# Transcriptomics Comparison between Porcine Adipose and Bone Marrow Mesenchymal Stem Cells during *In Vitro* Osteogenic and Adipogenic Differentiation

**DOI:** 10.1371/journal.pone.0032481

**Published:** 2012-03-07

**Authors:** Elisa Monaco, Massimo Bionaz, Sandra Rodriguez-Zas, Walter L. Hurley, Matthew B. Wheeler

**Affiliations:** 1 Laboratory of Stem Cell Biology and Engineering, Department of Animal Sciences, University of Illinois at Urbana-Champaign, Urbana, Illinois, United States of America; 2 Institute for Genomic Biology, University of Illinois at Urbana-Champaign, Urbana, Illinois, United States of America; University of Udine, Italy

## Abstract

Bone-marrow mesenchymal stem cells (BMSC) are considered the gold standard for use in tissue regeneration among mesenchymal stem cells (MSC). The abundance and ease of harvest make the adipose-derived stem cells (ASC) an attractive alternative to BMSC. The aim of the present study was to compare the transcriptome of ASC and BMSC, respectively isolated from subcutaneous adipose tissue and femur of 3 adult pigs, during *in vitro* osteogenic and adipogenic differentiation for up to four weeks. At 0, 2, 7, and 21 days of differentiation RNA was extracted for microarray analysis. A False Discovery Rate ≤0.05 for overall interactions effect and P<0.001 between comparisons were used to determine differentially expressed genes (DEG). Ingenuity Pathway Analysis and DAVID performed the functional analysis of the DEG. Functional analysis of highest expressed genes in MSC and genes more expressed in MSC vs. fully differentiated tissues indicated low immunity and high angiogenic capacity. Only 64 genes were differentially expressed between ASC and BMSC before differentiation. The functional analysis uncovered a potential larger angiogenic, osteogenic, migration, and neurogenic capacity in BMSC and myogenic capacity in ASC. Less than 200 DEG were uncovered between ASC and BMSC during differentiation. Functional analysis also revealed an overall greater lipid metabolism in ASC, while BMSC had a greater cell growth and proliferation. The time course transcriptomic comparison between differentiation types uncovered <500 DEG necessary to determine cell fate. The functional analysis indicated that osteogenesis had a larger cell proliferation and cytoskeleton organization with a crucial role of G-proteins. Adipogenesis was driven by PPAR signaling and had greater angiogenesis, lipid metabolism, migration, and tumorigenesis capacity. Overall the data indicated that the transcriptome of the two MSC is relatively similar across the conditions studied. In addition, functional analysis data might indicate differences in therapeutic application.

## Introduction

Reports of successful use of bone marrow derived mesenchymal stem cells (BMSC) in tissue engineering applications and disease treatments [1], [2], in addition to concerns about the use of embryonic stem cells, have stimulated increased interest in the use of adult stem cells for therapeutic purposes. Interestingly, among adult stem cells, the mesenchymal stem cells (MSC) are featured with several therapeutic properties, which make them excellent candidates for tissue replacement therapies. The MSC are able to differentiate into multiple cell lineages [3], secrete several factors (growth factors and cytokines) with important functions in tissue regeneration [4], are immune privileged [5], and secrete immunomodulatory factors [6], [7].

Mesenchymal stem cells were originally isolated from bone marrow [8], but they appear to be present in many tissues. One of the most interesting tissues for the isolation of MSC is adipose. The quantity and accessibility of subcutaneous adipose tissue in humans makes it an attractive alternative to bone marrow as a source of adult stem cells [9]–[11]. Reports of successful isolation and differentiation of adult stem cells derived from human adipose tissue have stimulated further studies regarding the ubiquity, similarity, and multipotency of these cells in comparison with BMSC [11]–[15].

The human-derived adipose stem cells can be easily studied *in vitro* but, for the *in vivo* studies, an appropriate animal model is needed. The pig is an ideal animal model for initial studies exploring human therapeutic applications. For instance, pigs are immunologically and physiologically more similar to humans than other non-primate species [16]. Previous *in vitro* studies reported in the scientific literature, or done in our laboratory, demonstrated that porcine adipose derived stem cells (ASC) can be easily harvested, isolated, expanded and differentiated *in vitro* [11], [13], [17], [18].

We have previously characterized the morphological features of porcine BMSC and ASC during adipogenic and osteogenic differentiation [18]. That study clearly demonstrated that both MSC are able to differentiate into adipocytes and osteocytes, but with some morphological differences. During the osteogenic differentiation large nodules are formed by ASC in contrast to a sheet of small nodules generated by BMSC. In the same study we analyzed the temporal expression of few osteogenic and adipogenic markers. In a further study, using the same conditions except that the MSC were cultivated in 3D hydrogel, no morphological differences were observed in the osteogenic differentiation between ASC and BMSC [17].

Some differences at the transcription level [19] and differentiation capacity [20] between BMSC and ASC have been previously reported in humans and mice. Besides those reported differences and the morphological differences seen in our 2D study during the osteogenic differentiation [18], the two MSC seem to be nearly identical when considered in the context of clinical applications [19], [21]. However, a more in depth comparison between BMSC and ASC is required in order to discover potential differences useful in a clinical setting. Analysis of the transcriptome can uncover main genes and associated functions that participate in determining the potential differences between the two MSC during differentiation. Few studies have been performed with human MSC where a transcriptomics comparison between BMSC and ASC has been reported, and none of those were longitudinal studies [19], [22].

The aim of the present study was to compare the transcriptome of porcine ASC and BMSC prior and during *in vitro* osteogenic and adipogenic differentiation. The results from this study uncovered that the transcriptome of the two MSC were similar before differentiation and relatively similar during differentiation. Functional analysis revealed an overall greater lipid metabolism in ASC, while BMSC had a greater cell growth and proliferation during differentiation. A relatively large number of genes differentially expressed were observed between differentiation types with osteogenesis featured by large cell proliferation, while adipogenesis was driven by PPAR signaling and was featured by an active angiogenesis, lipid metabolism, and migration.

## Results and Discussion

Reported in the present manuscript are main findings. Additional results and discussion are reported in file S1 and complete dataset in file S2.

### Highly abundant transcripts and MSC vs. fully differentiated tissues

The complete results from the highly expressed genes in MSC and DEG between MSC and fully differentiated cells are discussed in details in file S1 and reported in file S3 and S4. Among the most interesting findings were that highly abundant transcripts in MSC were significantly enriched in functions related to protein synthesis and with roles in extracellular matrix formation, differentiation, immune suppression, and cell cycle regulation. In addition, the comparison between MSC and fully differentiated tissues indicated that genes low expressed in MSC vs. differentiated tissues significantly enriched functions related to immune systems response including also major histocompatibility complex. All of these data reinforce the fact that MSC are immune-privileged, and this appears to be a feature of the MSC and likely regulated at the transcriptomic level.

### Transcriptome comparison prior to in vitro differentiation

#### Correlation analysis

**Figure 1:**
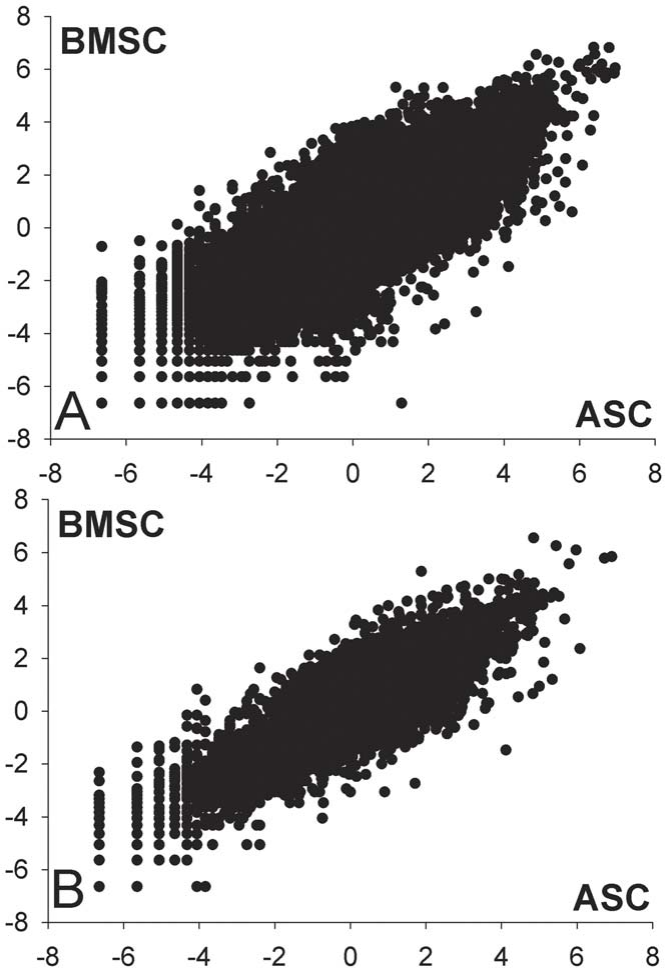
Overall correlation of the transcriptome between ASC and BMSC. Correlation of the transcriptome between ASC and BMSC during the whole experiment (top panel; r = 0.78; p<0.0001) or prior differentiation (bottom panel; r = 0.79; p<0.0001). This analysis highlights the large similarity between the two cell types both prior differentiation and during osteogenic or adipogenic differentiation.

The coefficient of correlation prior to differentiation (dd0) between the complete measured transcriptome of ASC and BMSC was 0.79 (p<0.0001; Figure 1 and Table S1) while the correlation among DEG was 0.63. When correlations between the transcriptome of ASC and BMSC at dd0 were run for each pig (Table S1) we observed variations among animals (r from 0.70 to 0.90). A similar animal variation was observed in the correlation between the transcriptome of ASC and BMSC during differentiation (Table S1). The three pigs used were of the same breed, same age, similar weight, and were raised in the same environment; in addition, the protocol for stem cells extraction and culture was identical between the three pigs. The inter-animal variation observed might be explained by animal genetics or by epigenetics factors. To our knowledge there is not available information in the literature about transcriptome correlation among ASC and BMSC in pigs. However, our observation appears to be in contradiction with previously reported data of high similarity between samples found in the transcriptome correlation between ASC and BMSC in humans using a relatively small microarray dataset [19]. The latter observation is also confirmed by analysis from another large microarray dataset [22], where the correlations ranged from 0.954 to 0.963 (Figure S1). Those data are surprising considering the large genetic and epigenetic variation among humans compared to specific pig breeds.

#### Number of DEG

Using a post-hoc correction of P<0.001, 65 DEG (64 unique annotated genes or 0.5% of all oligos coding for genes in array) were found between BMSC and ASC at dd0 with 40 unique annotated genes over-expressed in ASC *vs.* BMSC (see file S5, Sheet 1). The percentage of DEG compared to the measured transcriptome between the two cell types found in this experiment is lower than that previously reported in humans [19]. Microfibrillar associated protein 5 (17-fold; *MFAP5*), fibulin 2 (13-fold; *FBLN2*), periostin or osteoblast specific factor (8-fold; *POSTN*), secreted frizzled-related protein 2 (7-fold; *SFRP2*), and RNA 28S ribosomal 1 (7-fold; *RN28S1*) were the genes with >4-fold greater expression in ASC compared to BMSC. Interestingly the *MFAP5*, *FBLN2*, and *POSTN* are all extracellular matrix proteins, with specific roles in bone formation [23]–[25], and *POSTN* appears to play a role in tumorigenesis [25]. The *SFRP2* is a modulator of Wnt signaling pathways and appears to induce, through the signaling of the Wnt pathway, cell growth [26] and osteogenesis [27]. Overall, the data suggest a specific osteogenic-signature in undifferentiated ASC compared to BMSC. Osteopontin (7-fold; *SPP1*) was the only gene expressed at a level >4-fold in BMSC *vs.* ASC. The latter observation was confirmed by qPCR analysis (file S2). *SPP1* has been first isolated from bone where it is highly abundant and participates actively in osteogenesis [28]. The significance of the higher abundance of *SPP1* in BMSC compared to ASC is not clear. Other genes over-expressed (>2-fold) in BMSC *vs.* ASC were: myelin protein zero (*MPZ*), high mobility group AT-hook 1 (*HMGA1*), vascular endothelial growth factor A (*VEGFA*), fibronectin type III domain containing 3A (*FNDC3A*), and fibronectin 1 (*FN1*). The *MPZ* has important roles in myelination of peripheral nerves and cell adhesion [29]. The *HMGA1* is highly expressed in adenocarcinoma and appears to increase cancer invasiveness [30]. The *VEGFA* is a pivotal factor in angiogenesis [31]. Fibronectin proteins have an important role in extracellular matrix formation, especially during osteogenesis [32]. Overall, the genes with greater expression in BMSC *vs.* ASC suggest the former has a greater capacity for adhesion and migration into the tissues. Moreover, if *VEGFA* is elevated in BMSC *in vivo* compared to ASC, then BMSC may have a greater angiogenic capacity compared to ASC, which appears to challenge the well-known higher angiogenic capacity of adipose tissue compared to BMSC [21], [33]. In accordance with our findings, it has been demonstrated that BMSC have angiogenic capacity, at least in tumors [34], and *VEGF* has been reported to be more expressed in BMSC compared to ASC in humans [19]. On the other hand, we have observed a larger expression of *VEGFA* in ASC *vs.* BMSC during adipogenesis (see below).

A direct comparison of the gene expression in this current study was made with genes previously reported [19] to have significantly higher expression in human ASC *vs.* BMSC (8 genes) and in BMSC *vs*. ASC (29 genes). Among the 37 genes reported in the human study only 4 were present as DEG in our analysis, with *VEGFA* and wingless-type MMTV integration site family member 5A (*WTN5*) confirmed to be more expressed in BMSC *vs.* ASC. In addition, the analysis confirmed a greater expression of *SFRP2* in ASC *vs.* BMSC. The fibronectin 1 was among the genes with a larger expression in ASC *vs.* BMSC in the human comparison [19] but was among the genes with greater expression in BMSC *vs.* ASC in the current study of porcine cells. The different results may highlight a likely species-specific difference.

#### Functional enrichment analysis

Several functions were identified by using IPA with a minimum of 2 genes as significantly overrepresented in DEG between BMSC and ASC at dd0 (Table 1; supplementary details in file S5). Several functions were found significantly enriched in the 24 DEG more expressed in BMSC *vs.* ASC, and only few were enriched in the 40 DEG more expressed in ASC *vs.* BMSC. It is interesting that most of the DEG in the significantly enriched functions were the ones with the greatest difference in expression between the types of cells, particularly for the genes more expressed in BMSC *vs.* ASC. This may suggest that the enriched functions were highly impacted.

**Table 1:** Functions significantly enriched (Benjamini-Hochberg FDR ≤0.05) in DEG between ASC and BMSC at dd0 (or prior differentiation).

Cell type^1^	Category	P-value^2^	# Genes	Effect on function (all induced)^3^
**ASC**	Skeletal and Muscular System Development and Function	0.023	2	Myogenesis
	Tissue Development	0.023	3	Tissue development
**BMSC**	Cellular Movement	0.004	5	Cell migration and invasion
	Immune Cell Trafficking	0.004	9	Migration of immune cells and activation of T-lymphocytes
	Cell-To-Cell Signaling and Interaction	0.006	8	Activation of T-lymphocytes and adhesion of fibroblasts
	Tissue Development	0.006	3	Cell adhesion
	Connective Tissue Development and Function	0.006	6	Adhesion and growth of fibroblasts
	Cellular Assembly and Organization	0.006	13	Outgrowth of neurites, quantity of focal adhesion, and rearrangement of the cytoskeleton

1 ASC denotes genes more expressed in ASC *vs.* BMSC while BMSC denotes genes more expressed in BMSC *vs.* ASC.

2 The P-value denotes the Benjamini-Hochberg FDR corrected P-value as provided by Ingenuity Pathway Analysis. Additional functions significant with a non-corrected P-value ≤0.05 are reported in online file S5.

3 The effect on function is estimated by the “effect on function” option in IPA using criteria as described in supplementary Materials and Methods in file S1. The “all induced” indicates that all the reported functions were overall induced in the DEG more expressed in one stem cell type *vs.* the other.

The number of genes of significant enriched functions in DEG more expressed in ASC compared to BMSC was very low (2 and 3, see Table 1), but the number of genes was relatively low also for enriched functions in additional comparisons. Thus, the interpretation of those functions needs to be taken *cum grano salis*. The enrichment analysis is highly dependent on the dimension of the gene list [35]: smaller is the gene list higher is the likelihood of finding significant enriched functions. Despite this limitation, and in order to be consistent, we have set for all the comparisons a threshold of significance for discussion of ≥2 genes per function, as for the default in DAVID [36].

Considering what reported above, the results by IPA uncovered a high enrichment in DEG more expressed in ASC vs. BMSC of terms related to tissue development. In particular, the analysis of effect on functions in IPA (file S5, Sheets 4 and 5) identified myogenesis as the most enriched and induced function (Table 1). Functions enriched significantly among the genes over-expressed in BMSC *vs.* ASC included cell movement and migration, bone formation, angiogenesis (e.g., cardiovascular system development), and nervous system development (Table 1 and file S5, Sheets 8). Moreover, the analysis uncovered a significant enrichment of functions related to metabolism among the DEG that were more expressed in BMSC *vs.* ASC (e.g., carbohydrate, gene expression, molecular transport, DNA metabolism, and protein synthesis). The functional analysis suggested an overall greater capacity of the undifferentiated BMSC *vs.* undifferentiated ASC for migration, cell adhesion, inflammatory response, and binding of carbohydrates (file S5, Sheet 9). There were not significantly enriched pathways in IPA analysis in the two gene lists.

The results from DAVID confirmed (but only with an EASE score <0.1) the enrichment of genes involved with muscle development in DEG with greater expression in ASC *vs.* BMSC (file S5, Sheet 2 and 3), and enrichment of genes involved in metabolism (particularly nitrogen and purine metabolism and secretion) and adhesion in DEG with greater expression in BMSC *vs.* ASC (file S5, Sheet 6 and 7). The DAVID analysis additionally uncovered among the most enriched terms in DEG more expressed in ASC *vs.* BMSC extracellular matrix, actin binding, and several TFBS (file S5, Sheets 2 and 3). Among the latter, the most enriched were BRACH, which represents the site for the binding of Brachyury protein (29 DEG had binding motifs able to bind the TF), the heat shock transcription factor 2 (HSF2; 24 DEG with binding motifs for the TF), the sex determining region Y (SRY; 23 DEG with binding motifs for the TF), and regulatory factor ×1 (RFX1; 30 DEG with binding motifs for the TF). The BRACH protein has been shown to be important during the differentiation in chondrocytes of MSC [37]. The HSF2 protein is important in neuronal development, particularly in cortex development [38], [39]. The SRY is important in the determination and development of testis [40]. The RFX1 is known to regulate MHC class II expression [41], to affect spermatogenesis [42], and to indirectly increase neuronal differentiation through induction of *FGF1* expression [43]. These findings suggest a higher chondrogenic, neurogenic, and spermatogenic differentiation capacity of the ASC compared to BMSC. Indeed it has been shown that both MSC are able to restore male fertility [44], differentiate to chondrocytes [45], or induce differentiation of neurogenic lineages [46], [47] likely through release of paracrine factors.

The most enriched terms from the DAVID analysis of the DEG with higher expression in BMSC *vs.* ASC at dd0 were related to nitrogen compound metabolism, vesicle transport, fibronectin, and bone resorption and remodeling (file S5, Sheet 6 and 7). However, the number of genes was low (from 2 to 5) to provide a biologically relevant conclusion. The most enriched TFBS was ARP1 with 15 DEG presenting binding sites able to bind the TF. This TF is known to be highly expressed in human BMSC [48] with multiple functions. Some of those functions, for instance, are involved in liver development [49], brain development [50], and normal embryonic development [51]. Those data indicate that BMSC are probably more prone to differentiate into liver or pancreas compared to ASC. However, this inferred conclusion needs to be verified by *in vitro* and/or *in vivo* data.

Overall data indicated that the two undifferentiated MSC have substantial similarity at the transcriptome level, with ASC having a greater expression of most DEG compared to BMSC. The transcriptome differences between these MSC may relate to a slight diversity in differentiation capacity.

### Overall DEG between ASC and BMSC

Details of results and discussion of DEG between ASC and BMSC during the entire experiment (cell type effect with FDR P-value ≤0.05) with relative functional analysis are reported in file S1, S7 and S9.

### Transcriptome comparison between in vitro osteogenic and adipogenic differentiation in ASC and BMSC

#### Correlation analysis

The overall measured transcriptome correlation between the osteogenic and adipogenic differentiation was positive (0.78; P-value <0.0001). This value was surprisingly not different than when the same analysis was run for the dd0 only. Correlation considering only the DEG with overall FDR ≤0.05 was 0.70. When the correlation was run for each cell type the ASC showed a higher correlation (r = 0.80) compared to BMSC (r = 0.74). Among time points, day of differentiation (dd) 7 had the lowest correlation. There was a variation in transcriptome correlation of osteogenic and adipogenic differentiation among animals (Table S1).

#### Number of DEG

**Figure 2:**
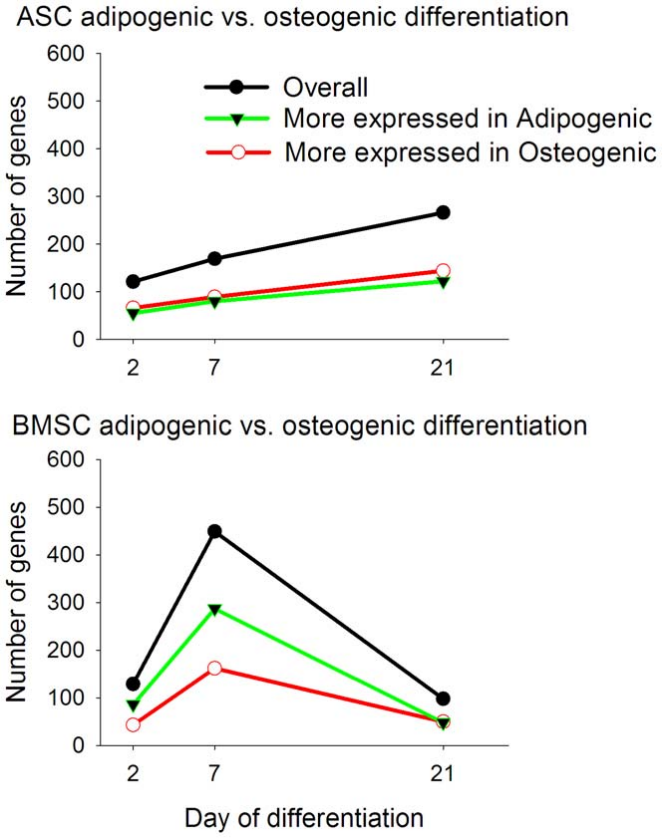
Number of transcripts differentially expressed in adipogenic *vs.* osteogenic differentiation in ASC and BMSC. The figure reports also the number of genes highly expressed in adipogenic *vs.* osteogenic differentiation (green line) and osteogenic *vs*. adipogenic differentiation (red line).

The number of DEG between the adipogenic and osteogenic differentiation was relatively low in ASC, with the lowest number of DEG observed at dd2, followed by an increase up to dd21 (Figure 2). The number of DEG that had greater expression in adipogenic *vs.* osteogenic differentiation was similar to the number of DEG that had greater expression in osteogenic *vs.* adipogenic differentiation.

In BMSC, the number of DEG between the two differentiations appeared to be similar to the ASC at dd2, which was followed by a large increase at dd7 and subsequently followed by a decrease at dd21. In addition, adipogenic differentiation in BMSC induced the expression of twice as many genes as compared to the osteogenic differentiation at dd7. Surprisingly, and differently from the ASC, the BMSC fully differentiated in bone or adipose (dd21) had a more similar transcriptome than the two differentiating cells at dd7 or even at dd2 (Figure 2). In BMSC there was an overall greater number of genes with a greater expression in the adipogenic differentiation compared to the osteogenic differentiation (Figure 2).

Transcriptome dissimilarity between differentiations was greater in BMSC compared to ASC, with a particularly greater difference at dd7 (Figure 2). The overall data suggest that the two MSC behave in a different way at the transcriptomics level during these *in vitro* differentiations, in particular at the end of the first week. The morphological characterization of the two differentiations [18] demonstrated a clear adipogenic and osteogenic differentiation for both MSC. Therefore, the relatively small transcriptomics difference between adipocytes and osteocytes (at dd21) from BMSC is surprising. In addition, from the results it is clear that for both cell types, but in particular for the ASC, only a limited number of DEG appeared to be necessary to drive the MSC to differentiate to bone or adipose.

#### Functional enrichment analysis by IPA

**Figure 3:**
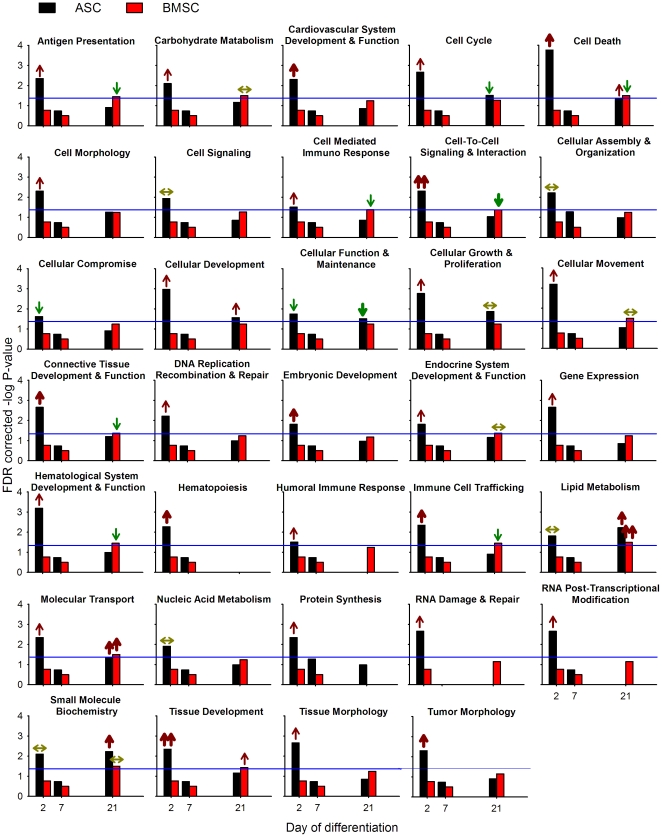
Functional analysis of differentially expressed genes between adipogenic and osteogenic differentiation in ASC and BMSC. Ingenuity Pathway Analysis® (IPA) of ASC (black bar) and BMSC (red bar) induced to differentiate towards the adipogenic and osteogenic lineages. The blue line denotes an FDR of 0.05. The arrows present at the top of each bar denote the overall effect on the function inferred by the gene annotation using IPA (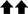 = highly activated in adipogenic *vs.* osteogenic differentiation; 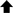 = activated in adipogenic *vs.* osteogenic differentiation; ↑ = tends to be activated in adipogenic *vs.* osteogenic differentiation; 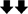 = highly activated in osteogenic *vs.* adipogenic differentiation; 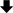 = activated in osteogenic *vs.* adipogenic differentiation; ↓ = tends to be activated in osteogenic *vs.* adipogenic differentiation; ↔ = not apparent overall activation or inhibition).

Results of the functions found to be significantly enriched by IPA with a B–H FDR ≤0.05 of the DEG between adipogenic and osteogenic differentiation in ASC and BMSC at each time point are reported in Figure 3 and Tables S2, S3, and S4. The results of DAVID analysis for each single time point comparison are reported in file S6.

Several functions were significantly enriched in at least one time point comparison (Figure 3 and Tables S2, S3, and S4). The DEG between the two differentiations in ASC had the greatest number of significantly enriched functions, particularly at dd2. The DEG between differentiation in ASC did not present significant enriched functions at dd7, and presented only 8 enriched functions at dd21 (Figure 3). In BMSC, at dd2 and dd7 there were no significant overrepresented functions despite the high number of DEG compared to dd21 (Figure 2 and 3). In contrast, several (n = 14) functions were significantly enriched at dd21 for BMSC (Figure 3 and Table S4). The lack of enriched functions with the selected criteria (i.e., B–H FDR ≤0.05) in the comparison at dd7 in ASC and at dd2 and dd7 in BMSC between differentiations, which corresponded to the larger gene size among comparisons, was probably due to a dependence of the enrichment analysis on the gene list size as above reported and previously pointed out [35].

The results of IPA in DEG during each time point of differentiations for each cell type are summarized below as follows:

At dd2 in ASC the DEG between differentiations presented a large number of significantly enriched functions (Figure 3). In contrast, there were not functions or pathways enriched in BMSC. Using the “effect on function” analysis in IPA (Table S2) an overall greater induction of almost all the significantly enriched functions was uncovered for adipogenesis compared to osteogenesis. Tissue development and cell-to-cell signaling and interaction were the most induced functions in adipogenesis compared to osteogenesis (Figure 3). There was greater induction of cell adhesion in adipogenesis compared to osteogenesis in ASC with particular enrichment of molecules involved in adhesion of endothelial and immune cells, and recruitment of cells (Table S2). Other enriched functions in the comparison of adipogenesis *vs.* osteogenesis in ASC were cell death, formation of connective tissue, immune cell trafficking, cardiovascular system development and function, tumor morphology, and hematopoiesis. The interpretation of those functions by the “effect on function” analysis in IPA suggest that in ASC adipogenesis induced, compared to osteogenesis, a higher apoptosis (with a likely larger cell cycle as well), larger migration and cell adhesion, larger angiogenic capacity, and greater effect on formation and morphology of tumors (Table S2). The higher apoptosis is supported by a significant decrease in number of cells during adipogenesis *vs.* osteogenesis in ASC (Figure S2). It is also supported by time lapse visualization of the cells during the first 24 h after initiation of differentiation (video S1, S2, S3, and S4).

The higher induced angiogenesis during adipogenic differentiation compared to the osteogenic is interesting. The ASC have been shown to be able to differentiate into endothelial cells and participate into the formation of blood vessels in mouse model [52], [53]. Our data suggest that during the beginning of adipogenesis there is stimulation for angiogenesis. This appears to be in line with the evidence that vasculogenesis or angiogenesis regulates adipogenesis (reviewed in [54]). However, for other IPA results observed no supporting data are available because there is a paucity of studies comparing temporal osteogenesis and adipogenesis in mesenchymal stem cells. Beside the functions clearly more activated, other functions were slightly more activated during adipogenesis compared to osteogenesis in ASC (Figure 3). Among those it is interesting to highlight carbohydrate and lipid metabolism. In particular the “effect on function” analysis indicated a larger uptake of glucose by the cells, and a lower phosphatidylcholine and triacylglycerol hydrolysis during adipogenesis compared to osteogenesis. Interestingly, the phosphatidylcholine is potentially involved in the control of cell growth as a mitogenic factor [55]. The overall data indicate that ASC undergoing adipogenesis are more prone to migrate, undergo apoptosis, induce/participate in angiogenesis, and participate in tumor formation than when undergoing osteogenesis. Moreover, these cells present a larger uptake of glucose and a reduced hydrolysis of lipids which might allowed for a larger triacylglycerol accumulation observed as early as dd2 in few cells, but evident at dd4 [18].

At dd21 in ASC the DEG between differentiations presented several functions that were significantly enriched (Figure 3 and Table S3). Lipid metabolism (particularly triacylglycerol formation) was more induced during adipogenesis compared to osteogenesis (see also molecular transport and small molecule biochemistry in Table S3). Cellular function and maintenance (particularly function of the cytoskeleton) was more enriched in DEG more induced during osteogenesis compared to adipogenesis (Table S3). The analysis suggests that at dd21 the ASC undergoing adipogenesis had greater cell death and lower cell cycle function compared to those undergoing osteogenesis (Table S3). Overall the data indicate a clear lipidogenic feature of the cells undergoing adipogenesis compared to osteogenesis, and a more pronounced cell morphology transformation during osteogenesis. Those features are supported by the *in vitro* morphological characterization of the same cells [18].

At dd21 in BMSC, as was the case for the ASC, the synthesis and accumulation of lipids were among the most enriched functions and were induced in DEG between adipogenesis and osteogenesis (Figure 3 and details in Table S4). Cell cycle and cell death were more pronounced in osteogenesis *vs.* adipogenesis with a likely greater overall cell proliferation at dd21. This is supported by a significant greater number of cells in BMSC undergoing osteogenesis compared to adipogenesis (Figure S2). In contrast to the ASC, the osteogenic differentiation in BMSC had a greater cell-to-cell signaling and interaction (particularly the activation of cells) and antigen presentation compared to the adipogenic differentiation. Other functions were either not more induced during one differentiation compared to the other or were not significantly enriched. As for ASC, the overall data clearly confirmed the *in vitro* observations [18]. In addition, the data uncovered a greater immunogenicity in BMSC undergoing terminal osteogenesis compared to adipogenesis (Table S4).

#### Functional enrichment analysis by DAVID

Using DAVID functional analysis several terms were significantly enriched (FDR P-value ≤0.05) at each time point by DEG between differentiations, with a greater number of significantly enriched terms in ASC compared to BMSC (file S6). The analysis uncovered as the most significant enriched functions in DEG those related to dd2, dd7, and dd21.

At dd2 the data indicated increasing proliferation and decreasing apoptosis and stimulation of biosynthetic processes involving mitochondria, among DEG that were more expressed during adipogenesis *vs.* osteogenesis in ASC. Binding of platelet-derived growth factor and zinc finger protein 238, involved in chromatin assembly (*RP58*), and the neuron-specific transcription factor OLF1 as TFBS, were significantly enriched in DEG with greater expression during osteogenesis *vs.* adipogenesis in BMSC. At dd7 protein synthesis and acetylation were the most enriched functions among DEG that were more expressed during adipogenesis *vs.* osteogenesis in both ASC and BMSC, and GTPase activity in DEG that were more expressed during osteogenesis *vs.* adipogenesis in ASC and BMSC. At dd21 fatty acid metabolic processes involving NADP and PPAR signaling were among the most enriched functions in DEG more expressed during adipogenesis *vs.* osteogenesis in ASC. Methylation, cytoskeleton organization and cell division were the most enriched functions in DEG more expressed in osteogenesis *vs.* adipogenesis in ASC. In BMSC, the data indicated a significant enrichment of functions related to lipid biosynthesis, particularly sterol biosynthesis, and PPAR signaling in DEG that were more expressed in adipogenesis *vs.* osteogenesis.

#### Overall pathway enrichment

Among more than 200 pathways present in IPA there were 15 that were significantly (FDR P-value ≤0.05) enriched in at least one time point among the DEG between the adipogenic and osteogenic differentiation in ASC and BMSC (Figure S3). None of the pathways were significantly overrepresented in DEG between differentiations at dd2. Five and 12 pathways were significantly enriched in DEG between differentiations in ASC at dd7 and dd21, respectively (see file S7). Three of those pathways were enriched in DEG between differentiations in both dd7 and dd21: CCR5 signaling in macrophages, cardiac hypertrophy signaling, and α-adrenergic signaling (Figure S3). Those three pathways have in common the signaling from G-proteins and calmodulin groups, both more expressed in osteogenic *vs.* adipogenic differentiation (file S7). The CCR5 pathway is involved in chemotaxis, the cardiac hypertrophy pathway is involved in cell proliferation, and the α-adrenergic pathway plays an important role in the regulation of adipose tissue lipolysis [56], among others. IL8 and relaxin signaling were significantly enriched only in DEG between differentiations in ASC at dd7. As for the above pathways, also for IL8 and relaxin, an important role is played by G-proteins (file S7). The G-proteins group appears to be an important hub in the ASC decision to move toward adipogenesis or osteogenesis. The small G-proteins are a superfamily of proteins with a myriad of roles; with minor variations in GTP/GDP-binding core domain they have a role in nearly every known signaling process in cells including vesicular targeting, nuclear transport, and growth factor signaling for maintenance of the spatial cycles [57]. Our data appear to be supported by previous results, where the down-regulation of a gamma 5 G-protein was essential for the onset of adipogenic differentiation [58].

The other pathways enriched significantly in DEG between the two differentiations in ASC at dd21 (Figure S3) included pathways involved in cellular movement, cytoskeleton signaling, and fatty acid biosynthesis. The first two categories of pathways were more induced in osteogenesis compared to adipogenesis (see depiction of the pathways in file S7). As for the pathways discussed above, those pathways have in common the signaling involving CCR receptors and G-proteins. In particular, the CCR3 signaling was the most significantly enriched pathway (Figure S3, file S7). The activation of CCR3 receptor by chemokines, besides stimulating intracellular calcium release and production of active oxygen species, produces changes in actin polymerization allowing the chemotaxis of eosinophils. Human CD34^+^ cord blood progenitor cells and CD34^+^ progenitors from bone marrow express the CCR3 receptor [59], [60]. Moreover, CCR3-dependent chemokine interactions control migration of CD34^+^ progenitors from bone marrow to sites of injury such as ischemic myocardium [60]. In our case, the pathway appeared to be more induced during osteogenesis compared to adipogenesis.

The detail visualization of the chemokine signaling pathway (file S7) allowed uncovering a greater expression of *CXCR4* gene during adipogenesis compared to osteogenesis in ASC. This gene codes for a receptor that specifically binds SDF-1 [61]. The interaction of another chemokine receptor, the CXCR4, with SDF-1 is known to be important in hematopoietic stem cell homing and quiescence [62], [63]. Moreover, it has been shown that *in vitro* over-expression of CXCR4 induces human ASC migration in response to SDF-1 [64]. In another *in vitro* study of ASC, the interaction of CXCR4, expressed by ASC, with its ligand SDF-1 induced ASC chemotaxis [65]. The SDF-1 is secreted by adipose-derived capillary endothelial cells. Our data suggest that ASC undergoing adipogenic differentiation may be less sensitive to chemokines that bind CCR3 but more sensitive to those that bind CXCR4 compared to ASC cells undergoing osteogenic differentiation.

Other pathways related to cell motility/cytoskeleton signaling in adipogenic *vs.* osteogenic differentiation in ASC at dd21 induced ‘ephrin receptor signaling’ and ‘fMLP signaling in neutrophils’ (Figure S3, file S7). Ephrin receptor signaling can affect cell migration by modifying cytoskeletal organization and cell adhesion [66]. It also seems that this receptor signaling can influence numerous biological processes such as cell proliferation and fate [66], cell-cell interaction, morphology and angiogenesis [67]. The fMLP signaling has been related to the chemotaxis of phagocytic leukocytes towards inflammatory sites [68].

Overall results from IPA support a greater capacity for motility and cytoskeleton modification during osteogenesis compared to adipogenesis in ASC. This is consistent with our *in vitro* observations [18] where ASC undergoing osteogenic differentiation moved to form large cell clusters to produce columnar-shaped nodules. This behavior was not observed during adipogenesis nor was observed in BMSC during either differentiation. For BMSC, only one pathway (LPS/IL1 mediated inhibition of RXR function) was found to be significantly enriched by the DEG between differentiations at dd21 by IPA (file S7). The same pathway was enriched for the ASC, as well (Figure S3). In both cases the pathway was more induced during adipogenesis compared to osteogenesis mostly as a result of a greater expression of genes coding for proteins involved in lipid metabolism.

In DAVID few Kyoto Encyclopedia of Genes and Genomes (KEGG) pathways were significantly enriched (FDR P-value ≤0.05) (Table 2). Among those, ribosome (dd7) and PPAR signaling (dd7 and dd21) were enriched in DEG that were over-expressed in adipogenic *vs.* osteogenic differentiation in both MSC. At dd2 the DEG more expressed in adipogenic *vs.* osteogenic differentiation in ASC significantly enriched pathways involved in inflammation and cancer (Table 2). At dd21 the DEG more expressed in BMSC undergoing adipogenesis *vs.* osteogenesis enriched significantly steroid biosynthesis, as well as PPAR signaling. Those data indicate that PPAR signaling is the most important pathway in differentiating cells toward adipocytes. This finding is not novel but confirms the fact that PPAR activation, in particular PPARγ, is essential for adipogenesis [69]. Moreover, this analysis clearly identifies PPAR signaling as one of the most important pathways that distinguish differentiation of cells toward adipogenesis *vs.* toward osteogenesis. This conclusion is supported by previous data showing that mouse homozygous PPARγ-deficient ES cells failed to differentiate into adipocytes, but spontaneously differentiated into osteoblasts [70]. Besides PPAR signaling, our data highlighted a crucial role of the protein synthesis machinery (i.e., ribosome) in driving MSC to differentiate into adipocytes instead of osteocytes (Table 2). In both MSC the enrichment of DEG associated with ribosomes (i.e., protein synthesis) happened within one week of the *in vitro* differentiation process. It is interesting that the quantity of RNA/cell (Figure S4) had a significant increase during the first week of differentiation, particularly during osteogenesis.

**Table 2:** KEGG pathways significantly enriched with a Benjamini-Hochberg FDR ≤0.10 in DAVID analysis for DEG between adipogenesis and osteogenesis in ASC and BMSC.

Pathway	# genes	p-value	Benjamini
***DEG more expressed in adipogenic vs. osteogenic differentiation***			
**ASC**			
**dd2**			
hsa04621:NOD-like receptor signaling pathway	4	0.002	0.059
hsa04060:Cytokine-cytokine receptor interaction	6	0.001	0.069
hsa05200:Pathways in cancer	7	0.004	0.080
**dd7**			
hsa03010:Ribosome	10	<0.001	<0.001
hsa03320:PPAR signaling pathway	5	0.004	0.100
**dd21**			
hsa03320:PPAR signaling pathway	7	<0.001	0.012
**BMSC**			
**dd7**			
hsa03010:Ribosome	13	<0.001	<0.001
**dd21**			
hsa00100:Steroid biosynthesis	4	<0.001	0.003
hsa03320:PPAR signaling pathway	5	0.003	0.006
***DEG more expressed in osteogenic vs. adipogenic differentiation***			
**BMSC**			
**dd7**			
hsa04810:Regulation of actin cytoskeleton	11	0.001	0.088

The DAVID analysis confirmed the significant enrichment of pathways involved in inflammation and cancer among DEG more expressed in adipogenesis *vs.* osteogenesis in ASC at dd2 first uncovered by the functional analysis in IPA (i.e., immune cell trafficking and tumor morphology, described above). The pathways indicated that the ASC during adipogenesis may have a greater anti-inflammatory behavior [higher expression of the inhibitor of NFκB (*IKBKG*)], and a greater secretion of multiple cytokine-like molecules with chemotactic and angiogenic effect (*CCL2*, *CCL5*, and *VEGFA*) compared to the osteogenic differentiation. Many of the same genes are also involve in pathways related to cancer. The results appear to indicate a greater tumorigenesis capacity for ASC during early adipogenesis compared to osteogenesis. However, tumor formation is not strictly a cellular phenomenon and the suggestion by those data needs to be taken with caution. The DEG involved in angiogenic capacity are all factors (i.e., *CCL2*, *CCL5*, and *VEGFA*) released by cells and known to affect positively the formation of vessels; thus, this last can be considered more likely as a direct cellular effect.

From IPA, fatty acid biosynthesis was significantly enriched in adipogenesis *vs.* osteogenesis only in ASC (Figure S3, file S7), consistent with a greater utilization of fatty acids for triacylglycerol formation in ASC. Interestingly, the IPA did not uncover the same enrichment in DEG between differentiations in BMSC; however, DAVID uncovered in both MSC the central role of PPAR signaling in guiding the MSC toward the adipogenic differentiation. In addition, the functional analysis using DAVID uncovered an important role of steroid biosynthesis for the formation of cholesterol among DEG more expressed in adipogenic *vs.* osteogenic differentiation in BMSC. The same functions were not revealed to be significantly enriched in IPA, probably due to different knowledge base compared to DAVID. However, this finding highlights the limitation on relying on a single bioinformatics tool in performing functional analysis of microarray data [35]. In summary, the data highlight the central role of PPAR signaling and differences between the two MSC. Moreover, IPA results might explain the greater overall lipid accumulation in ASC *vs.* BMSC previously observed [18].

### Transcriptome comparison between ASC and BMSC during adipogenic and osteogenic differentiation

#### Correlation analysis

In line with the above reported overall coefficient of correlation for the complete transcriptome between BMSC and ASC, the same analysis for each time point showed a significant (P-value<0.0001) and positive Pearson correlation between BMSC and ASC (0.79, 0.72, and 0.85, for dd2, dd7, and dd21, respectively). The overall correlation was higher for the osteogenic (r = 0.79) compared to the adipogenic (r = 0.76) differentiation (Table S1). The lower correlation seen for the adipogenic differentiation was due mostly to one animal that presented an overall correlation between the two cell types of r = 0.61 (Table S5). The same animal also had the lowest correlation during the osteogenic differentiation (Table S6). The overall correlation between pigs was lower for the BMSC compared to the ASC. The correlation considering only overall DEG (FDR ≤0.05) was 0.76. Overall those data highlight a large similarity in gene expression between the two MSC undergoing the osteogenic and adipogenic differentiation with some variation between animals, and with a more consistent transcriptome between animals in ASC compared to BMSC.

#### Number of DEG

**Figure 4:**
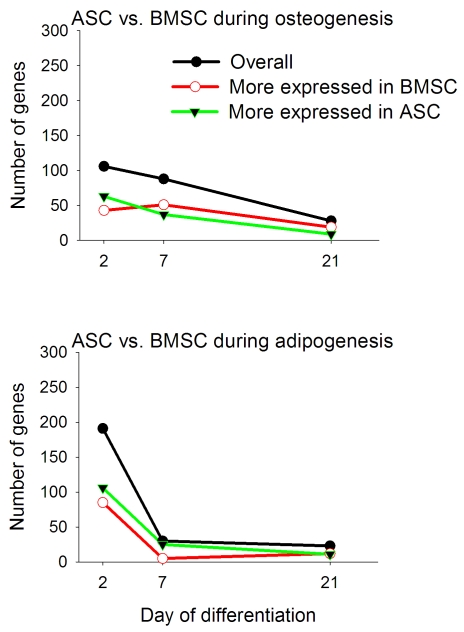
Number of transcripts differentially expressed in BMSC *vs.* ASC during adipogenic and osteogenic differentiation. Number of transcripts differentially expressed in BMSC *vs.* ASC in adipogenic and osteogenic differentiation at each time point. The image reports also the number of genes more expressed in BMSC *vs.* ASC (red line) or more expressed in ASC *vs.* BMSC (green line).

The number of DEG between BMSC and ASC at the beginning (dd2) of the *in vitro* differentiation was 191 and 106 for the adipogenic and osteogenic differentiations, respectively (Figure 4), but already at dd7 the number dropped to 30. During the osteogenic differentiation the number of DEG between BMSC and ASC continued to decrease over time with only 28 DEG between the two cell types at dd21 as it was for dd21 adipogenic differentiation (23 DEG). The data also showed that during the adipogenic differentiation ASC had more genes with a greater expression compared to BMSC until dd7 (Figure 4). For the osteogenic differentiation more genes with a higher expression in ASC *vs.* BMSC were present at dd2; afterwards, the genes with a higher expression in BMSC *vs.* ASC were relatively more numerous. These results, together with data at dd0 reported above, strongly support a relatively larger difference between the two MSC during the early phases of differentiation compared to late differentiation.

#### Overall functional enrichment analysis

**Figure 5:**
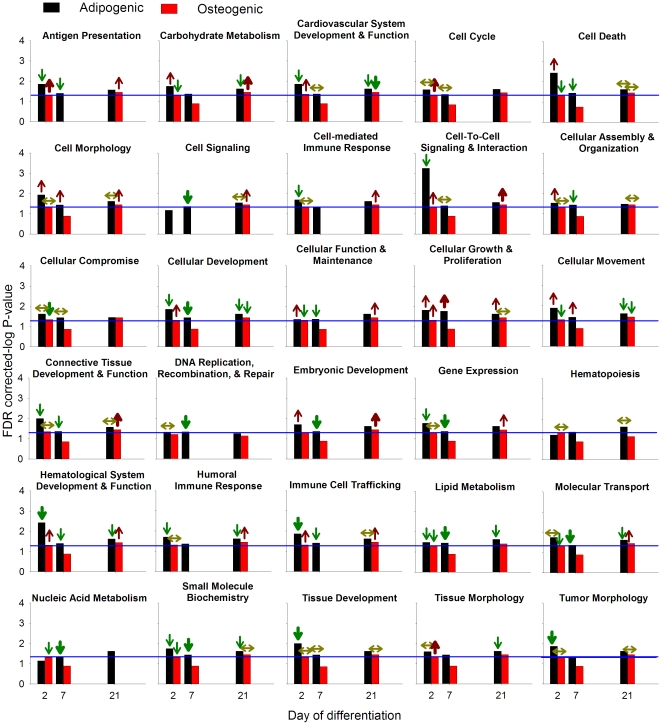
Functional analysis of differentially expressed genes between BMSC and ASC during adipogenic and osteogenic differentiation. Ingenuity Pathway Analysis® (IPA) of BMSC *vs.* ASC induced to differentiate towards the adipogenic (black bar) and osteogenic (red bar) lineages. The blue line denotes an FDR of 0.05. The arrows denote the overall effect on the function inferred by the gene annotation using IPA (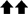 = highly activated in BMSC *vs.* ASC; 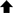 = activated in BMSC *vs.* ASC; ↑ = tends to be activated in BMSC *vs.* ASC; 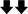 = highly activated in ASC *vs.* BMSC; 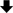 = activated in ASC *vs.* BMSC; ↓ = tends to be activated in ASC *vs.* BMSC; ↔ = not apparent overall activation or inhibition). Functions with “effect on functions” with only 1 gene were not considered for the analysis (i.e., not arrows; see Tables S7, S8, S9, S10 and S11 for details).

Significant enrichment of IPA functions (FDR P-value ≤0.05) by DEG between BMSC and ASC in adipogenic and osteogenic differentiation in at least one time point are reported in Figure 5. Different from the comparison between differentiations, IPA identified several enriched functions among the DEG between the two MSC. In contrast, the analysis with DAVID resulted in few terms significantly enriched (FDR P-value ≤0.05; file S8). To facilitate the interpretation of the enrichment analysis the functions are discussed in 6 clusters, as related to: immune function, metabolism, system development and function, cell cycle, signaling, and morphology.

For the immune function data indicated that all significantly enriched immune-related functions by DEG between BMSC and ASC in the adipogenic differentiation had a greater induction in ASC *vs.* BMSC (Figure 5 and Tables S7, S8, and S9), especially at dd2. Functions related to movement of immune-related cells were among the most important functions, with an indication of a greater induction in ASC *vs.* BMSC mediated through a potential greater release of chemotactic factors (e.g., *CXCL2* and *IL1A*). However, several other functions related to adhesion of immune-cells and inflammation also were more expressed in ASC *vs.* BMSC (e.g., *NFKB1A* and *VCAM1*). Interestingly, a larger induction of immune-related functions was observed in BMSC vs. ASC during the osteogenic differentiation (Figure 5 and Table S10, S11). Overall, the data suggest a slightly greater inflammatory response in the differentiation proper of each MSC (e.g., in adipogenesis for ASC and osteogenesis for BMSC). The biological meaning of this effect is not apparent. Moreover aside from dd2, where the number of DEG was relatively numerous, at the other dd the number of DEG in each function was relatively low (<3–4 genes) and the potential biological meaning may be insignificant.

Among the metabolic functions lipid metabolism was the most enriched. The effect on function analysis of DEG between BMSC and ASC during the adipogenic differentiation suggested a greater lipid metabolism (especially accumulation of lipids) in ASC *vs.* BMSC during the first week of adipogenic differentiation (Figure 5 and S7, S8, S9). Those data are in accordance with the greater lipid droplet formation observed in ASC *vs.* BMSC [18]. The analysis of DEG between BMSC and ASC during the osteogenic differentiation also suggested a greater lipid metabolism in ASC *vs.* BMSC at dd2 (Figure 5 and Table S10). These data appear to suggest that the ASC also tend to retain a greater lipid metabolism function compared to BMSC during the osteogenic differentiation. The analysis with DAVID of DEG more expressed during osteogenesis in BMSC *vs.* ASC at dd2 only uncovered enrichment among metabolic-related functions associated with steroid biosynthesis (file S8, Sheet 4).

Among the functions of the System Development and Function (SD&F) group, in addition to the hematological SD&F included in the above discussion of the immune-related functions, cardiovascular and connective SD&F were significantly enriched in almost all the comparisons (Figure 5). The effect on function analysis indicated a greater induction at dd2 for both adipogenesis and angiogenesis in ASC *vs.* BMSC during *in vitro* adipogenesis (Table S7). The greater adipogenesis response in ASC *vs.* BMSC is in line with previously observed morphological measurements [18]. A greater angiogenic response in ASC *vs.* BMSC was also suggested by the data at dd21 during osteogenic differentiation (Table S11). The ASC express the CD34 surface marker [19]. A positive correlation between CD34+ cells and *in vivo* angiogenesis has been reported in the rat [71]. The BMSC also actively enhance angiogenesis *in vivo* [72], but the ASC have a greater angiogenic capacity. Moreover, it appears that angiogenesis needs to precede adipogenesis in the normal adipose tissue development [33]. Other functions related to development (tissue, cell, and embryonic development) were significantly enriched, especially in early adipogenic differentiation. The effect on function analysis suggested a greater differentiation of the ASC in adipose compared to BMSC (Table S7, S8 and S9). Moreover, it also uncovered a greater differentiation of the BMSC compared to ASC towards bone tissue during early osteogenesis (Table S10).

Among the cell cycle functions four were the most enriched: cell cycle, cell death, cellular growth and proliferation, and DNA replication, recombination, and repair (Figure 5). The interpretation of the data using the effect on function in IPA (Table S7, S8, S9, S10, and S11) indicated that at dd2 cell death was higher in BMSC during adipogenesis and lower in BMSC during osteogenesis *vs.* ASC. The same data suggested a greater cellular growth in BMSC compared to ASC in both osteogenesis and adipogenesis through the whole differentiation. The data also indicated a greater cell cycle function at the beginning of osteogenesis in BMSC *vs.* ASC (Figure 5 and Table S10). The overall data appear to indicate a greater cell proliferation in BMSC at the early phases of both differentiations compared to ASC. These data are partly supported by the data on the number of cells (Figure S2). In fact, the cell number increased significantly through dd21 in BMSC undergoing osteogenesis but not undergoing adipogenesis; while within the same time frame ASC undergoing adipogenesis had a significant decrease in cell number. Overall the number of cells was significantly greater in BMSC compared to ASC during both differentiations (Figure S2). Our *in vitro* data using a 3D system also support a larger cell proliferation in BMSC compared to ASC during late osteogenesis [17].

For the signaling four functions were identified by IPA and were related to: cell signaling, cell-to-cell signaling and interaction, cellular compromise, and cellular movement (Figure 5). The interpretation of IPA results indicated that during early adipogenesis the BMSC had a greater movement but a lower migration (i.e., immune cell trafficking from IPA) compared to ASC, while the ASC had a greater ability to adhere to other cells and were more activated (i.e., more sensitive to external signals as suggested by cell-to-cell signaling from IPA) (Table S7). This latter feature in ASC *vs.* BMSC appeared to be retained at dd7 with greater hydrolysis of GTP and calcium release. At this same time, data also indicated that ASC appeared to have more synthesis of nitric oxide compared to BMSC (Table S8). Nitric oxide is an important factor in increasing blood flux and angiogenesis [73]. Release of nitric oxide appears to be part of the immunomodulatory mechanism in MSC [74]. During the osteogenic differentiation, data indicated a greater induction of cell movement and immunomodulatory effects (i.e., deletion of T-lymphocytes) in ASC *vs.* BMSC (Tables S10, S11). Overall, our data appear to indicate, as reported above, a greater angiogenic effect and a more pronounced immunomodulatory effect of ASC *vs.* BMSC during both differentiations, but with a relatively greater effect during the adipogenic differentiation. DAVID analysis uncovered a significant enrichment of signal and secretion in DEG more expressed in BMSC *vs.* ASC at dd7 of osteogenesis (file S8, Sheet 5). Moreover, the angiogenesis and cell adhesion was significantly enriched in DEG more expressed in ASC *vs.* BMSC at dd2 of adipogenesis, confirming the conclusion from IPA analysis (file S8, Sheet 1).

The function morphology included a cluster of five functions: cell morphology, cellular assembly and organization, cellular function and maintenance, tissue morphology, and tumor morphology (Figure 5). The interpretation of IPA functions indicated that there was not a greater morphogenesis in one cell type vs. the other during adipogenesis (Tables S7, S8, and S9). Interestingly, the data suggested a greater tumorigeneicity for ASC *vs.* BMSC at dd2 of adipogenesis. During osteogenesis several of the functions related with morphology were significantly enriched, but there were no specific differences uncovered between the two MSC. However, the IPA revealed a greater tissue morphology function for BMSC *vs.* ASC in early osteogenesis (dd2). The DEG between BMSC and ASC in early osteogenesis were overrepresented by genes related to bone loss and increased thickness of connective tissue (Table S10). A direct comparison between ASC and BMSC for tumor proliferation has not been performed, but recent data indicate a specific homing and contribution for tumor growth and proliferation by transplanted MSC [75]. The relationship of the tumor growth function to the MSC is not clear, but it may have contrasting effects: on the one hand MSC inhibit tumor formation by releasing anti-tumor factors, while on the other hand MSC actively increase angiogenesis in tumor masses [76]. In addition, it has been shown that human ASC promote tumor growth in mouse [77]. Our data indicate a potentially greater tumorigenesis effect in ASC during adipogenesis compared to BMSC. Those conclusions however need to be verified by *in vivo* experiments. The analysis with DAVID uncovered a significant enrichment of adherens junction and extracellular region, including collagen-related terms, in DEG that were more expressed in ASC *vs.* BMSC at dd2 of adipogenesis, (file S8, Sheet 1). Other results from DAVID confirmed IPA results.

#### Overall pathway enrichment

Only 4 pathways were found to be significantly enriched by IPA (FDR P-value ≤0.05) in at least one time point comparison among the DEG between BMSC and ASC during adipogenic and/or osteogenic differentiation (Figure S5, details in file S7). In DAVID only the ‘ECM-receptor interaction’ was significantly enriched in DEG more expressed in ASC *vs.* BMSC at dd2 of adipogenesis (file S8, Sheet 1). In IPA only the ‘14-3-3 mediated signaling’ was enriched at dd2 of osteogenic differentiation while ‘keratan sulfate biosynthesis’, ‘LPS/IL-1 mediated inhibition of RXR function’, and ‘sulfur metabolism’ were enriched at dd21 of adipogenic differentiation. Among those only the ‘keratan sulfate biosynthesis’ was obviously more enhanced in ASC vs. BMSC during adipogenesis. The significance of this is not readily apparent; however, the higher amount of keratin sulfate might indicate a lower adherence or a lower migration [78] of ASC during adipogenesis compared to osteogenesis.

### Network between DEG

In order to uncover potential interactions between DEG in each comparison we have run network analysis using IPA, considering all the potential interactions between genes. Results are reported in file S7. Few observations from such analysis can be made: the larger network in osteogenic vs. adipogenic differentiation was observed at dd7 for BMSC and at dd21 for ASC. Interestingly, in both comparisons the networks were almost completely formed by genes more expressed in osteogenic vs. adipogenic, suggesting that the differentiation toward osteogenesis requires a large interaction of genes products compared to adipogenesis.

Networks formed by DEG between differentiations in ASC showed an apparent central role for CEBPA in controlling expression of genes more expressed in adipogenic vs. osteogenic and YWHAZ (Tyrosine 3-monooxygenase/tryptophan 5-monooxygenase activation protein, zeta) in regulating expression of genes more expressed in osteogenic vs. adipogenic. For BMSC the largest network was observed at dd7 with ESR1 (estrogen receptor 1) and a network encompassing YWHAZ, RARA (retinoic acid receptor), NKX2-1 (NK2 homeobox 1 or Thyroid transcription factor 1), CDC42 [cell division cycle 42 (GTP binding protein, 25 kDa)], and ATXN1 (ataxin 1) playing a central role in controlling expression of genes more expressed in osteogenic vs. adipogenic. Interestingly, a central role of IL1B was revealed by networks of DEG between adipogenesis and osteogenesis in both MSC, but in particular in BMSC (file S7).

The proteins coded by DEG between MSC in each differentiation produced small interactive networks with few genes likely playing a central role (file S7).

### Adipogenic and osteogenic markers

The detailed presentation of the results and discussion for finding specific adipogenic and osteogenic markers is reported in file S1 and S10. The results indicate that NAD(P)H dehydrogenase quinone 1 (*NQO1*) is the best overall marker of adipogenesis followed by aquaporin 3 (*AQP3*), stearoyl-CoA desaturase (*SCD*), fatty acid binding protein 3 and 5 (*FABP3* and *FABP5*), and ferritin light polypeptide (*FTL*). For osteogenesis the best markers were hemopexin (*HPX*), collagenase type 3 α1 (*COL3A1*), annexin A8-like 1 (*ANXA8L1*), flotillin 2 (*FLOT2*), and periostin or osteoblast specific factor (*POSTN*).

### Limitations

The experiment and methods used have several limitations that need to be acknowledged. It is generally accepted that the *in vitro* experimental conditions are different from the *in vivo* conditions. For stem cells this is particularly true as they are maintained in an undifferentiated state by the *in vivo* “niche” [79]. In the *in vitro* environment most of the niche environmental conditions are absent. Therefore, the data generated and conclusions need to be verified in an *in vivo* milieu. In addition, the *in vitro* conditions used in the present experiment are effective in inducing osteogenic and adipogenic differentiation [18] however we do not know if those conditions are optimal for those differentiation processes. In support of this, recent data showed that use of different media critically changed the performance of rabbit MSC [80].

The microarray platform used contained ca. 10,000 genes, which is probably less than half of the complete mRNA pool in pig. Thus, all conclusions are inferred using less than half of the complete genome information. In addition, using DAVID the enrichment analysis revealed that the platform is significantly enriched by several terms compared to the whole genome (file S11), indicating that the genes coded by the oligos in the platform are not completely randomly distributed in the whole genome.

The enrichment analysis presents several limitations [35], [81] for a full biological interpretation of the data. Among those, the most critical is the substantial effect of the dimension of the gene list in determining the final enrichment. A consequence of this limitation is the difficulty and inaccuracy in comparing functional analysis between gene lists [35]. Both IPA and DAVID do not provide any buffer/adjustment to address such limitation. Despite those limitations the enrichment analysis is still the most accepted method to uncover the most important “biological effect” contained in the lists of DEG.

Our porcine microarray was annotated using human Entrez gene ID; therefore, DAVID results for the UCSC-TFBS have to be taken with caution since the human promoter region can have different motifs compared to the porcine promoter region.

### Concluding remarks

The data generated by the present study suggest:

The undifferentiated ASC and BMSC have a low expression of genes related to the immune system and hematopoiesis; thus suggesting, as for human, that porcine ASC and BMSC are immune-privileged;

There is a large variation of the ASC and BMSC transcriptome between animals but there are relatively few genes differentially expressed between the two cell types. The functional analysis of DEG between these porcine MSC indicated a greater myogenic capacity of the ASC vs. BMSC but also a greater angiogenesis, migration capacity, bone formation capacity, and neuronal differentiation capacity. In addition there was a greater likelihood of the undifferentiated BMSC for differentiating toward liver and pancreas compared to the undifferentiated ASC. Moreover, those differences appeared to be under the control of few TF, such as BRACH, HSF2, SRY, RFX1, and ARP1;

The direct comparison between differentiations uncovered relatively few genes as crucial in determining the osteogenic or adipogenic fate of MSC, with a larger transcriptomics difference between differentiations of BMSC compared to ASC at the end of the first week of differentiation. Functional analysis allowed identifying cytoskeleton modification and lipid metabolism as the most crucial functions. The functional analysis uncovered a crucial role for the G-proteins and PPAR signaling in directing the cell to differentiate into osteocytes or adipocytes. In addition, the data indicated that MSC undergoing adipogenic differentiation are more angiogenic and, may be more prone to induce tumor formation. This latter observation was more pronounced in ASC compared to BMSC;

The transcriptome of ASC and BMSC during the two differentiations was similar. The transcriptome between the two MSC became virtually equal after the first week of differentiation. The functional analysis of the few DEG indicated that ASC retained in any condition a greater capacity for lipid metabolism and migration than BMSC. Moreover, the ASC were more angiogenic, and probably more tumorigenic, and had less proliferative capacity compared to BMSC under same conditions. Our data also suggested that MSC undergoing differentiation of the tissue of origin (ASC in adipose and BMSC in bone) were more prone to induce inflammation than when undergoing differentiation to the other tissue.

**Figure 6:**
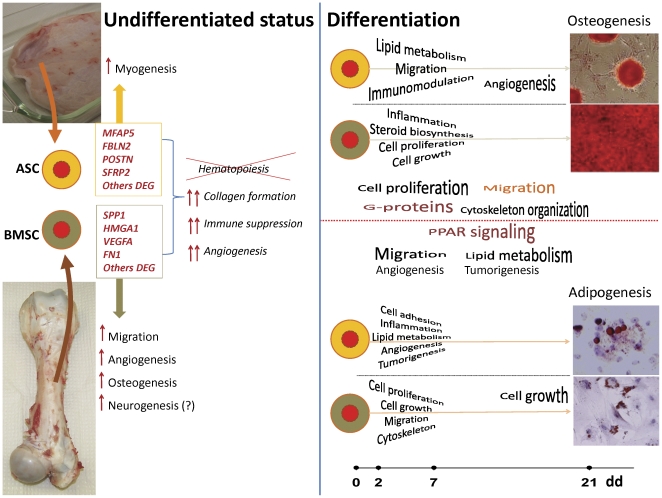
Functional model summarizing the main findings from the present study. Results from the transcriptome analysis of ASC and BMSC induced to differentiate towards the adipogenic and osteogenic lineages suggest: prior differentiation the ASC appear to be more myogenic compared to the BMSC, while these latter appear to be more prone to migrate, to induce angiogenesis, and to differentiate into bone and neurons compared to the ASC. Both MSC have a low expression of several genes related to immune response and hematopoiesis, while they express in large amount genes involved in formation of collagen, immune suppression, and angiogenesis. All those data strongly support an immunomodulatory and angiogenic capacity of MSC with few (but maybe crucial?) differences between BMSC and ASC. During early osteogenic differentiation the data suggest ASC having larger lipid metabolism, migration, and immunomodulatory capacity compared to BMSC, while BMSC have larger induction of inflammation, cell growth, and cell proliferation with a likely larger production of cholesterol. In late osteogenesis ASC appear to have larger angiogenic capacity compared to BMSC. During early adipogenesis the ASC appear to be more prone to induce inflammation, to have higher cell adhesion, tumorigenesis, and angiogenesis compared to BMSC, while BMSC have larger cell proliferation and growth, migration, and cytoskeleton modification compared to ASC. The cell growth appears to be more induced in BMSC *vs.* ASC also at the end of adipogenesis. The direct comparison between differentiations suggest an early larger cell proliferation and later larger cell migration (only in ASC) and cytoskeleton modification in osteogenesis *vs.* adipogenesis with a crucial role of G-proteins. A larger migration and angiogenesis in early differentiation and larger lipid metabolism and tumorigenesis in later differentiation seem to characterize the adipogenic *vs.* osteogenic differentiation with a pivotal role of PPAR signaling.

Finally, we have identified several adipogenic and osteogenic markers, some of which have not been reported previously. The *NQO1* appeared to be the best adipogenic marker followed by *AQP3* and *SCD*. Good osteogenic markers appeared to be *HPX*, *COL3A1*, and *ANXA8L1*;

The conclusions from the present investigation allow proposing a model (Figure 6). This model provides several hypotheses that need to be verified by *in vitro* and/or *in vivo* studies with direct comparisons between the two MSC.

### Prospective

Even though our study confirmed a similar transcriptome (i.e., few DEG in direct comparisons) between BMSC and ASC both at the undifferentiated status and during osteogenic and adipogenic differentiation, the few DEG can have a great impact for the potential therapeutic application of these cells. To understand the potential impact of those few DEG and, overall, to uncover the best stem cell treatment for each specific ailment, the two MSC need to be tested by direct comparison of their bone healing capacity in *in vivo* experiments. We believe that this should be one of the priorities for the research in clinical application of stem cells in the future.

## Materials and Methods

### Ethics Statement

Subcutaneous back fat and bone marrow from femurs were harvested from three castrated Yorkshire crossbred male pigs under a protocol approved for this study by the University of Illinois Institutional Animal Care and Use Committee (IACUC #04296).

### ASC and BMSC isolation, culture, and osteogenic and adipogenic differentiation

ASC and BMSC were isolated, cultured, and induced to differentiate in adipogenic and osteogenic lineages as described previously [18].

### Microarray and qPCR analysis

In a previous study [18] we characterized and directly compared by histological staining and qPCR analysis the *in vitro* differentiation of ASC and BMSC towards the osteogenic and adipogenic lineages. Based on the results obtained we chose the time points for the microarray analysis of this study. At 0, 2, 7, and 21 days of differentiation (dd) cells were trypsinized and total RNA was immediately isolated using the RNeasy® Mini Kit (Qiagen Sciences, Germantown, MD) according to the manufacturer's protocol. Any residual genomic DNA was eliminated using RNase-Free DNase Set® (Qiagen). RNA concentration was measured with a NanoDrop ND-1000 spectrophotometer (NanoDrop Technologies, Wilmington, DE, http://www.nanodrop.com). The purity of RNA (A260/A280) was >1.90.

Total RNA (10 µg) from samples and a reference standard derived from a mixture of porcine tissues (RNA from jejunum, liver, kidney, and mammary tissue in 34%, 32.5%, 32.5%, and 0.7% proportion, respectively) were used to make aminoallyl-labeled cDNA followed by incorporation of Cy3-ester and Cy5-ester (Amersham, Piscataway, NJ) as previously described [82] with slight modifications (see file S1). Each experimental sample was co-hybridized with an equal amount of labeled cDNA of the reference standard.

A 13,309 oligo 70-mers array developed by Operon (Sus scrofa AROS V1.0 with extension) [83] spotted in duplicate on amino silane-coated glass slides was used for transcript profiling. The platform contained oligos for ca. 10,000 unique annotated genes. Annotation was based on similarity searches (September 2007) using sequential Basic Local Alignment Search Tool (BLASTN and TBLASTX against human and mouse UniGene databases and the human genome) as previously described [84]. Details of the microarray are reported in file S1. Slides were scanned for both dye channels with a Scanarray 4000 (GSI-Lumonics, Billerica, MA) dual-laser confocal scanner and images were processed and edited using GenePix 6.0 (Axon Instruments, Concord, ON, http://www.moleculardevices.com). Array quality was assessed using homemade software written in Perl language as previously described [82]. Details of the microarray protocol are reported in file S1. In order to validate the microarray analysis accuracy a direct comparison with data from quantitative RT-PCR (qPCR) for 14 genes previously published using the same samples [18] was performed (file S2).

Microarray data are deposited in the National Center for Biotechnology Information (NCBI) Gene Expression Omnibus (GEO) database (accession GSE25854).

### Statistical Analysis

Microarray spots with median intensity ≥3 standard deviation above the median of the background and GenePix flag >100 were applied as filters to ensure high quality data. Data from a total of 82 microarrays were adjusted for dye and array effect (Loess normalization and array centering), duplicated spot intensities were not averaged and were subsequently used for statistical analysis. A mixed model with repeated measures was then fitted to the normalized log2-transformed adjusted ratios (sample/reference standard) using Proc MIXED (SAS, SAS Inst. Inc., Cary, NC). The model included the fixed effects of time (2, 7, and 21 dd), cell type (ASC and BMSC), differentiation (osteogenic and adipogenic), interactions: time×cell type×differentiation; time×differentiation; time×cell type; cell type×differentiation. Dye and expression at dd0 was a covariate and pig (n = 3) was considered as random effect. P-values were adjusted for the number of genes tested using Benjamini and Hochberg's false discovery rate (FDR) [85] to account for multiple comparisons. Differences in relative expression were considered significant at an FDR-adjusted P≤0.05 for time×cell type×differentiation. Post-hoc P<0.001 was considered significant between pairwise comparisons. For the comparison between the two cell types before differentiation (dd0) a second statistical analysis was run excluding dd0 as covariate and using a FDR ≤0.05 cut-off for the overall MSC effect (i.e., considering all time points) and with P<0.001 between the two MSC at dd0. The difference in expression of genes is reported as fold change (2-fold = 100% change). All correlation analyses were performed using Proc Corr of SAS (SAS Institute, Cary, NC). The contrasts between factor levels and P-values are reported in file S2.

### Data mining

The data mining analysis of the transcriptome was performed in three separate result sub-sets: 1) at dd0 or prior to differentiation, which included differentially expressed genes (DEG) between the two MSC, genes highly expressed in MSC, and genes more expressed in MSC compared to fully differentiated tissues (i.e., microarray reference RNA, see above); 2) on DEG between differentiations in each MSC for each time point; and 3) on DEG between MSC within each differentiation for each time point.

The functional analysis was performed using Ingenuity Pathway Analysis (**IPA**; Ingenuity® Systems, Mountain View, CA) and Database for Annotation Visualization and Integrated Discovery (DAVID) [86]. The use of multiple tools is highly recommended for functional analysis of microarray data [35].

For the IPA the entire microarray data set with associated statistical FDR P-values were considered using GenBank or HUGO ids. The annotated oligos in the pig array were used as the reference set for functional and canonical pathway analysis. In IPA, functions and pathways were considered significantly enriched with a threshold of FDR P-value ≤0.05. The interpretation of IPA data is as reported previously [87] and described in file S1.

The same gene lists as for IPA and the gene list of up- and down-regulated DEG were analyzed in DAVID [86] using Entrez Gene ID, and the entire annotated pig array was used as background. Analysis was run using default functional categories tools of DAVID plus UP-Tissue and UCSC-TFBS (transcription factor binding site). Results that had an EASE score ≤0.1 were considered and all reported following suggestions from Huang da *et al.* [86]. Results with an EASE score ≤0.1 were discussed only when not terms were enriched with a Benjamini-Hochberg (B–H) multiple comparison ≤0.05, otherwise a B–H ≤0.05 was used as cut-off. For some analysis the Functional Annotation Clustering tool in DAVID [86] was used with the highest classification stringency in order to group redundant terms and determine their overall enrichment score [88].

## Supporting Information

Figure S1 **Pearson correlation between human ASC and BMSC.** Pearson correlation is 0.975 with a p-value <0.0001, calculated with Proc SAS as reported in Materials and Methods of the main body of the paper. The correlation between each human individual ranged from 0.954 to 0.963.
(TIF)

Figure S2 **Number of cells before starting differentiation and during the adipogenic and osteogenic differentiation in porcine ASC and BMSC.** The data were normalized by log2 transformation prior analysis. The model included time, cell type, differentiation and interactions: time×cell type×differentiation; time×differentiation; time×cell type; cell type×differentiation. Pig (n = 3) was included as random variable. A post-hoc correction using Tukey's was applied. The time×cell type×differentiation was significant at p = 0.02 and all the main effects and interactions were significant with exception of time. Different letters denote p<0.05; * and # denote significant (p<0.05) difference relative to dd0 in ASC during adipogenic differentiation and BMSC during osteogenic differentiation, respectively.
(TIF)

Figure S3 **Significant enriched pathways between adipogenic and osteogenic differentiation in ASC and BMSC.** Pathways from Ingenuity Pathway Analysis significantly enriched (B–H-FDR ≤0.05) in at least one comparison among DEG between adipogenic and osteogenic differentiation in ASC (black line) and BMSC (red line). The lines and markers denote the significance of enrichment in –log B–H-FDR (e.g., 0.05 = 1.33; 0.01 = 2.0) and cross symbols denote ratio of DEG/genes composing the pathway (black ASC and red BMSC). The blue line denotes a B–H FDR of 0.05 (-log B–H FDR of 1.33).
(TIF)

Figure S4 **Quantity of RNA per cell (ng) before starting differentiation and during the adipogenic and osteogenic differentiation in porcine ASC and BMSC.** The model included time, cell type, differentiation and interactions: time×cell type×differentiation; time×differentiation; time×cell type; cell type×differentiation. Pig (n = 3) was included as random variable. A post-hoc correction using Tukey's was applied. The time×cell type×differentiation and overall differentiation were not significant (p = 0.81 and p = 0.24, respectively); all the other main effects and interactions were significant (p<0.05). * and # denote significant (p<0.05) difference relative to dd0.
(TIF)

Figure S5 **Significant enriched pathways between ASC and BMSC during adipogenic and osteogenic differentiation.** Pathways from Ingenuity Pathway Analysis significantly enriched (B–H-FDR ≤0.05) in at least one comparison among DEG between ASC and BMSC during adipogenic (black line) and osteogenic (red line) differentiation. The lines and markers denote the significance of enrichment in –log B–H-FDR (e.g., 0.05 = 1.33; 0.01 = 2.0) and cross symbols denote ratio of DEG/genes composing the pathway (black adipogenic and red osteogenic). The blue line denotes a B–H FDR of 0.05 (-log B–H FDR of 1.33).
(TIF)

File S1 **Supplementary Materials and Methods and Results and Discussion.**
(DOCX)

File S2 **Entire microarray results during differentiations plus qPCR and microarray comparison.** The excel file contains 4 sheets: “***Cell type comparison***” microarray results of the direct comparison between ASC and BMSC during both adipogenic and osteogenic differentiation; “***Differentiation comparison***” microarray results of the direct comparison between adipogenic and osteogenic differentiation in ASC and BMSC; “***qPCR vs Array adipogenic diff***” comparison between qPCR and microarray analysis for the adipogenic differentiation; “***qPCR vs Array osteogenic diff***” comparison between qPCR and microarray analysis for the osteogenic differentiation.
(XLSX)

File S3 **Results of functional analysis of genes highly expressed in both MSC prior differentiation.** The excel sheet presents 6 sheets: “***Sheet 1 -Transcripts >1,000 RFU***” list of genes expressed with a Relative Fluorescence Units >1,000 (≥ to the signal of GAPDH) in our microarray; “***Sheet 2 - Funct chart >1000 RFU***” functional chart analysis results from DAVID of genes expressed with a Relative Fluorescence Units >1,000; “***Sheet 3-Funct cluster >1000 RFU***” functional cluster analysis results from DAVID of genes expressed with a Relative Fluorescence Units >1,000; “***Sheet 4 Funct chart >10000RFU***” functional chart analysis results from DAVID of genes expressed with a Relative Fluorescence Units >10,000; “***Sheet 5 Funct cluster >10000RFU***” functional cluster analysis results from DAVID of genes expressed with a Relative Fluorescence Units >10,000; “***Sheet 6 IPA Pathway >10000RFU***” depiction of the “Intrinsic Prothrombin Activation Pathway” from Ingenuity Pathway Analysis.
(XLSX)

File S4 **Results of functional analysis of genes more expressed in both MSC prior to differentiation compared to fully differentiated tissues.** The excel file presents 11 sheets: “***Sheet 1 MSC vs references***” list of genes more expressed in MSC vs. fully differentiated tissues; “***Sheet 2 Funct chart DEG>2fold***” functional chart analysis results from DAVID of genes expressed >2-fold in MSC vs. fully differentiated tissues; “***Sheet 3 Funct cluter DEG>2fold***” functional cluster analysis results from DAVID of genes expressed >2-fold in MSC vs. fully differentiated tissues; “***Sheet 4 IPA functions DEG>2fold***” functional analysis results from Ingenuity Pathway Analysis of genes expressed >2-fold in MSC vs. fully differentiated tissues; “***Sheet 5 IPA effect funct >2fold***” effect on function analysis results from Ingenuity Pathway Analysis of genes expressed >2-fold in MSC vs. fully differentiated tissues; “***Sheet 6 IPA pathways DEG>2fold***” list and depiction of pathways significantly enriched (Benjamini-Hochberg P≤0.05) in Ingenuity Pathway Analysis of genes expressed >2-fold in MSC vs. fully differentiated tissues; “***Sheet 7 Funct chart DEG<0.5FD***” functional chart analysis results from DAVID of genes expressed ≤0.5-fold in MSC vs. fully differentiated tissues; “***Sheet8 Funct cluster DEG<0.5FD***” functional cluster analysis results from DAVID of genes expressed ≤0.5-fold in MSC vs. fully differentiated tissues; “***Sheet 9 IPA functions DEG<0.5FD***” functional analysis results from Ingenuity Pathway Analysis of genes expressed ≤0.5-fold in MSC vs. fully differentiated tissues; “***Sheet 10 IPA eff func DEG<0.5FD***” effect on function analysis results from Ingenuity Pathway Analysis of genes expressed ≤0.5-fold in MSC vs. fully differentiated tissues; “***Sheet 11 IPA pathways DEG<0.5FD***” list of pathways significantly enriched (Benjamini-Hochberg P≤0.05) in Ingenuity Pathway Analysis of genes expressed ≤0.5-fold in MSC vs. fully differentiated tissues.
(XLSX)

File S5 **Results of functional analysis of genes differentially expressed in BMSC and ASC prior differentiations.** The excel file contains 9 sheets: “***Sheet 1 ASC vs BMSC FDR0.05 p<0.001***” complete list of DEG between BMSC and ASC prior differentiation; “***Sheet 2 Funct chart DEG>ASC***” functional chart analysis results from DAVID of genes more expressed in ASC *vs.* BMSC; “***Sheet 3 Funct cluster DEG>ASC***” functional cluster analysis results from DAVID of genes more expressed in ASC *vs.* BMSC; “***Sheet 4 IPA Func DEG>ASC vs BMSC***” functional analysis results from Ingenuity Pathway Analysis of genes more expressed in ASC *vs.* BMSC; “***Sheet 5 Effect funct DEG>ASC***” effect on function analysis results from Ingenuity Pathway Analysis of genes more expressed in ASC *vs.* BMSC; “***Sheet 6 Funct chart DEG>BMSC***” functional chart analysis results from DAVID of genes more expressed in BMSC *vs.* ASC; “***Sheet 7 Funct cluster DEG>ASC***” functional cluster analysis results from DAVID of genes more expressed in BMSC *vs.* ASC; “***Sheet 8 IPA Func DEG>ASC vs BMSC***” functional analysis results from Ingenuity Pathway Analysis of genes more expressed in BMSC *vs.* ASC; “***Sheet 9 Effect funct DEG>ASC***” effect on function analysis results from Ingenuity Pathway Analysis of genes more expressed in BMSC *vs.* ASC.
(XLSX)

File S6 **DAVID functional analysis results for genes differentially expressed between adipogenesis and osteogenesis in ASC and BMSC during differentiation.** The excel file contains 6 sheets: “***Adipo vs Osteo ASC dd2***” functional analysis results for both DEG more expressed in adipogenesis and more expressed in osteogenesis for ASC at dd2; “***Adipo vs Osteo ASC dd7***” functional analysis results for both DEG more expressed in adipogenesis and more expressed in osteogenesis for ASC at dd7; “***Adipo vs Osteo ASC dd21***” functional analysis results for both DEG more expressed in adipogenesis and more expressed in osteogenesis for ASC at dd21; “***Adipo vs Osteo BMSC dd2***” functional analysis results for both DEG more expressed in adipogenesis and more expressed in osteogenesis for ASC at dd2; “***Adipo vs Osteo BMSC dd7***” functional analysis results for both DEG more expressed in adipogenesis and more expressed in osteogenesis for BMSC at dd7; “***Adipo vs Osteo BMSC dd21***” functional analysis results for both DEG more expressed in adipogenesis and more expressed in osteogenesis for BMSC at dd21.
(XLSX)

File S7 **Results of significantly enriched pathway analysis from Ingenuity Pathway Analysis of DEG between adipogenesis and osteogenesis and DEG between BMSC and ASC during differentiation.** The file contains the complete list of enriched pathways (P-value ≤0.05) and figures of the enriched pathways for each comparison.
(DOCX)

File S8 **DAVID functional analysis results for genes differentially expressed between ASC and BMSC during adipogenic and osteogenic differentiation.** The excel file contains 6 sheets: “***Sheet 1 Adipo BMSC vs ASC dd2***” functional analysis results for both DEG more expressed in BMSC and more expressed in ASC during adipogenesis at dd2; “***Sheet 2 Adipo BMSC vs ASC dd7***” functional analysis results for both DEG more expressed in BMSC and more expressed in ASC during adipogenesis at dd7; “***Sheet 3 Adipo BMSC vs ASC dd21***” functional analysis results for both DEG more expressed in BMSC and more expressed in ASC during adipogenesis at dd21; “***Sheet 4 Osteo BMSC vs ASC dd2***” functional analysis results for both DEG more expressed in BMSC and more expressed in ASC during osteogenesis at dd2; “***Sheet 5 Osteo BMSC vs ASC dd7***” functional analysis results for both DEG more expressed in BMSC and more expressed in ASC during osteogenesis at dd7; “***Sheet 6 Osteo BMSC vs ASC dd21***” functional analysis results for both DEG more expressed in BMSC and more expressed in ASC during osteogenesis at dd21.
(XLSX)

File S9 **Functional analysis of DEG between BMSC and ASC during the whole differentiation considering an overall tissue effect with FDR ≤0.05.** The excel file contains 14 sheets: “***Sheet 1 DEG ASC vs BMSC FDR0.05***” complete list of DEG; “***Sheet 2 Funct chart all FDR0.05***” functional chart analysis results from DAVID of DEG between ASC and BMSC; “***Sheet 3 Funct Clust all FDR0.05***” functional cluster analysis results from DAVID of DEG between ASC and BMSC; “***Sheet 4 IPA func all FDR0.05***” functional analysis results from Ingenuity Pathway Analysis of DEG between ASC and BMSC; “***Sheet 5 Effect func all FDR0.05***” effect on function analysis of functions significantly enriched in Ingenuity Pathway Analysis of DEG between ASC and BMSC; “***Sheet 6 IPA path all FDR0.05***” pathways analysis in Ingenuity Pathway Analysis of DEG between ASC and BMSC; “***Sheet 7 Funct chart DEG>ASC***” functional chart analysis results from DAVID of DEG more expressed in ASC *vs.* BMSC; “***Sheet 8 Funct cluster DEG>ASC***” functional cluster analysis results from DAVID of DEG more expressed in ASC *vs.* BMSC; “***Sheet 9 IPA Func DEG>ASC vs BMSC***” functional analysis results from Ingenuity Pathway Analysis of DEG more expressed in ASC *vs.* BMSC; “***Sheet 10 Effect funct DEG>ASC***” effect on function analysis of functions significantly enriched in Ingenuity Pathway Analysis of DEG more expressed in ASC *vs.* BMSC; “***Sheet 11 IPA Path DEG>ASC vs BMSC***” pathways analysis results from Ingenuity Pathway Analysis of DEG more expressed in ASC *vs.* BMSC; “***Sheet 12 Funct chart DEG>BMSC***” functional chart analysis results from DAVID of DEG more expressed in BMSC *vs.* ASC; “***Sheet 13 Funct cluster DEG>BMSC***” functional cluster analysis results from DAVID of DEG more expressed in BMSC *vs.* ASC; “***Sheet 14 IPA Path DEG>BMSCvASC***” functional analysis results from Ingenuity Pathway Analysis of DEG more expressed in BMSC *vs.* ASC.
(XLSX)

File S10 **Complete list of markers for osteogenic and adipogenic differentiation with relative functional analysis.** The excel file contains 15 sheets: “***Sheet1 Markers***” complete list of markers; “***Sheet2 David adipog markers***” functional analysis of overall adipogenic markers; “***Sheet3 David osteog markers***” functional analysis of overall osteogenic markers; “***Sheet4 David adipog markers ASC***” functional analysis of adipogenic markers in ASC; “***Sheet5 David osteog markers ASC***” functional analysis of osteogenic markers in ASC; “***Sheet6 David adipog marker BMSC***” functional analysis of adipogenic markers in BMSC; “***Sheet7 David osteog marker BMSC***” functional analysis of osteogenic markers in BMSC; “***Sheet8 David adipog markers 2dd***” functional analysis of adipogenic markers at dd2; “***Sheet9 David adipog markers 7dd***” functional analysis of adipogenic markers at dd7; “***Sheet10 David adipog markers 21dd***” functional analysis of adipogenic markers at dd21; “***Sheet11 David adipog mark 7–21dd***” functional analysis of adipogenic markers between 7 and 21 day of differentiation; “***Sheet12 David osteog marker 2dd***” functional analysis of osteogenic markers at dd2; “***Sheet13 David osteog marker 7dd***” functional analysis of osteogenic markers at dd7; “***Sheet14 David osteog mark 21dd***” functional analysis of osteogenic markers at dd21; “***Sheet15 David osteog mark 7–21dd***” functional analysis of osteogenic markers between 7 and 21 day of differentiation.
(XLSX)

File S11 **Functional chart analysis from DAVID of the entire annotated microarray used in the present experiment (excel file with only 1 sheet).**
(XLSX)

Table S1 **Pearson correlation between ASC and BMSC transcriptome.** Reported are the results from SAS analysis of overall Pearson correlation between ASC and BMSC transcriptome overall, before differentiation (dd0) for all the pigs and for each single pig; between differentiations in the same cell type (ASC or BMSC) overall and for each single pig; and overall correlation between ASC and BMSC during adipogenic and osteogenic differentiation. All correlations were significant at p<0.0001.
(DOCX)

Table S2 **Functional analysis results by IPA of adipogenic and osteogenic differentiation of ASC at dd2.** Tabulated results from Ingenuity Pathway Analysis® (IPA) effect on function analysis of DEG between adipogenic and osteogenic differentiation of ASC at dd2. Reported are the functions sorted by decrease in significance. The category denotes the main functional category assigned by IPA. The function annotation is derived by the “effect on function” in IPA. In parenthesis are reported the number of DEG for each specific function and the arrows denote the overall effect on the function inferred by the gene annotation using IPA (⇑⇑ = highly activated in adipogenic vs. osteogenic differentiation; ⇑ = activated in adipogenic vs. osteogenic differentiation; ↑ = tends to be activated in adipogenic vs. osteogenic differentiation; ⇓⇓ = highly activated in osteogenic vs. adipogenic differentiation; ⇓ = activated in osteogenic vs. adipogenic differentiation; ↓ = tends to be activated in osteogenic vs. adipogenic differentiation) following the criteria reported in Materials and Methods in file S1. In yellow shade all functions enriched with a B–H FDR≤0.05.
(DOCX)

Table S3 **Functional analysis results by IPA of adipogenic and osteogenic differentiation of ASC at dd21.** Tabulated results from Ingenuity Pathway Analysis® (IPA) effect on function analysis of DEG between adipogenic and osteogenic differentiation of ASC at dd21. Reported are the functions sorted by decrease in significance. The category denotes the main functional category assigned by IPA. The function annotation is derived by the “effect on function” in IPA. In parenthesis are reported the number of DEG for each specific function and the arrows denote the overall effect on the function inferred by the gene annotation using IPA (⇑⇑ = highly activated in adipogenic vs. osteogenic differentiation; ⇑ = activated in adipogenic vs. osteogenic differentiation; ↑ = tends to be activated in adipogenic vs. osteogenic differentiation; ⇓⇓ = highly activated in osteogenic vs. adipogenic differentiation; ⇓ = activated in osteogenic vs. adipogenic differentiation; ↓ = tends to be activated in osteogenic vs. adipogenic differentiation.) following the criteria reported in Materials and Methods in file S1.
(DOCX)

Table S4 **Function analysis results by IPA of adipogenic and osteogenic differentiation of BMSC at dd21.** Tabulated results from Ingenuity Pathway Analysis® (IPA) effect on function analysis of DEG between adipogenic and osteogenic differentiation of BMSC at dd21. Reported are the functions sorted by decrease in significance. The category denotes the main functional category assigned by IPA. The function annotation is derived by the “effect on function” in IPA. In parenthesis are reported the number of DEG for each specific function and the arrows denote the overall effect on the function inferred by the gene annotation using IPA (⇑⇑ = highly activated in adipogenic vs. osteogenic differentiation; ⇑ = activated in adipogenic vs. osteogenic differentiation; ↑ = tends to be activated in adipogenic vs. osteogenic differentiation; ⇓⇓ = highly activated in osteogenic vs. adipogenic differentiation; ⇓ = activated in osteogenic vs. adipogenic differentiation; ↓ = tends to be activated in osteogenic vs. adipogenic differentiation) following the criteria reported in Materials and Methods in file S1.
(DOCX)

Table S5 **Pearson correlation between ASC and BMSC transcriptome in each pig and for each time point during adipogenic and osteogenic differentiation.** All correlations were significant at p<0.0001.
(DOCX)

Table S6 **Pearson correlation between pigs (12, 22, and 40) transcriptome in each cell type (ASC or BMSC) during adipogenic and osteogenic differentiation.** All correlations were significant at p<0.0001.
(DOCX)

Table S7 **Function analysis results by IPA of BMSC and ASC during adipogenic differentiation at dd2.** Tabulated results from Ingenuity Pathway Analysis® (IPA) effect on function analysis of DEG between BMSC and ASC during adipogenic differentiation at dd2. Reported are the functions sorted by decrease in significance. The category denotes the main functional category assigned by IPA. The function annotation is derived by the “effect on function” in IPA. In parenthesis are reported the number of DEG for each specific function and the arrows denote the overall effect on the function inferred by the gene annotation using IPA (⇑⇑ = highly activated in BMSC vs. ASC; ⇑ = activated in BMSC vs. ASC; ↑ = tends to be activated in BMSC vs. ASC; ⇓⇓ = highly activated in ASC vs. BMSC; ⇓ = activated in ASC vs. BMSC; ↓ = tends to be activated in ASC vs. BMSC) following the criteria reported in Materials and Methods in file S1. Effect on functions with <2 genes were discarded.
(DOCX)

Table S8 **Function analysis results by IPA of BMSC and ASC during adipogenic differentiation at dd7.** Tabulated results from Ingenuity Pathway Analysis® (IPA) effect on function analysis of DEG between BMSC and ASC during adipogenic differentiation at dd7. Reported are the functions sorted by decrease in significance. The category denotes the main functional category assigned by IPA. The function annotation is derived by the “effect on function” in IPA. In parenthesis are reported the number of DEG for each specific function and the arrows denote the overall effect on the function inferred by the gene annotation using IPA (⇑⇑ = highly activated in BMSC vs. ASC; ⇑ = activated in BMSC vs. ASC; ↑ = tends to be activated in BMSC vs. ASC; ⇓⇓ = highly activated in ASC vs. BMSC; ⇓ = activated in ASC vs. BMSC; ↓ = tends to be activated in ASC vs. BMSC) following the criteria reported in Materials and Methods in file S1. Effect on functions with <2 genes were discarded.
(DOCX)

Table S9 **Functional analysis results by IPA of BMSC and ASC during adipogenic differentiation at dd21.** Tabulated results from Ingenuity Pathway Analysis® (IPA) effect on function analysis of DEG between BMSC and ASC during adipogenic differentiation at dd21. Reported are the functions sorted by decrease in significance. The category denotes the main functional category assigned by IPA. The function annotation is derived by the “effect on function” in IPA. In parenthesis are reported the number of DEG for each specific function and the arrows denote the overall effect on the function inferred by the gene annotation using IPA (⇑⇑ = highly activated in BMSC vs. ASC; ⇑ = activated in BMSC vs. ASC; ↑ = tends to be activated in BMSC vs. ASC; ⇓⇓ = highly activated in ASC vs. BMSC; ⇓ = activated in ASC vs. BMSC; ↓ = tends to be activated in ASC vs. BMSC) following the criteria reported in Materials and Methods in file S1. Effect on functions with <2 genes were discarded.
(DOCX)

Table S10 **Functional analysis results by IPA of BMSC and ASC during osteogenic differentiation at dd2.** Tabulated results from Ingenuity Pathway Analysis® (IPA) effect on function analysis of DEG between BMSC and ASC during osteogenic differentiation at dd2. Reported are the functions sorted by decrease in significance. The category denotes the main functional category assigned by IPA. The function annotation is derived by the “effect on function” in IPA. In parenthesis are reported the number of DEG for each specific function and the arrows denote the overall effect on the function inferred by the gene annotation using IPA (⇑⇑ = highly activated in BMSC vs. ASC; ⇑ = activated in BMSC vs. ASC; ↑ = tends to be activated in BMSC vs. ASC; ⇓⇓ = highly activated in ASC vs. BMSC; ⇓ = activated in ASC vs. BMSC; ↓ = tends to be activated in ASC vs. BMSC) following the criteria reported in Materials and Methods in file S1. Effect on functions with <2 genes were discarded.
(DOCX)

Table S11 **Functional analysis results by IPA of BMSC and ASC during osteogenic differentiation at dd21.** Reported are the functions sorted by decrease in significance. The function annotation is derived by the “effect on function” in IPA. In parenthesis are reported the number of DEG for each specific function and the arrows denote the overall effect on the function inferred by the gene annotation using IPA (⇑⇑ = highly activated in BMSC vs. ASC; ⇑ = activated in BMSC vs. ASC; ↑ = tends to be activated in BMSC vs. ASC; ⇓⇓ = highly activated in ASC vs. BMSC; ⇓ = activated in ASC vs. BMSC; ↓ = tends to be activated in ASC vs. BMSC) following the criteria reported in Materials and Methods in file S1. Effect on functions with <2 genes were discarded.
(DOCX)

Video S1 **ASC Adipogenesis 1.** The video is a time lapse taken for 12 h after addition of adipogenic medium in ASC (replicate 1) cultivated in 24 well plate (2 frames/s; pictures were taken every 20 min).
(FLV)

Video S2 **ASC Adipogenesis 2.** The video is a time lapse taken for 12 h after addition of adipogenic medium in ASC (replicate 2) cultivated in 24 well plate (2 frames/s; pictures were taken every 20 min).
(FLV)

Video S3 **ASC Osteogenesis 1.** The video is a time lapse taken for 12 h after addition of osteogenic medium in ASC (replicate 1) cultivated in 24 well plate (2 frames/s; pictures were taken every 20 min).
(FLV)

Video S4 **ASC Osteogenesis 2.** The video is a time lapse taken for 12 h after addition of osteogenic medium in ASC (replicate 2) cultivated in 24 well plate (2 frames/s; pictures were taken every 20 min).
(FLV)
