# Supplementary figures and images for: Transcriptomics Comparison between Porcine Adipose and Bone Marrow Mesenchymal Stem Cells during In Vitro Osteogenic and Adipogenic Differentiation
Source: PLoS One. 2012 Mar 7;7(3):e32481. doi: 10.1371/journal.pone.0032481 (PMC3296722; doi:10.1371/journal.pone.0032481)

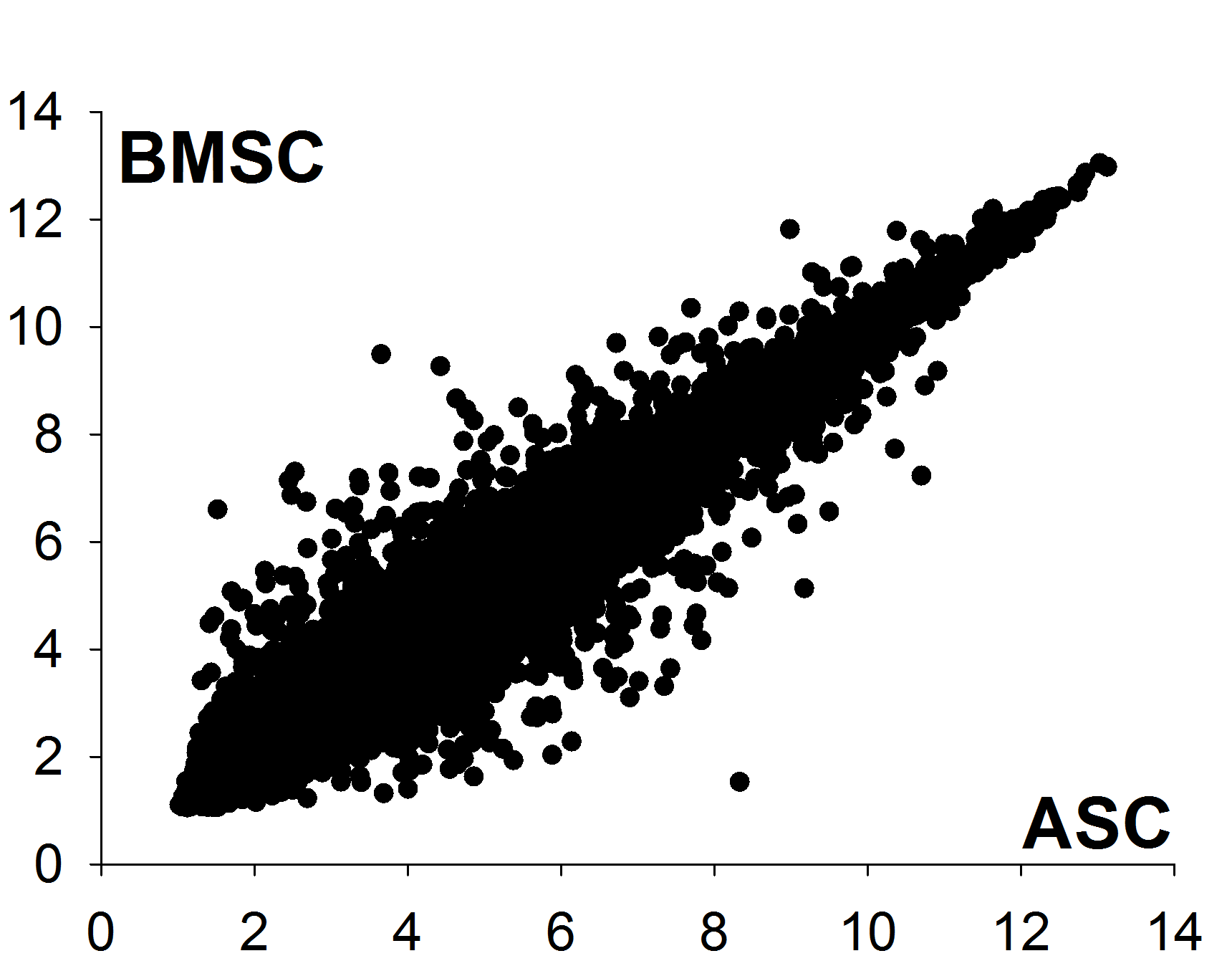

Supplement: Figure S1 — Pearson correlation between human ASC and BMSC. Pearson correlation is 0.975 with a p-value <0.0001, calculated with Proc SAS as reported in Materials and Methods of the main body of the paper. The correlation between each human individual ranged from 0.954 to 0.963. (TIF) [file pone.0032481.s001.tif]

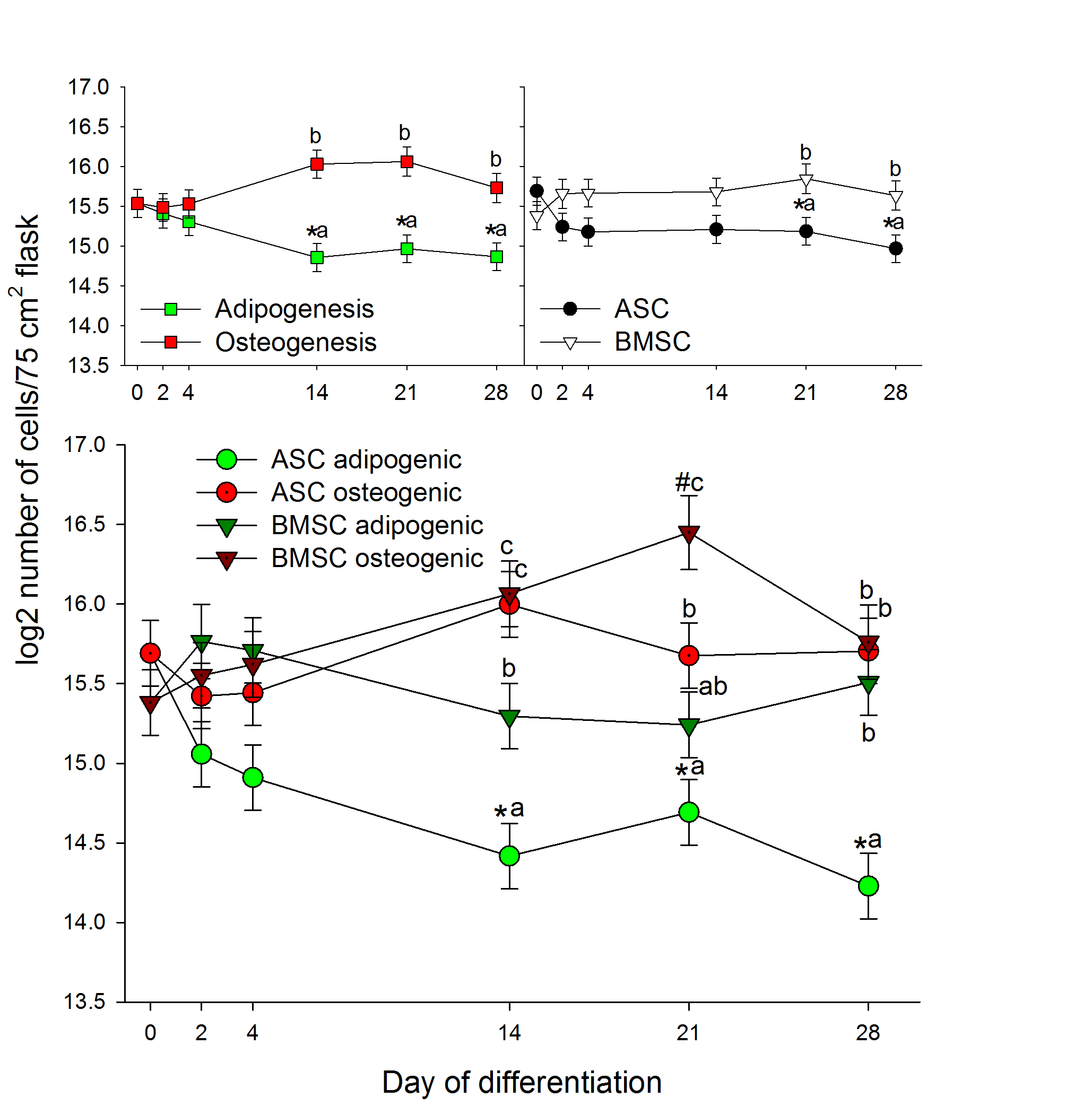

Supplement: Figure S2 — Number of cells before starting differentiation and during the adipogenic and osteogenic differentiation in porcine ASC and BMSC. The data were normalized by log2 transformation prior analysis. The model included time, cell type, differentiation and interactions: time×cell type×differentiation; time×differentiation; time×cell type; cell type×differentiation. Pig (n = 3) was included as random variable. A post-hoc correction using Tukey's was applied. The time×cell type×differentiation was significant at p = 0.02 and all the main effects and interactions were significant with exception of time. Different letters denote p<0.05; * and # denote significant (p<0.05) difference relative to dd0 in ASC during adipogenic differentiation and BMSC during osteogenic differentiation, respectively. (TIF) [file pone.0032481.s002.tif]

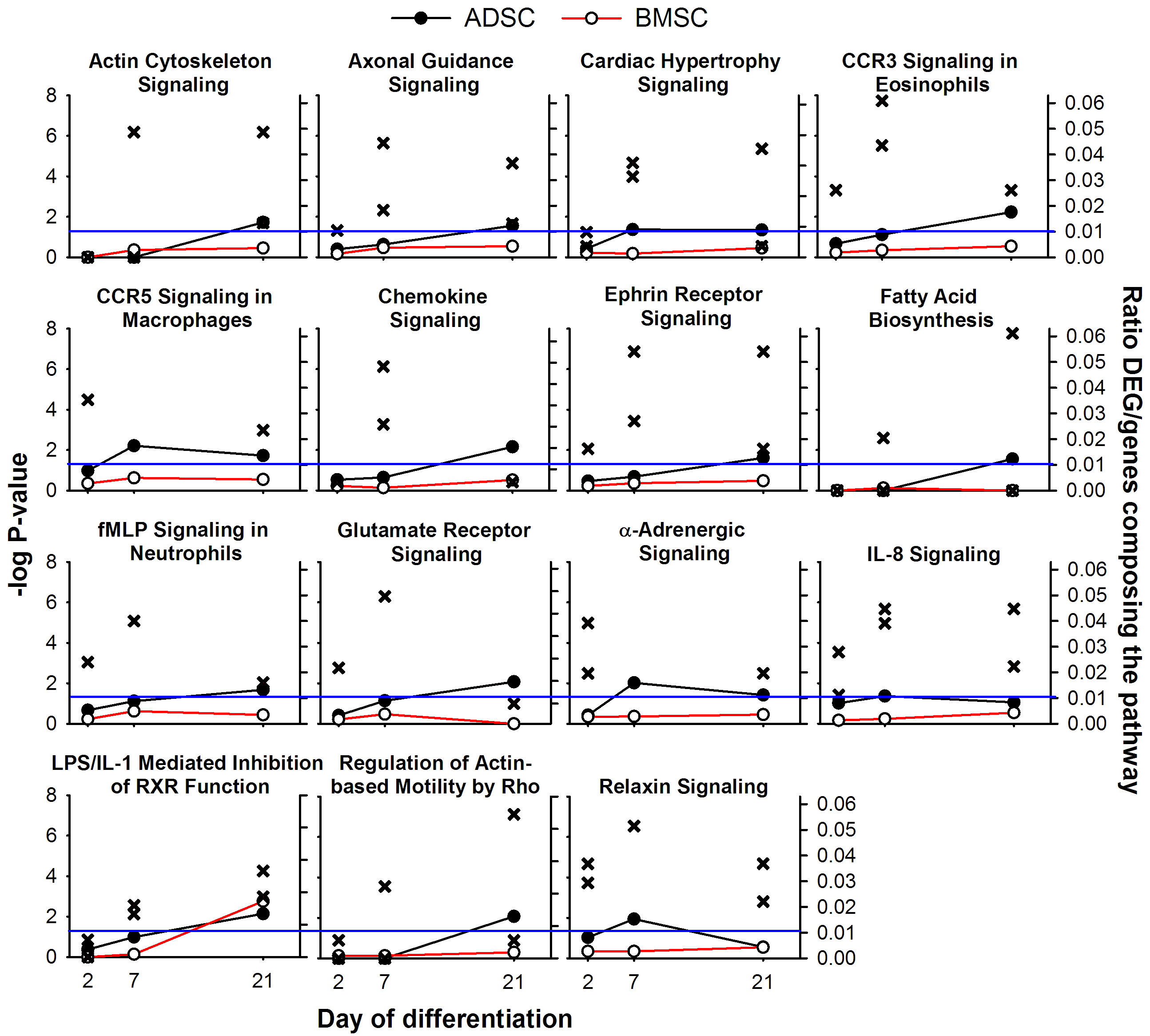

Supplement: Figure S3 — Significant enriched pathways between adipogenic and osteogenic differentiation in ASC and BMSC. Pathways from Ingenuity Pathway Analysis significantly enriched (B–H-FDR ≤0.05) in at least one comparison among DEG between adipogenic and osteogenic differentiation in ASC (black line) and BMSC (red line). The lines and markers denote the significance of enrichment in –log B–H-FDR (e.g., 0.05 = 1.33; 0.01 = 2.0) and cross symbols denote ratio of DEG/genes composing the pathway (black ASC and red BMSC). The blue line denotes a B–H FDR of 0.05 (-log B–H FDR of 1.33). (TIF) [file pone.0032481.s003.tif]

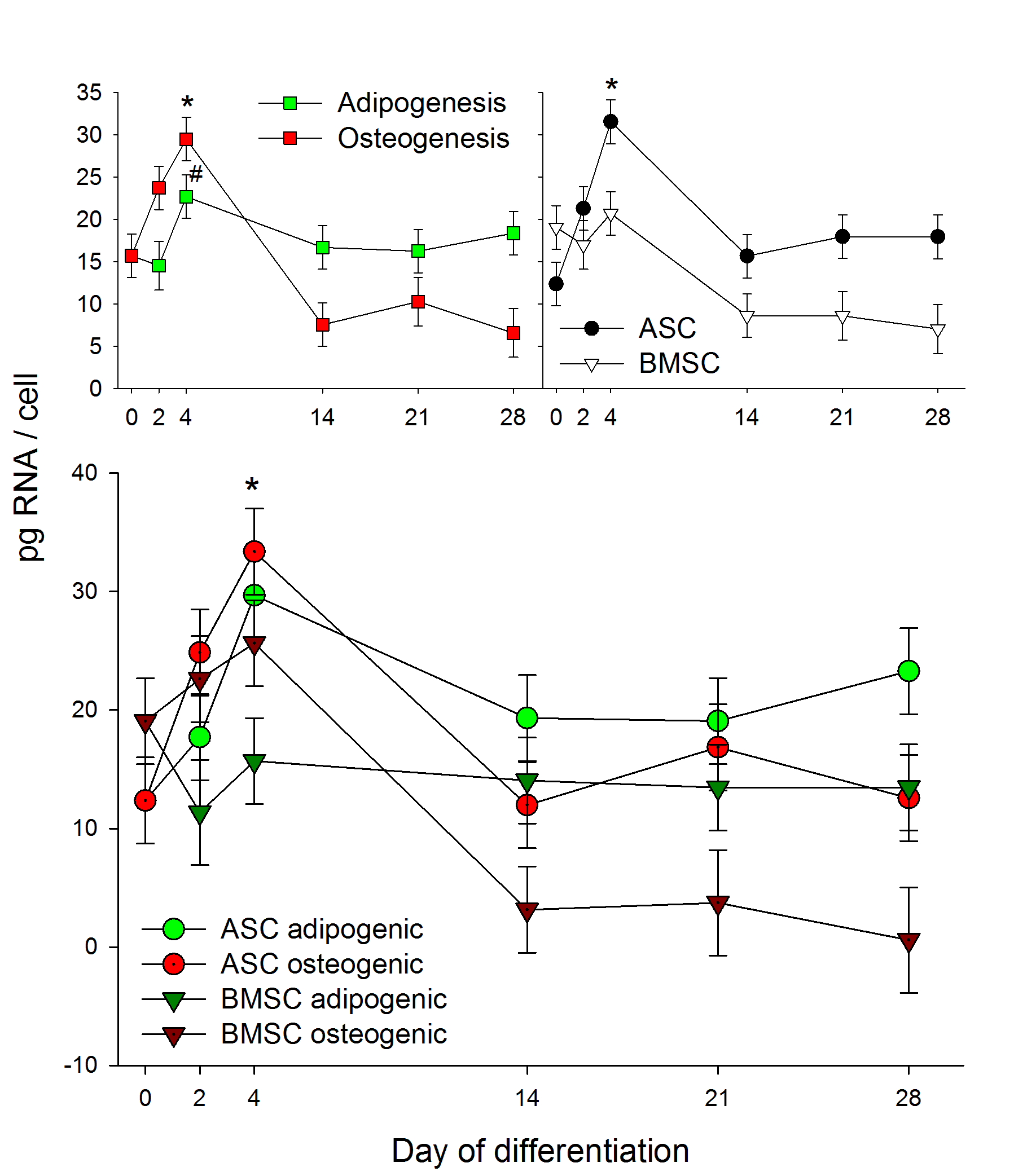

Supplement: Figure S4 — Quantity of RNA per cell (ng) before starting differentiation and during the adipogenic and osteogenic differentiation in porcine ASC and BMSC. The model included time, cell type, differentiation and interactions: time×cell type×differentiation; time×differentiation; time×cell type; cell type×differentiation. Pig (n = 3) was included as random variable. A post-hoc correction using Tukey's was applied. The time×cell type×differentiation and overall differentiation were not significant (p = 0.81 and p = 0.24, respectively); all the other main effects and interactions were significant (p<0.05). * and # denote significant (p<0.05) difference relative to dd0. (TIF) [file pone.0032481.s004.tif]

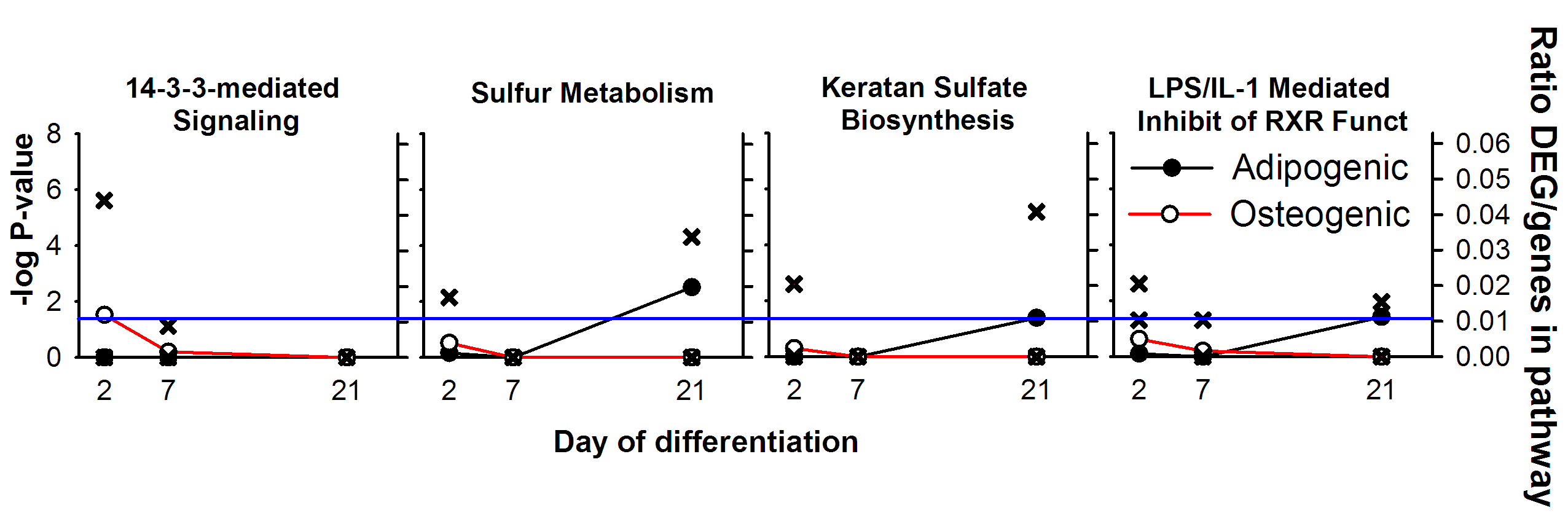

Supplement: Figure S5 — Significant enriched pathways between ASC and BMSC during adipogenic and osteogenic differentiation. Pathways from Ingenuity Pathway Analysis significantly enriched (B–H-FDR ≤0.05) in at least one comparison among DEG between ASC and BMSC during adipogenic (black line) and osteogenic (red line) differentiation. The lines and markers denote the significance of enrichment in –log B–H-FDR (e.g., 0.05 = 1.33; 0.01 = 2.0) and cross symbols denote ratio of DEG/genes composing the pathway (black adipogenic and red osteogenic). The blue line denotes a B–H FDR of 0.05 (-log B–H FDR of 1.33). (TIF) [file pone.0032481.s005.tif]
