# Supplementary material for: Transcriptomics Comparison between Porcine Adipose and Bone Marrow Mesenchymal Stem Cells during In Vitro Osteogenic and Adipogenic Differentiation
Source: PLoS One. 2012 Mar 7;7(3):e32481. doi: 10.1371/journal.pone.0032481 (PMC3296722; doi:10.1371/journal.pone.0032481)
Supplement: File S1 — Supplementary Materials and Methods and Results and Discussion. (DOCX) [file pone.0032481.s006.docx]

Supplementary Materials and Methods and Results and Discussion

*Monaco et al., 2011*

Contents

[Microarray protocol 3](#_Toc298943070)

[Synthesis of cDNA 3](#_Toc298943071)

[cDNA cleaning 3](#_Toc298943072)

[cDNA labeling 3](#_Toc298943073)

[Microarray slide preparation and prehybridization 3](#_Toc298943074)

[Hybridization 4](#_Toc298943075)

[Washing and scanning of slides and analysis of data 4](#_Toc298943076)

[*Sus scrofa* oligo microarray features 4](#_Toc298943077)

[Additional strategies used for Ingenuity Pathway Analysis 5](#_Toc298943078)

[Criteria used to interpret the IPA functional analysis 5](#_Toc298943079)

[Criteria used to interpret the IPA Canonical Pathways 6](#_Toc298943080)

[Additional results and discussion 7](#_Toc298943081)

[Highly abundant transcripts 7](#_Toc298943082)

[MSC vs. fully differentiated tissues 8](#_Toc298943083)

[Overall DEG between ASC and BMSC during the whole experiment 9](#_Toc298943084)

[Osteogenic and adipogenic gene markers 10](#_Toc298943085)

[TABLE S.1 – Pearson correlation between ASC and BMSC transcriptome 12](#_Toc298943086)

[TABLE S.2. Function analysis results by IPA of adipogenic and osteogenic differentiation of ASC at dd2. 13](#_Toc298943087)

[TABLE S.3. Function analysis results by IPA of adipogenic and osteogenic differentiation of ASC at dd21 15](#_Toc298943088)

[TABLE S.4. Function analysis results by IPA of adipogenic and osteogenic differentiation of BMSC at dd21 16](#_Toc298943089)

[TABLE S.5. Pearson correlation between ASC and BMSC transcriptome in each pig and for each time point during adipogenic and osteogenic differentiation 17](#_Toc298943090)

[TABLE S.6. Pearson correlation between pigs (12, 22, and 40) transcriptome in each cell type 18](#_Toc298943091)

[TABLE S.7. Function analysis results by IPA of BMSC and ASC during adipogenic differentiation at dd2 19](#_Toc298943092)

[TABLE S.8. Function analysis results by IPA of BMSC and ASC during adipogenic differentiation at dd7 21](#_Toc298943093)

[TABLE S.9. Function analysis results by IPA of BMSC and ASC during adipogenic differentiation at dd21 22](#_Toc298943094)

[TABLE S.10. Function analysis results by IPA of BMSC and ASC during osteogenic differentiation at dd2 23](#_Toc298943095)

[TABLE S.11. Function analysis results by IPA of BMSC and ASC during osteogenic differentiation at dd21 25](#_Toc298943096)

[*Figure S1. Pearson correlation between human ASC and BMSC in data from Jansen et al.* 26](#_Toc298943097)

[*Figure S2. Number of cells before starting differentiation and during the adipogenic and osteogenic differentiation in porcine ASC and BMSC*. 27](#_Toc298943098)

[*Figure S3. Significant enriched pathways between adipogenic and osteogenic differentiation in ASC and BMSC*. 28](#_Toc298943099)

[*Figure S4. Quantity of RNA per cell (ng) before starting differentiation and during the adipogenic and osteogenic differentiation in porcine ASC and BMSC*. 29](#_Toc298943100)

[*Figure S5. Significant enriched pathways between ASC and BMSC during adipogenic and osteogenic differentiation.* 30](#_Toc298943101)

[REFERENCES 31](#_Toc298943102)

# Microarray protocol

Synthesis of cDNA. The cDNA synthesis was carried out with a total of 10 μg of RNA (~1 μg/μl). The RNA was mixed with 2 μl of random hexamer primers (Invitrogen: 3 mg/ml) and 1 μl oligo dT18 (1 μg/μl; Operon Biotechnologies), and taken to a final volume of 17.78 μl with RNase-free water. The mixture was mixed and incubated at 70^o^C for 10 min in order to allow dissociation between partial-complementary RNA (dimerization) and dissociation of complementary bases in the same RNA (i.e., harping and self-dimerization). After incubation, the tubes were placed on ice for at least 3 min to avoid coiling of RNA. Each sample had 12.2 μl of a master mix added. The master mix was prepared with: 6.0 μl of 5X First Strand Buffer, 3.0 μl of 0.1 MDTT, 0.6 μl of 50X dNTP-dUTP, 0.12 μl of 50 mM aa-UTP, 2.0 μl of SuperScript III RT (200 U/μl; Invitrogen), and 0.5 μl of RNase inhibitor (10,000 U/μl; Promega). The preparation was mixed and incubated at 23^o^C for 1 min, and then at 46^o^C for 9 h. After the incubation period, the samples were kept at 4^o^C to preserve the integrity of the DNA synthesized.

cDNA cleaning. Cleaning was performed using Qiaquick PCR purification kit (Qiagen). Ten μl of 1 M NaOH were added to the synthesized cDNA to remove residual RNA, and then incubated at 65^o^C for 15 min. To neutralize pH and avoid damage of the columns, 10 μl of 1M HCl were added. To remove unincorporated aa-dUTP and free amines, a purification protocol modified from Qiagen Qiaquick PCR purification kit was used. Cleaned cDNA was collected in 100 µl Phosphate elution buffer and concentration was measured by NanoDrop ND-100 ([www.nanodrop.com](http://www.nanodrop.com)) in order to verify efficiency of the cDNA synthesis.

cDNA labeling. Methods for aminoallyl-labeling and cleanup of cDNA were described previously[^1^](#_ENREF_1). Briefly, the aminoallyl-labeled cDNA samples were dried using a speed-vac (Eppendorf Vacufuge® Concentrator) for ~1 h and then resuspended in 4.5 μl 0.1 M sodium carbonate buffer (pH = 9.0). Four and a half μl of the appropriate Cy dye ester (Cy3 or Cy5; Amersham) was added to couple the aa-cDNA and the samples were incubated for at least 1 h at room temperature. Removal of uncoupled dye was done using the Qiagen Qiaquick PCR purification kit. Labeled cDNA was specked using NanoDrop ND-100. Only samples with ≥ 3 μg of cDNA and ≥ 300 pMol of labeling were used for hybridization. All experimental samples had to be co-hybridized with the same amount of cDNA of the reference labelled with opposite dye. For the purpose, the same amount of samples and references cDNA were transferred in new 1.5 ml tubes and vacuum dried. Samples and references were then ready for hybridization.

Microarray slide preparation and prehybridization. Prior hybridization slides were rehydrated, treated at the UV crosslinker, washed with 0.2% SDS solution, deeply rinsed with purified water, and pre-hybridized using a solution containing 1% albumin, 5 × SCC, and 0.1% SDS at 42°C for ≥ 45 min. After pre-hybridization slides were rinsed with abundant purified water and immerged in isopropanol for ~10 s and spin dried. Dried slides were immediately hybridized.

Hybridization. Slides were hybridized according to a dye-swap-reference design (i.e. each sample was labeled twice using the two dyes and hybridized in each slide with the reference labeled with the opposite dye). Labeled cDNA of the sample was re-hydratated with 80µl of hybridization buffer #1 (Ambion) and mixed thoroughly. The same solution was transferred in the reference with opposite dye and mixed in order to obtain a homogenous solution of the two labeled cDNA. Probe mixture was denatured at 95°C for 3 min and injected under the coverslip of the slide. Hybridization was carried out using humidified slide chamber (Corning) at 42°C for about 40 hours in dark.

Washing and scanning of slides and analysis of data**.** After disassembling of the hybridization chamber, slides were transferred to a solution of 1X SSC, 0.2% SDS at 42°C. Slides were then washed with agitation in 0.1X SSC and 0.2% SDS for 5 min. Slides were next transferred to a new tray and washed with agitation for 5 min in 0.1X SSC at room temperature. An Axon 4000B scanner was used to collect the data in conjunction with GenPix 6.0 software.

# *Sus scrofa* oligo microarray features

Microarray used for hybridization consist in a novel 13,297 (excluding control spots) pig specific oligonucleotide set developed by Qiagen–Operon and the USDA-NRSP-8 Swine Genome community collaboration[^2^](#_ENREF_2). The Qiagen oligonucleotide set represents porcine cDNA and EST, designed from the Institute of Genome Research (TIGR) Tentative Consensus cDNA sequences. Synthesized oligonucleotides were printed at the W.M. Keck Center microarray facility (University of Illinois at Urbana-Champaign).

Synthesized oligos were reconstituted at 40 μM in double distilled H_2_O using a Biomek FX robot (Beckman Coulter, Fullerton, CA), and allowed to rehydrate overnight at 4°C. Microarray printing plates were created by aliquoting 5 μl of the oligo solution into 5 μl 300 mM NaPO4 buffer (pH 8.5). Oligos were printed on GAPS II slides (Corning, Corning, NY) using a GeneMachines OmniGrid 100 microarrayer (Genomic Solutions, Ann Arbor, MI) at 65% humidity. Oligos were printed in duplicate side by side, using a 4 × 12 print design, a center-to-center distance of 180 μm, and a spot diameter of 100 μm. Control oligos were located in printing plate 1 and subsequently spread over all blocks. Each block also contained at least 2 spots of buffer, control DNA, and polyA. The total number of spots, including control spots, on the array was 28,800. After printing, arrays were left on the microarrayer overnight in 65% humidity and stored in vacuum-sealed containers until use.

In the latest up-to-date annotation of the pig array (December 2007) 99.4% of oligos (13,228 oligos) were annotated (i.e. with either a gene symbol, Entrez Gene ID, or Unigene). Of those 73.3% were unique gene symbol, 78% unique Unigene ID, and 79.6% unique Entrez Gene ID, and 20.8% of annotated genes had redundancy (16.2% genes with 2 replicates, 4.6% with >2 replicates). In addition, there were 358 control oligos (e.g. genomics DNA and polyA) and 363 duplicates spots for blank and only buffer.

# Additional strategies used for Ingenuity Pathway Analysis

The IPA knowledge base provides Core Analysis results which include diseases and additional functions that cannot be considered biologically relevant in the present analysis; therefore, several functions have been eliminated from the final results. The eliminated functions were: all Diseases and Disorders, Auditory and Vestibular System Development and Function, Behavior, Digestive System Development and Function, Hepatic System Development and Function, Renal and Urological System Development and Function, Respiratory System Development and Function, and Visual System Development and Function.

## Criteria used to interpret the IPA functional analysis

The description of the functions in IPA includes the response of the genes (⇑ or ⇓) and the “effect on function” feature in IPA. The final evaluation on the effect on the function reported in Figures 3 and 5 of the main body of the manuscript and effect on the pathways reported in Figures S3 and S5 was given following these criteria:

- genes that were ⇑ and were associated with “decrease function” (in IPA) were considered as actively decreasing or inhibiting the function;
- genes that were ⇓ and were associated with “decrease function” were considered as failing to decrease the particular function, those were deemed to allow the function to happen;
- genes that were ⇑ and were associated with the category of “increase function” were considered as being able to increase or induce the particular function;
- genes that were ⇓ and were associated with the category of “increase function” were deemed as failing to increase or induce the particular function;
- the final evaluation was reached by taking into account the sum of all ⇑ and ⇓ regulated genes;
- several genes in IPA were considered to decrease or increase a particular function when in fact just the opposite was true, mostly because IPA often does not distinguish between cell/tissue/entire organism (e.g., “CD36 or LPL decrease quantity of lipids” which is true if the point of reference is the plasma, but if the point of reference is the cell then they increase quantity of lipid). In this case, based on our biological knowledge, even though the gene was included in the category of “decrease function” we considered it as part of the “increase function”.

Following the above criteria the final judgment on the direction of the function was finalized using the following approach:

- when the “effect on function” had a number of genes within “increase/decrease function” that was <10% greater than those of “decrease/increase function” the function was considered to be in equilibrium or without a net effect. Thus, even though the function was significantly enriched in the DEG, a conclusive judgment on a biological outcome was not feasible and the function was considered in equilibrium (denoted by ⇔).
- when “effect on function” had a number of genes within “increase/decrease function” that was ≥10% greater than those of “decrease/increase function” including genes in “affect function” the function was considered as “tends to increase/decrease (or induce/inhibit)” which was denoted with arrows (tendency to induce or increase = **↑**; tendency to inhibit or decrease = **↓**).
- when the number of genes considered to increase/induce or decrease/inhibit a particular function was ≥100% (or ≥2-fold) compared with the number of genes considered to decrease/inhibit or increase/induce, the function was considered evidently induced or inhibited (simple arrows **⇑** or **⇓**).
- when all, or nearly all, the genes were considered to increase/induce or decrease/inhibit a particular function, or the analysis of “affect function” denoted them as involved in inducing or inhibiting the function, the overall function was considered to be completely induced or inhibited (**⇑⇑** or **⇓⇓**).

## Criteria used to interpret the IPA Canonical Pathways

The canonical pathways interpretation was straighter forward due to the well-determined biological meaning of the pathways. However, very rarely the DEG in the pathways were all consistently ⇑ or ⇓ and a careful interpretation using the overall effect on the pathway was required. The interpretation of those pathways was based mainly on the possible final output of the overall DEG in the pathway. For example, if the pathway was labeled “LPS/IL-1-Mediated Inhibition of RXR Function” the interpretation was based on the actual inhibition of RXR function as suggested by the overall expression pattern of the genes, e.g., if the genes coding for protein in the LPS/IL-1 signaling for the inhibition of RXR were mostly ⇓, then the inhibition was not induced (or not taking place). The interpretation is not simply based on the sum of ⇑ and ⇓ DEG in the pathway.

# Additional results and discussion

## Highly abundant transcripts

To individuate the genes with a medium-high expression in both MSC an analysis using GeneSpring GX7 was run. Only genes with a relative fluorescent unit (RFU) or intensity signal >1,000 were taken into account. This intensity was set because it corresponds to the average RFU for the *GAPDH* gene that can be considered a medium expressed gene. However, this method has several limitations because the RFU depends on the abundance of the original transcript in the samples, on the specificity of binding between the labeled cDNA and printed oligo, on the amount of printed oligo after slide washing, and on the efficiency of cDNA labeling. Despite those limitations, a high RFU signal can be considered an acceptable indicator of transcript abundance. With RFU >1,000 as threshold, 960 unique annotated transcripts (1,041 oligos) were considered being present at a medium-high abundance (file S3, Sheet 1). The functional chart analysis of those transcripts using DAVID (file S3, Sheet 2) indicated that the most enriched terms were several TFBS, ribosomal components, collagen and fibrin. Among the TFBS the most enriched were GATA2 (GATA binding protein 2), a transcription factor specifically expressed in pre-adipocytes and with a role in adipogenesis^[3](#_ENREF_3)^, PAX5 (paired box gene 5) which has been shown to have an important role in immunosuppression capacity of mesenchymal stem cells[^4^](#_ENREF_4), and NRSF (RE1-silencing transcription factor) which has a pivotal role in suppressing neurogenesis in non-neural tissues[^5^](#_ENREF_5). The functional cluster analysis in DAVID (file S3, Sheet 3) uncovered that extracellular components, chiefly collagen, regulation of apoptosis, nucleotide metabolism, and immune suppression were the terms with the highest enrichment score[^6^](#_ENREF_6). In IPA no functions or pathways were significantly enriched with a threshold of B-H FDR ≤0.05.

When the RFU threshold was set to 10,000 in order to uncover the most highly expressed genes in the two types of MSC, 145 unique annotated transcripts (174 oligos) passed the threshold (file S3, Sheet 1). The functional analysis in DAVID identified components of the extracellular matrix (e.g., hydroxyproline, collagen), angiogenesis (e.g., platelet-derived growth factor binding, blood circulation), differentiation (of muscle, adipose, heart), and regulation of inflammation being the most enriched terms (file S3, Sheets 4 and 5). In IPA no functions were significantly enriched with B-H FDR≤0.05, and only one pathway (Intrinsic Prothrombin Activation) was significantly enriched but with only 4 genes. The visual analysis of the pathway (file S3, Sheet 6) indicated that the highly abundant transcripts in MSC were part of the first steps in regulating blood clothing after vessel damage.

All those data suggest that the transcriptome of the two MSC from porcine evaluated in the present experiment is characterized by a high abundance of mRNA coding for proteins with roles in extracellular matrix formation, differentiation, protein synthesis, immune suppression, and cell cycle regulation. In addition, the analysis suggests several TFBS being important in those cells, with the PAX5 playing a major role in the transcriptome, particularly in the immune suppression capacity of those cells, and NRSF probably playing a role in suppressing neurogenesis in those cells.

## MSC vs. fully differentiated tissues

The microarray were run with a dye-swap reference design using as reference a mixture of fully differentiated tissues (see above for details in microarray analysis protocol). Those conditions allowed for a direct transcriptomics comparison between undifferentiated MSC and fully differentiated tissues, with the identification of transcripts and related functions typical of the MSC compared to adult tissues. To identify DEG between MSC and fully differentiated tissues we run a statistical analysis using t-test with B-H FDR adjustment for multiple comparisons available in GeneSpring GX7. The method presented some limitations in fact, the reference used in our microarray analysis was highly enriched of endoderm-origin tissues (i.e., ca. 30% liver and ca. 30% jejunum) while underrepresented was the ectoderm (mammary gland was only 0.7%) and mesoderm (kidney and part of the mammary gland). Moreover, the use of a mixture of tissues tends to dilute highly abundant and tissue-specific transcripts. Thus, the comparison suffers for such unbalance. Despite this limitation, the mixture of mRNA used as reference can be considered representative of adult tissue-like transcriptome.

Using a cut-off of B-H FDR≤0.001 and a >2-fold differences between sample and reference we found 1,356 oligos differentially expressed in MSC *vs.* fully differentiated tissues. Among those 485 unique genes were >2-fold lower expressed and 706 unique genes >2-fold higher expressed in the MSC compared to the reference (file S4, Sheet 1).

The genes with >2-fold expression in MSC *vs.* reference had protein synthesis and components of protein synthesis machinery as the most significantly enriched terms in both DAVID and IPA (file S4, Sheet 2), which indicates a large protein synthesis in the MSC compared to adult tissues. Interestingly, the most significant enriched UP-tissue terms were Cajal-Retzius cells and fetal embryo cortex (file S4, Sheet 2). The Cajal-Retzius cells are cells with a primary role in the implementation of neuronal cortical circuits during development[^7^](#_ENREF_7). The reason for the high enrichment of these terms with >2-fold larger expressed genes in MSC than adult tissues is not apparent, but might suggests a similar function of the MSC as for Cajal-Retzius cells in the neuronal development. In support of this it has been shown that the use of mesenchymal stem cells improves neurogenesis in spinal cord injuries[^8^](#_ENREF_8)^,^[^9^](#_ENREF_9). The data however suffer for the absence in our microarray reference of any neuronal tissue, which might have caused for the enrichment of neuronal-related genes in the stem cells. The extracellular matrix together with cytoskeleton components was also among the most significantly enriched terms (file S4, Sheet 2). The functional cluster with the largest score included the above terms and uncovered also an overrepresentation of terms related to carbohydrate metabolism (i.e., glycolysis), cell adhesion, and migration among the terms with an EASE score <0.1 (file S4, Sheet 3).

The IPA analysis confirmed most of the findings with DAVID and uncovered a significant enrichment (B-H FDR<0.05) of cell death and proliferation (file S4, Sheet 4). The effect on functions analysis in IPA indicated that most of the genes which enriched cell death tended to inhibit it and the genes that enriched cell proliferation tended to induce the increase in cell number (file S4, Sheet 5). In Addition, the effect on functions in IPA clearly indicated larger protein synthesis in MSC compared to fully differentiated tissues. IPA uncovered several pathways significantly (B-H FDR<0.05) enriched by the genes more expressed in MSC compared to adult tissues; all those pathways, except IGF1 signaling which is involved in cell proliferation, are typical of cytoskeleton organization for cellular migration (see depiction of the pathways in file S4, Sheet 6).

The genes with lower expression in MSC compared to the differentiated tissues enriched in DAVID significantly terms related to liver, coagulation, oxidoreductase, activation of immune response (including also major histocompatibility complex), and lipid metabolism (file S4, Sheets 7 and 8). The high enrichment of liver is not surprising due to the >30% RNA from porcine liver in the reference. Moreover, the gene with the lowest ratio was albumin (i.e., highest expression in reference *vs.* sample; file S4, Sheet 1), which is a protein produced exclusively in the liver. Interesting was the high enrichment of immune response translated, in agreement with what previously reported[^10^](#_ENREF_10), in a low, or even decreased, activation of the immune response by MSC compared to differentiated tissues (e.g., lower histocompatibility complex activity). The enrichment of lipid metabolism by lower expressed genes in MSC compared to the reference might be partly due to the presence of tissues with high lipid metabolism in the latter, such as liver, but can also indicate a low lipid metabolism of undifferentiated cells.

The IPA analysis confirmed most of the findings with DAVID highlighting, as the most enriched functions, lipid metabolism and immune response (file S4, Sheet 9). The effect on functions in IPA confirmed a lower lipid metabolism and immune response and its activation in MSC compared to the differentiated tissues (file S4, Sheet 10). IPA uncovered several enriched pathways in genes more expressed in fully differentiated tissue vs. MSC (file S4, Sheet 11). Most of those pathways appeared to be typical of the liver (e.g., urea cycle, acute phase response, bile acid biosynthesis), but enriched were also several pathways related to lipid metabolism (e.g., fatty acid metabolism and activation of lipid related functions through FXR/RXR), xenobiotic metabolism (e.g.,involving P450 and xenobiotic metabolism), and several amino acids metabolism (e.g., tryptophan, arginine, proline, and tyrosine).

## Overall DEG between ASC and BMSC during the whole experiment

A total of 579 DEG (528 unique annotated genes or 4.3% of all oligos coding for genes in the microarray) with a B-H FDR≤0.05 for overall cell type effect was uncovered. This means the genes were overall different considering the whole experiment (i.e., prior differentiation plus during the whole differentiation). Functional analysis of those genes is reported in file S9, Sheet 1. Out of the 528 unique annotated genes, 280 were overall more expressed in ASC *vs.* BMSC and 248 more expressed in BMSC *vs.* ASC. The functional analysis of the 528 genes indicated that terms related to cell morphology, adhesion, extracellular matrix, and cell movement were among the most enriched (file S9, Sheets 2-6). In DAVID several TFBS were significantly enriched in this gene list, with OCT, and specifically POU6F1, being the most enriched. The POU6F1 appears to act as a repressor of POU2F2 (a.k.a OCT2A) mediated activation[^11^](#_ENREF_11). The POU2F2 is a transcription factor that induces expression of immunoglobulin genes[^12^](#_ENREF_12). Those observations suggest that the two MSC present distinctions during differentiation which encompass chiefly cell movement and cell spreading, and probably they differ in the regulation of immune-related genes.

The functional analysis in DAVID for the genes more expressed in ASC *vs.* BMSC during the whole experiment uncovered a significant enrichment of actin cytoskeleton components, adhesion, regulation of cell movement, and OCT and POU6F1 among the TFBS (file S9, Sheets 7 and 8). IPA analysis confirmed major finding by DAVID analysis (file S9, Sheets 9 and 10). Several pathways in IPA were significantly enriched (B-H FDR<0.05, file S9, Sheet 11). Those pathways were mostly associated with cytoskeleton organization, cell adhesion and movement, with significant enrichment of VEGF and CXCR4 signaling among others. Those data suggest an overall higher migration capacity and cellular remodeling for ASC *vs.* BMSC during the whole experiment.

The most significantly enriched terms in DAVID among genes overall more expressed in BMSC *vs.* ASC during the whole experiment were related to protein synthesis and fibronectin (file S9, Sheets 12 and 13). In IPA there were not functions or pathways significantly enriched (B-H FDR<0.05) with the exception of the pathway Butanoate Metabolism (file S9, Sheet 14). The analysis suggests that the BMSC *vs.* ASC were experiencing higher protein synthesis and production of fibronectin.

## Osteogenic and adipogenic gene markers

Based on previous morphological and transcriptomics characterization of the samples used for this experiment, both MSC are able to differentiate into adipocytes or osteocytes but with some differences[^13^](#_ENREF_13). To uncover specific gene markers for osteogenesis and adipogenesis we used the list of genes with FDR≤0.05 for the cell type × time × differentiation interaction, and followed the following criteria:

- the gene has to increase significantly (p<0.001) the expression ≥2-fold during the differentiation compared to the undifferentiated state (i.e., dd0); the increase in expression compared to dd0 has to be unique in one type of differentiation compared to the other (for this the gene has to increase ≥2-fold in one differentiation and ≤1.5 fold in the other differentiation; the difference in expression for the same gene between the two differentiation for each time point has to be significant at p<0.001).

Every specific marker was uncovered using these supplementary criteria:

- for **overall best adipogenic** or **osteogenic markers** expression of the genes needs to be overall ≥2-fold significantly (p<0.001) different in all time points compared to dd0 in adipogenic or osteogenic differentiation with a 2-fold significant (p<0.001) difference between the two differentiations;
- for **best adipogenic** or **osteogenic markers specifically in ASC** or **BMSC** overall expression of the genes needs to be ≥2-fold significantly (p<0.001) different in all time points compared to dd0 in adipogenic or osteogenic differentiation with a 2-fold significant (p<0.001) difference between the two differentiations in all time points considering only ASC or BMSC, respectively;
- for **good adipogenic markers specifically** **in ASC** or **BMSC** expression of the genes needs to be ≥2-fold significant (p<0.001) different in at the least in 2 time points compared to dd0 in adipogenic or osteogenic differentiation with a 2-fold significant (p<0.001) difference between the two differentiations in all time points considering only ASC or BMSC, respectively;
- for **weak adipogenic markers specifically** **in ASC** or **BMSC** expression of the genes needs to be ≥2-fold significant (p<0.001) different in at the least 1 time points compared to dd0 in adipogenic or osteogenic differentiation with a 2-fold significant (p<0.001) difference between the two differentiations in all time points considering only ASC or BMSC, respectively;
- in addition, because the differentiation is a chronological phenomenon, we have uncovered the **best adipogenic** or **osteogenic markers for the medium-late differentiation (after a week of differentiation; i.e, dd7 and dd21)** as the genes with an expression be ≥2-fold significant (p<0.001) different in both time points compared to dd0 in adipogenic or osteogenic differentiation with a 2-fold significant (p<0.001) difference between the two differentiations in both time points. The same analysis was run considering only ASC or BMSC alone;
- for each time point and for each time point in each MSCs markers were uncovered using the same criteria as above.

The overall abundance of the transcript can be an interesting feature in order to select an appropriate marker with low-sensitive techniques. For this in file S10 was provided also the initial expression in Relative Fluorescent Unit (RFU) which is, with several limitations (see main manuscript) the indication of transcript abundance. Reported are the RFU>1,000, as below this the genes can be considered medium-low expressed based on expression of *GAPDH* (ca. 1,000 RFU).

A complete list of those markers and the results of functional analysis using DAVID is provided in file S10 (Sheets 1-15). The use of selected criteria allowed the identification of NAD(P)H dehydrogenase quinone 1 (*NQO1*) as the best overall marker of adipogenesis. No overall best marker was uncovered for the osteogenic differentiation. We uncovered 39 markers for adipogenesis and 65 for osteogenesis. Aquaporin 3 (*AQP3*), stearoyl-CoA desaturase (*SCD*), fatty acid binding protein 3 and 5 (*FABP3* and *FABP5*), and ferritin light polypeptide (*FTL*) were among the best adipogenic markers after *NQO1*. Hemopexin (*HPX*), collagenase type 3 α1 (*COL3A1*), annexin A8-like 1 (*ANXA8L1*), flotillin 2 (FLOT2), and periostin or osteoblast specific factor (*POSTN*) were among the best osteogenic markers.

Functional analysis of the markers uncovered a significant enrichment of PPAR signaling pathways and other lipid-related functions among adipogenic markers, and extracellular matrix related functions among osteogenic markers (file S10, Sheets 2-7). Adipogenic and osteogenic markers for each time point comparison are reported in file S10 (Sheet 1) and DAVID analysis of those markers also is reported in file S10 (Sheets 8-15).

A complete list of those markers and the results of functional analysis using DAVID is provided in file S10 (Sheets 1-15). The use of selected criteria described above allowed the identification of NAD(P)H dehydrogenase quinone 1 (*NQO1*) as the best overall marker of adipogenesis. No overall best marker was uncovered for the osteogenic differentiation. We uncovered 39 markers for adipogenesis and 65 for osteogenesis. Aquaporin 3 (*AQP3*), stearoyl-CoA desaturase (*SCD*), fatty acid binding protein 3 and 5 (*FABP3* and *FABP5*), and ferritin light polypeptide (*FTL*) were among the best adipogenic markers after *NQO1*. Hemopexin (*HPX*), collagenase type 3 α1 (*COL3A1*), annexin A8-like 1 (*ANXA8L1*), flotillin 2 (FLOT2), and periostin or osteoblast specific factor (*POSTN*) were among the best osteogenic markers.

Functional analysis of the markers uncovered a significant enrichment of PPAR signaling pathways and other lipid-related functions among adipogenic markers, and extracellular matrix related functions among osteogenic markers (file S10, Sheets 2-7). Adipogenic and osteogenic markers for each time point comparison are reported in file S10 (Sheet 1) and DAVID analysis of those markers also is reported in file S10 (Sheets 8-15).

# REFERENCES

1 Loor, J. J. *et al.* Temporal gene expression profiling of liver from periparturient dairy cows reveals complex adaptive mechanisms in hepatic function. *Physiol Genomics* **23**, 217-226, doi:00132.2005 [pii]

10.1152/physiolgenomics.00132.2005 (2005).

2 Zhao, S. H. *et al.* Validation of a first-generation long-oligonucleotide microarray for transcriptional profiling in the pig. *Genomics* **86**, 618-625, doi:S0888-7543(05)00213-2 [pii]

10.1016/j.ygeno.2005.08.001 (2005).

3 Tong, Q., Tsai, J. & Hotamisligil, G. S. GATA transcription factors and fat cell formation. *Drug News Perspect* **16**, 585-588, doi:429 [pii] (2003).

4 Rafei, M. *et al.* Mesenchymal stromal cell-derived CCL2 suppresses plasma cell immunoglobulin production via STAT3 inactivation and PAX5 induction. *Blood* **112**, 4991-4998, doi:blood-2008-07-166892 [pii]

10.1182/blood-2008-07-166892 (2008).

5 Lunyak, V. V. & Rosenfeld, M. G. No rest for REST: REST/NRSF regulation of neurogenesis. *Cell* **121**, 499-501, doi:DOI 10.1016/j.cell.2005.05.003 (2005).

6 Huang da, W., Sherman, B. T. & Lempicki, R. A. Systematic and integrative analysis of large gene lists using DAVID bioinformatics resources. *Nat Protoc* **4**, 44-57, doi:nprot.2008.211 [pii]

10.1038/nprot.2008.211 (2009).

7 Mienville, J. M. Cajal-Retzius cell physiology: just in time to bridge the 20th century. *Cereb Cortex* **9**, 776-782 (1999).

8 Park, H. W. *et al.* Human mesenchymal stem cell-derived Schwann cell-like cells exhibit neurotrophic effects, via distinct growth factor production, in a model of spinal cord injury. *Glia* **58**, 1118-1132, doi:10.1002/glia.20992 (2010).

9 Oh, J. S. *et al.* Hypoxia-preconditioned adipose tissue-derived mesenchymal stem cell increase the survival and gene expression of engineered neural stem cells in a spinal cord injury model. *Neurosci Lett* **472**, 215-219, doi:S0304-3940(10)00154-0 [pii]

10.1016/j.neulet.2010.02.008 (2010).

10 Ghannam, S., Bouffi, C., Djouad, F., Jorgensen, C. & Noel, D. Immunosuppression by mesenchymal stem cells: mechanisms and clinical applications. *Stem Cell Res Ther* **1**, 2, doi:scrt2 [pii]

10.1186/scrt2 (2010).

11 Wey, E. & Schafer, B. W. Identification of novel DNA binding sites recognized by the transcription factor mPOU (POU6F1). *Biochem Biophys Res Commun* **220**, 274-279, doi:S0006-291X(96)90395-2 [pii]

10.1006/bbrc.1996.0395 (1996).

12 Salas, M. & Eckhardt, L. A. Critical role for the Oct-2/OCA-B partnership in Ig-secreting cells. *J Immunol* **171**, 6589-6598 (2003).

13 Monaco, E. *et al.* Morphological and Transcriptomic Comparison of Adipose and Bone Marrow Derived Porcine Stem Cells. *The Open Tissue Engineering & Regenerative Medicine Journal*, 20-33, doi:10.2174/1875043500902010020 (2009).
