# Supplementary material for: Transcriptomics Comparison between Porcine Adipose and Bone Marrow Mesenchymal Stem Cells during In Vitro Osteogenic and Adipogenic Differentiation
Source: PLoS One. 2012 Mar 7;7(3):e32481. doi: 10.1371/journal.pone.0032481 (PMC3296722; doi:10.1371/journal.pone.0032481)

PATHWAY RESULTS FROM INGENUITY PATHWAY ANALYSIS

Contents

[OSTEOGENIC vs. ADIPOGENIC DIFFERENTIATION IN ASC 2](#_Toc298936352)

[dd7 2](#_Toc298936353)

[Overall statistic 2](#_Toc298936354)

[α-adrenergic signaling 3](#_Toc298936355)

[CCR5 signaling in Macrophages 4](#_Toc298936356)

[Relaxin signaling 5](#_Toc298936357)

[OSTEOGENIC vs. ADIPOGENIC DIFFERENTIATION IN ASC 6](#_Toc298936358)

[dd21 6](#_Toc298936359)

[Overall statistic 6](#_Toc298936360)

[Actin cytoskeleton signaling 7](#_Toc298936361)

[α adrenergic signaling 8](#_Toc298936362)

[Axonal guidance signaling 9](#_Toc298936363)

[CCR3 signaling in Eosinophil 10](#_Toc298936364)

[CCR5 signaling in Macrophages 11](#_Toc298936365)

[Chemokine signaling 12](#_Toc298936366)

[Ephrin receptor signaling 13](#_Toc298936367)

[Fatty acid biosynthesis 14](#_Toc298936368)

[fMLP signaling in Neutrophils 15](#_Toc298936369)

[Glutamate receptor signaling 16](#_Toc298936370)

[LPS/IL-1 Mediated Inhibition of RXR Function 17](#_Toc298936371)

[Regulation of Actin-based Motility by Rho 18](#_Toc298936372)

[OSTEOGENIC vs. ADIPOGENIC DIFFERENTIATION IN BMSC 19](#_Toc298936373)

[dd21 19](#_Toc298936374)

[LPS/IL-1 Mediated Inhibition of RXR Function 19](#_Toc298936375)

[BMSC vs. ASC DURING OSTEOGENIC DIFFERENTIATION 20](#_Toc298936376)

[dd2 20](#_Toc298936377)

[14-3-3 mediated signaling 20](#_Toc298936378)

[dd21 21](#_Toc298936379)

[Keratan sulfate biosynthesis 21](#_Toc298936380)

[LPS/IL-1 Mediated Inhibition of RXR Function 22](#_Toc298936381)

[Sulfur metabolism 23](#_Toc298936382)

[NETWORK AMONG DEG 24](#_Toc298936383)

[OSTEOGENIC vs. ADIPOGENIC DIFFERENTIATION IN ASC 24](#_Toc298936384)

[dd2 24](#_Toc298936385)

[dd7 25](#_Toc298936386)

[dd21 26](#_Toc298936387)

[OSTEOGENIC vs. ADIPOGENIC DIFFERENTIATION IN BMSC 27](#_Toc298936388)

[dd2 27](#_Toc298936389)

[dd7 28](#_Toc298936390)

[dd21 29](#_Toc298936391)

[BMSC vs. ASC DURING OSTEOGENIC DIFFERENTIATION 30](#_Toc298936392)

[dd2 30](#_Toc298936393)

[BMSC vs. ASC DURING ADIPOGENIC DIFFERENTIATION 31](#_Toc298936394)

[dd2 31](#_Toc298936395)

[dd7 32](#_Toc298936396)

# OSTEOGENIC vs. ADIPOGENIC DIFFERENTIATION IN ASC

## dd7

### Overall statistic

| Ingenuity Canonical Pathways | -Log(P-value) | -Log(B-H P-value) | Ratio |
| --- | --- | --- | --- |
| CCR5 Signaling in Macrophages | 4.29 | 2.21 | 0.0706 |
| Α-Adrenergic Signaling | 3.79 | 2.02 | 0.0686 |
| Relaxin Signaling | 3.54 | 1.94 | 0.0515 |
| Cardiac Hypertrophy Signaling | 2.76 | 1.37 | 0.0364 |
| IL-8 Signaling | 2.67 | 1.37 | 0.0391 |
| Lysine Degradation | 2.36 | 1.14 | 0.0286 |
| Glutamate Receptor Signaling | 2.28 | 1.14 | 0.0476 |
| Cardiac β-adrenergic Signaling | 2.27 | 1.14 | 0.0394 |
| CXCR4 Signaling | 2.18 | 1.12 | 0.0373 |
| CCR3 Signaling in Eosinophils | 2.11 | 1.12 | 0.0435 |
| fMLP Signaling in Neutrophils | 2.09 | 1.12 | 0.0400 |
| Melatonin Signaling | 2.09 | 1.12 | 0.0526 |
| G-Protein Coupled Receptor Signaling | 2.03 | 1.12 | 0.0294 |
| Nitric Oxide Signaling in the Cardiovascular System | 2.03 | 1.12 | 0.0476 |
| cAMP-mediated Signaling | 1.88 | 1.00 | 0.0325 |
| LPS/IL-1 Mediated Inhibition of RXR Function | 1.83 | 0.99 | 0.0266 |
| Cellular Effects of Sildenafil (Viagra) | 1.73 | 0.92 | 0.0308 |
| CDK5 Signaling | 1.71 | 0.92 | 0.0455 |
| Role of NFAT in Regulation of the Immune Response | 1.57 | 0.80 | 0.0272 |
| Sonic Hedgehog Signaling | 1.55 | 0.80 | 0.069 |
| PXR/RXR Activation | 1.54 | 0.80 | 0.0357 |
| PPARÎ±/RXRÎ± Activation | 1.41 | 0.70 | 0.0276 |
| Pyrimidine Metabolism | 1.40 | 0.70 | 0.0177 |
| Thrombin Signaling | 1.34 | 0.69 | 0.0259 |
| Xenobiotic Metabolism Signaling | 1.34 | 0.69 | 0.0206 |
| Ephrin Receptor Signaling | 1.33 | 0.69 | 0.0270 |

**OSTEOGENIC vs. ADIPOGENIC DIFFERENTIATION IN ASC**

**dd7**

Red and green shade in objects denote higher expression in adipogenic and osteogenic, respectively

### α-adrenergic signaling


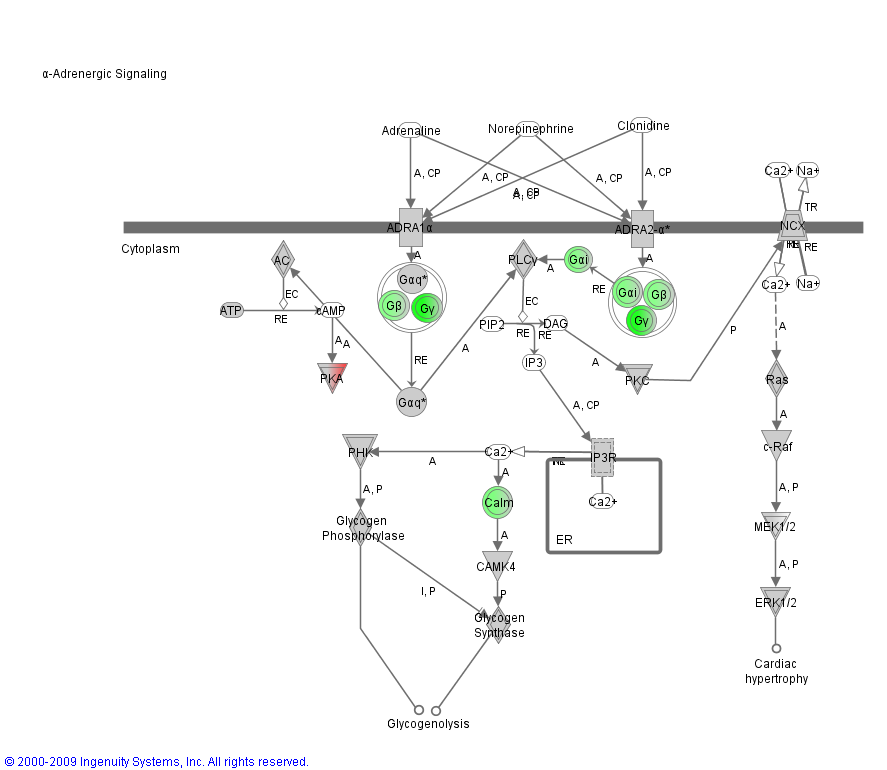


**OSTEOGENIC vs. ADIPOGENIC DIFFERENTIATION IN ASC**

**dd7**

Red and green shade in objects denote higher expression in adipogenic and osteogenic, respectively

### CCR5 signaling in Macrophages


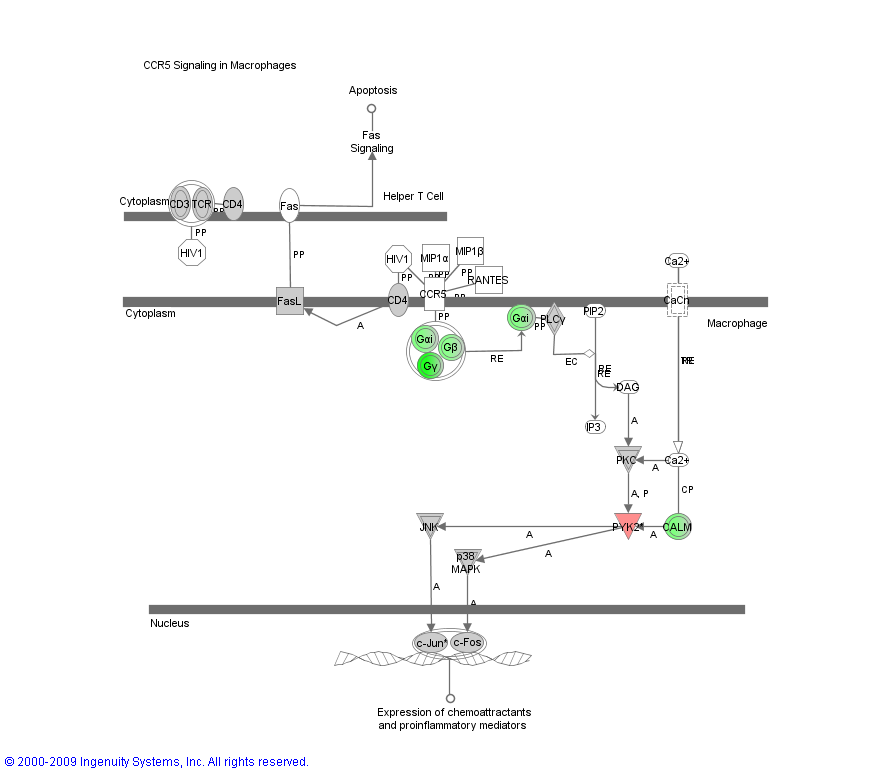


**OSTEOGENIC vs. ADIPOGENIC DIFFERENTIATION IN ASC**

**dd7**

Red and green shade in objects denote higher expression in adipogenic and osteogenic, respectively

### Relaxin signaling


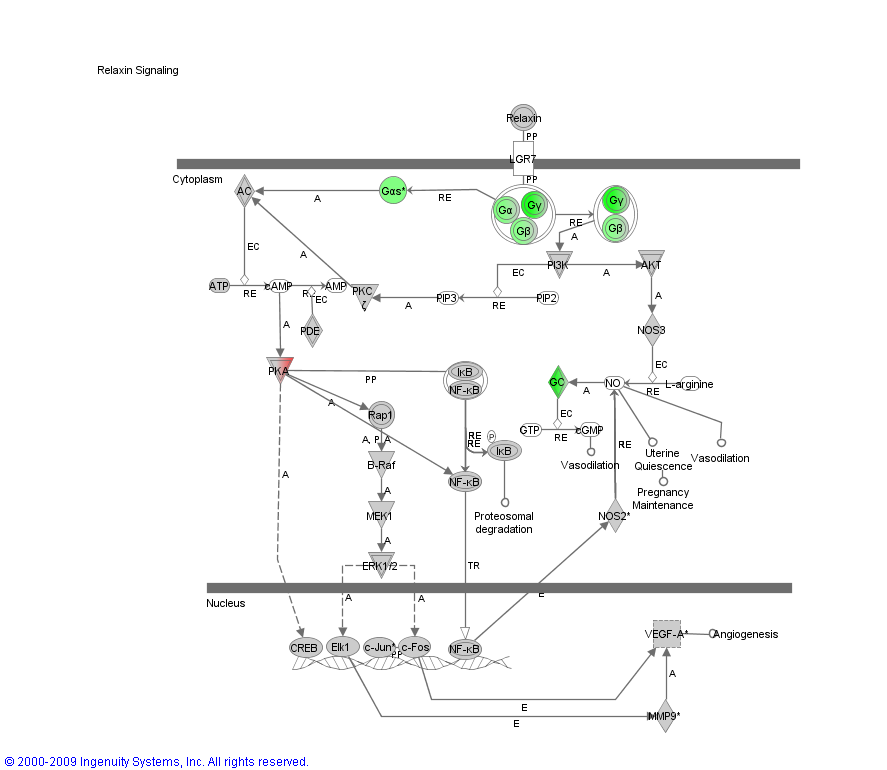


# OSTEOGENIC vs. ADIPOGENIC DIFFERENTIATION IN ASC

## dd21

### Overall statistic

| Pathways | -Log(P-value) | -Log(B-H P-value) | Ratio |
| --- | --- | --- | --- |
| CCR3 Signaling in Eosinophils | 4.41 | 2.23 | 0.09 |
| Chemokine Signaling | 4.04 | 2.16 | 0.11 |
| LPS/IL-1 Mediated Inhibition of RXR Function | 3.84 | 2.14 | 0.05 |
| Regulation of Actin-based Motility by Rho | 3.59 | 2.07 | 0.09 |
| Glutamate Receptor Signaling | 3.55 | 2.07 | 0.08 |
| CCR5 Signaling in Macrophages | 3.08 | 1.72 | 0.07 |
| Actin Cytoskeleton Signaling | 3.05 | 1.72 | 0.05 |
| fMLP Signaling in Neutrophils | 2.96 | 1.68 | 0.06 |
| Ephrin Receptor Signaling | 2.79 | 1.61 | 0.05 |
| Axonal Guidance Signaling | 2.68 | 1.56 | 0.04 |
| Fatty Acid Biosynthesis | 2.66 | 1.56 | 0.06 |
| α-Adrenergic Signaling | 2.49 | 1.42 | 0.07 |
| Role of NFAT in Regulation of the Immune Response | 2.15 | 1.14 | 0.04 |
| NRF2-mediated Oxidative Stress Response | 2.06 | 1.09 | 0.04 |
| Melatonin Signaling | 1.98 | 1.06 | 0.07 |
| IL-8 Signaling | 1.98 | 1.06 | 0.04 |
| Thrombin Signaling | 1.79 | 0.89 | 0.04 |
| Glycerophospholipid Metabolism | 1.71 | 0.83 | 0.03 |
| CXCR4 Signaling | 1.66 | 0.81 | 0.04 |
| Semaphorin Signaling in Neurons | 1.60 | 0.79 | 0.08 |
| Valine, Leucine and Isoleucine Degradation | 1.60 | 0.79 | 0.04 |
| Tight Junction Signaling | 1.58 | 0.79 | 0.04 |
| Virus Entry via Endocytic Pathways | 1.57 | 0.79 | 0.05 |
| LXR/RXR Activation | 1.53 | 0.77 | 0.05 |
| Cardiac β-adrenergic Signaling | 1.40 | 0.65 | 0.04 |
| Aryl Hydrocarbon Receptor Signaling | 1.33 | 0.59 | 0.04 |

**OSTEOGENIC vs. ADIPOGENIC DIFFERENTIATION IN ASC**

**dd21**

Red and green shade in objects denote higher expression in adipogenic and osteogenic, respectively

### Actin cytoskeleton signaling


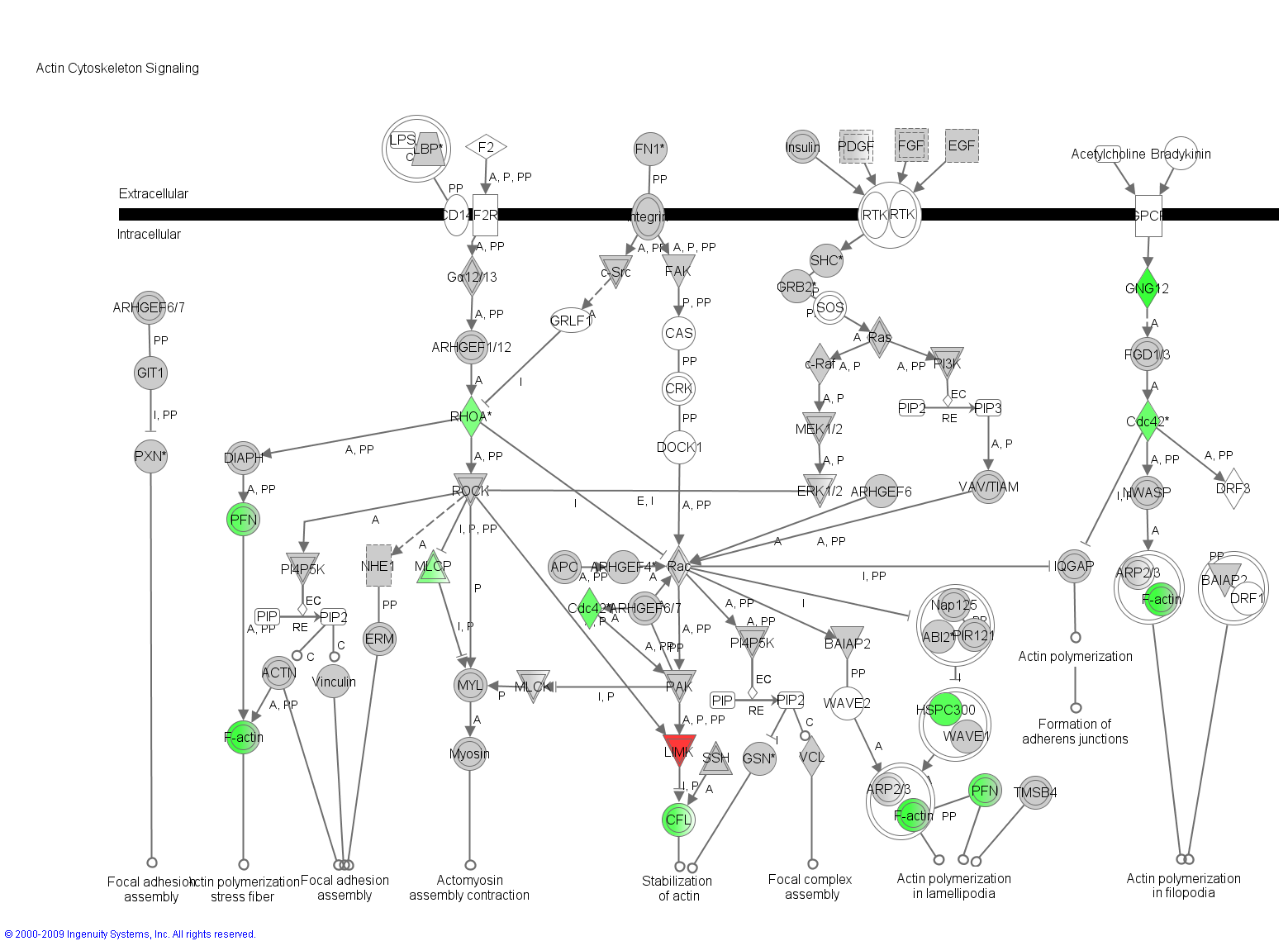


**OSTEOGENIC vs. ADIPOGENIC DIFFERENTIATION IN ASC**

**dd21**

Red and green shade in objects denote higher expression in adipogenic and osteogenic, respectively

### α adrenergic signaling


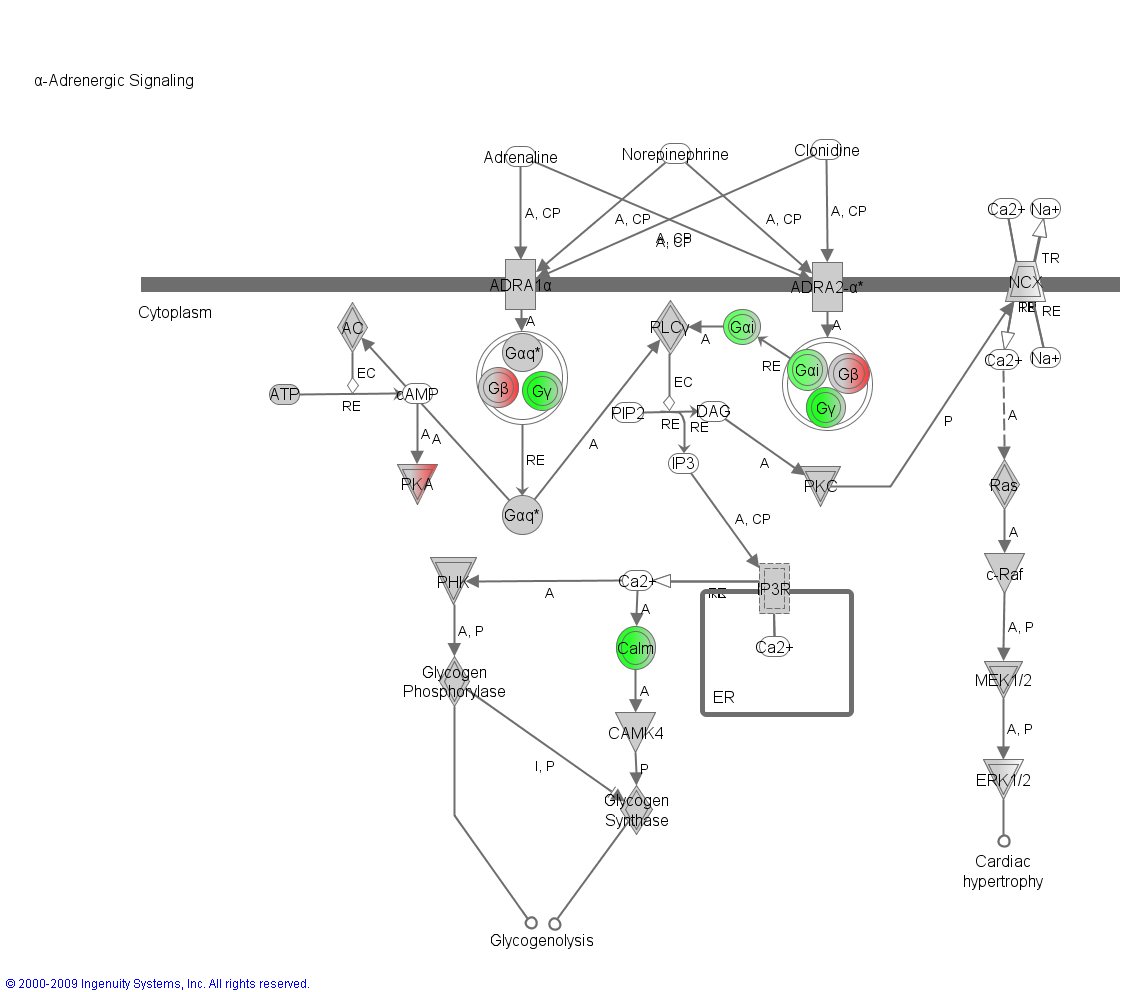


**OSTEOGENIC vs. ADIPOGENIC DIFFERENTIATION IN ASC**

**dd21**

Red and green shade in objects denote higher expression in adipogenic and osteogenic, respectively

### Axonal guidance signaling


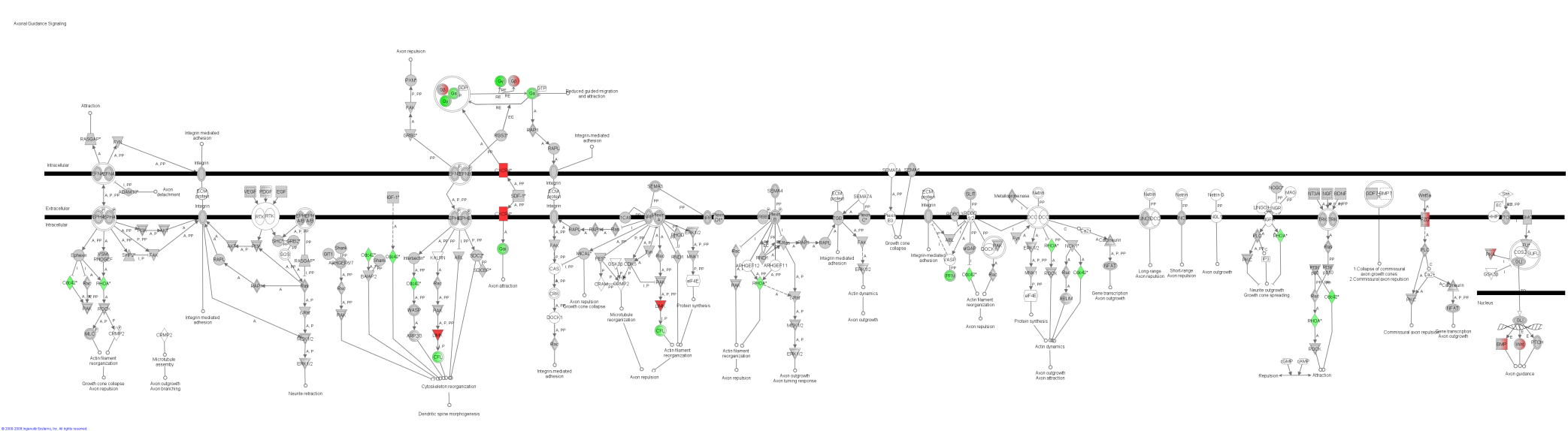


**OSTEOGENIC vs. ADIPOGENIC DIFFERENTIATION IN ASC**

**dd21**

Red and green shade in objects denote higher expression in adipogenic and osteogenic, respectively

### CCR3 signaling in Eosinophil


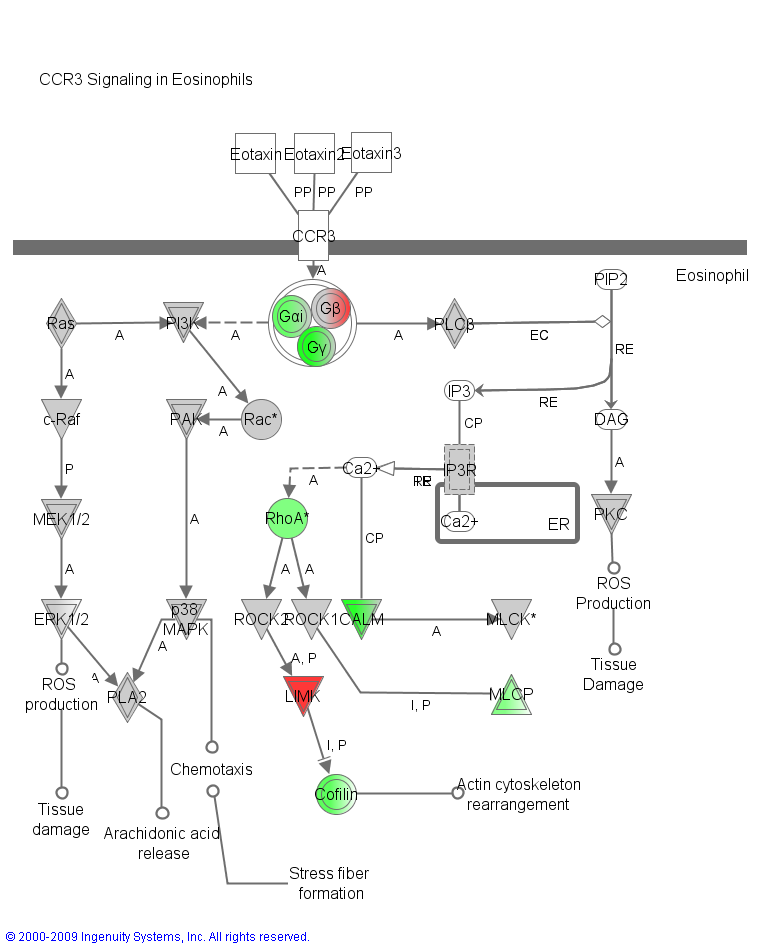


**OSTEOGENIC vs. ADIPOGENIC DIFFERENTIATION IN ASC**

**dd21**

Red and green shade in objects denote higher expression in adipogenic and osteogenic, respectively

### CCR5 signaling in Macrophages


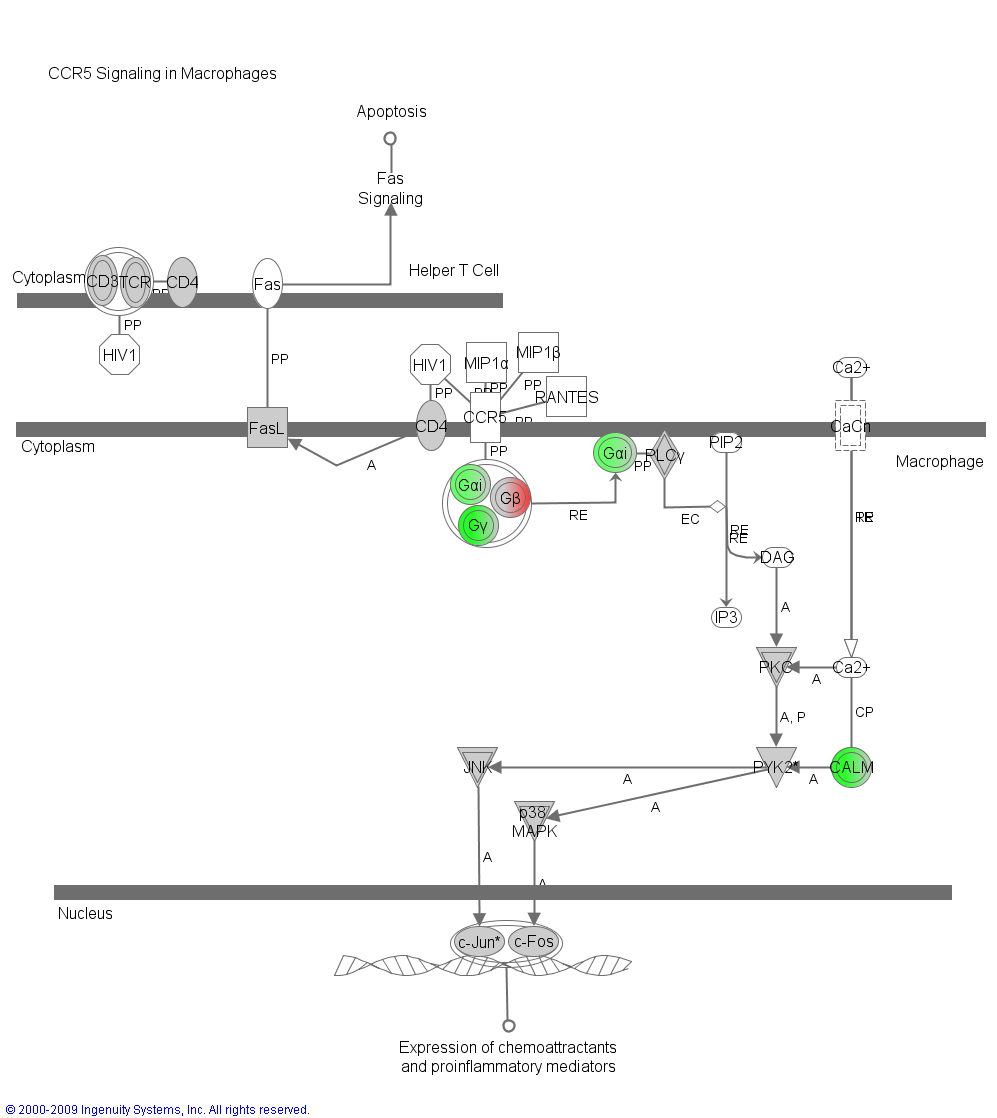


**OSTEOGENIC vs. ADIPOGENIC DIFFERENTIATION IN ASC**

**dd21**

Red and green shade in objects denote higher expression in adipogenic and osteogenic, respectively

### Chemokine signaling


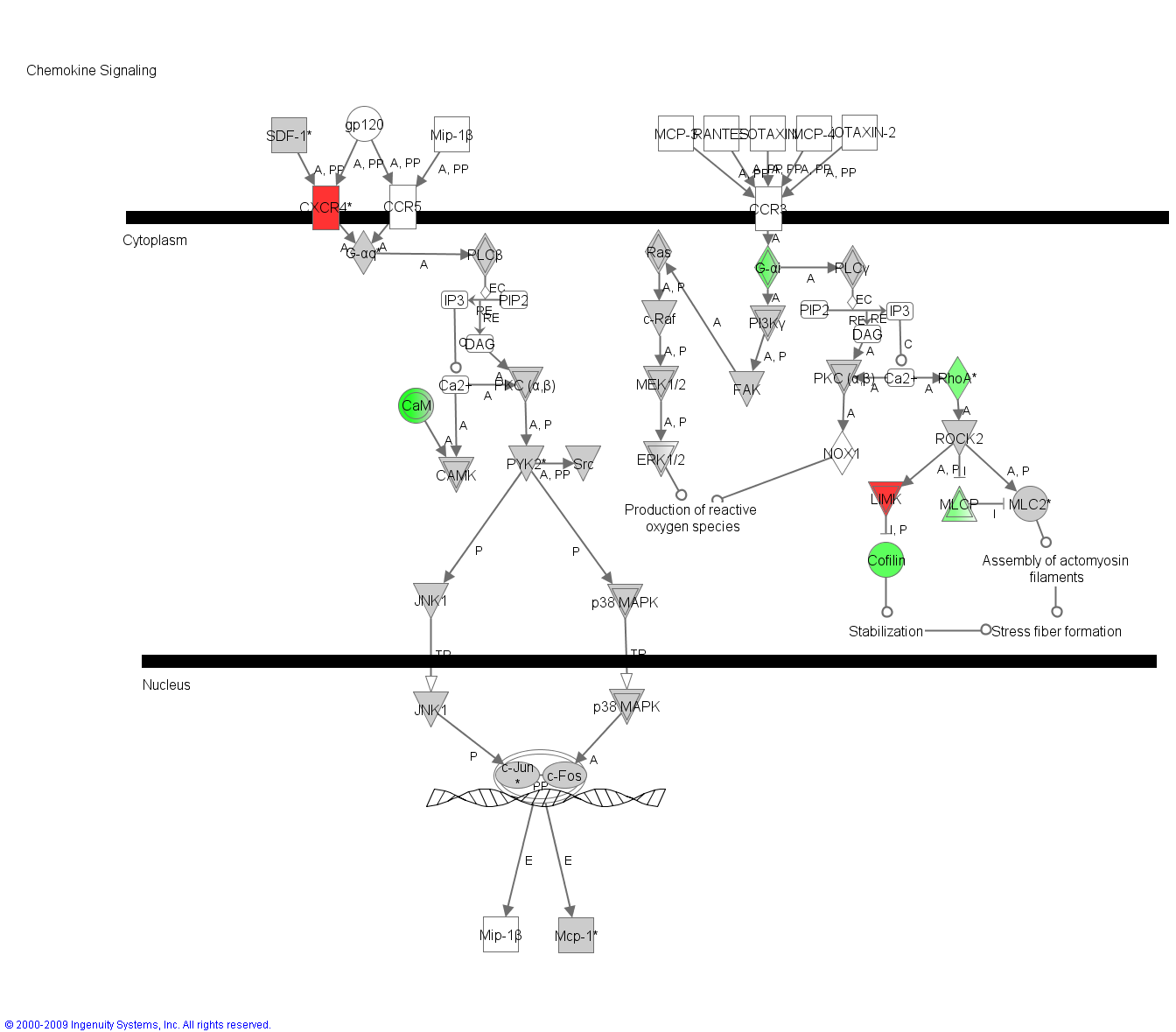


**OSTEOGENIC vs. ADIPOGENIC DIFFERENTIATION IN ASC**

**dd21**

Red and green shade in objects denote higher expression in adipogenic and osteogenic, respectively

### Ephrin receptor signaling


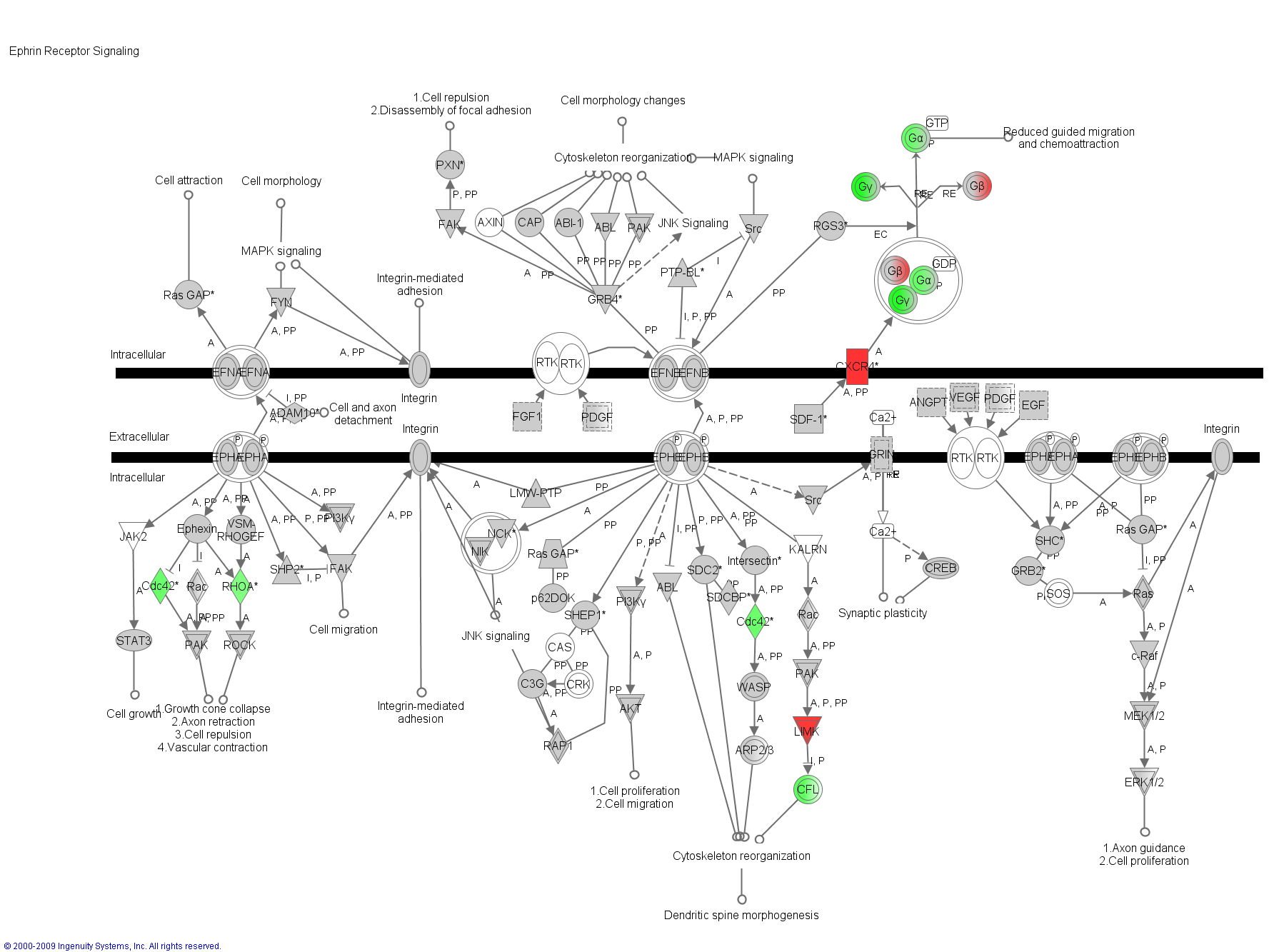


**OSTEOGENIC vs. ADIPOGENIC DIFFERENTIATION IN ASC**

**dd21**

Red and green shade in objects denote higher expression in adipogenic and osteogenic, respectively

### Fatty acid biosynthesis


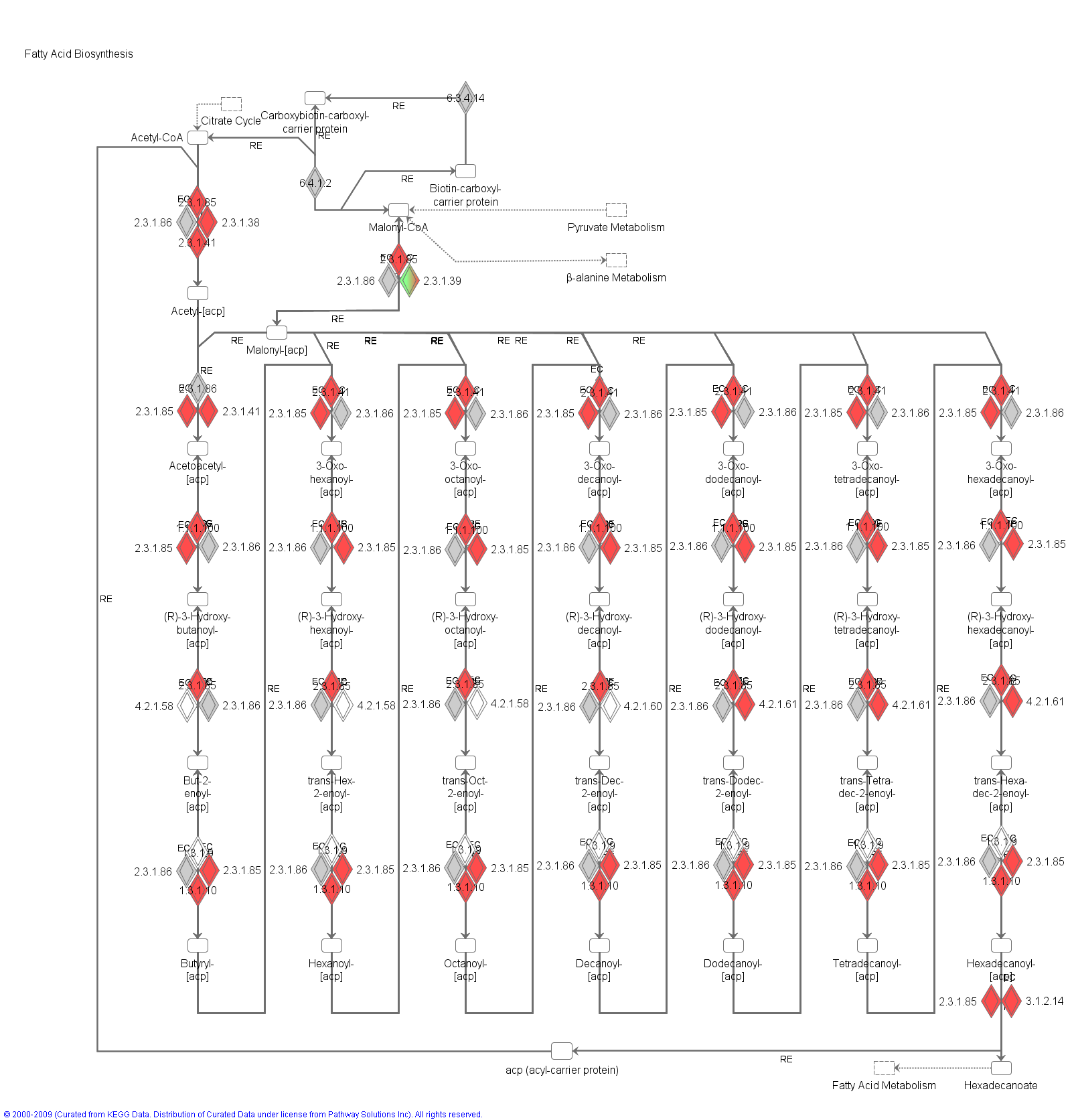


**OSTEOGENIC vs. ADIPOGENIC DIFFERENTIATION IN ASC**

**dd21**

Red and green shade in objects denote higher expression in adipogenic and osteogenic, respectively

### fMLP signaling in Neutrophils


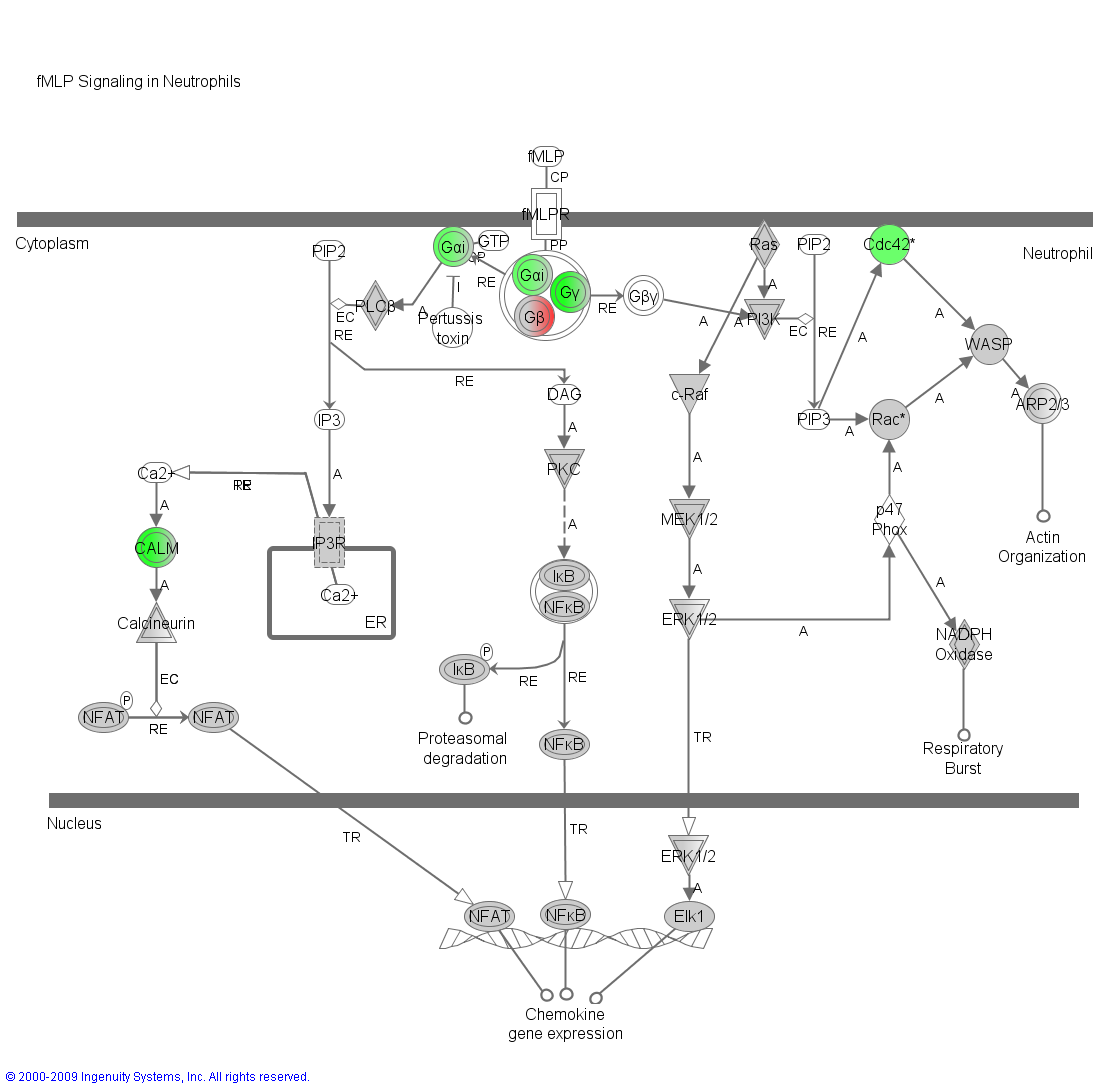


**OSTEOGENIC vs. ADIPOGENIC DIFFERENTIATION IN ASC**

**dd21**

Red and green shade in objects denote higher expression in adipogenic and osteogenic, respectively

### Glutamate receptor signaling


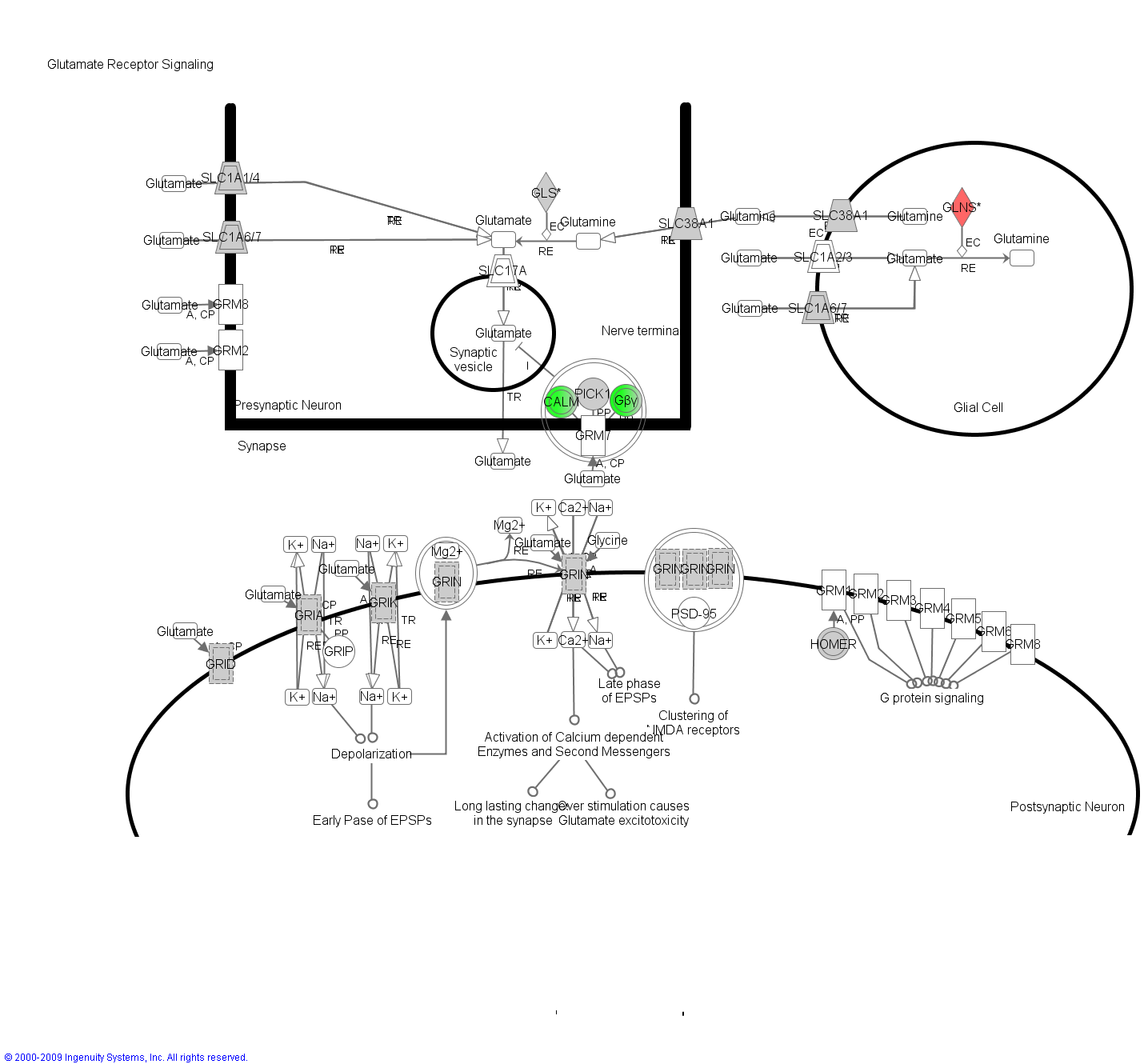


**OSTEOGENIC vs. ADIPOGENIC DIFFERENTIATION IN ASC**

**dd21**

Red and green shade in objects denote higher expression in adipogenic and osteogenic, respectively

### LPS/IL-1 Mediated Inhibition of RXR Function


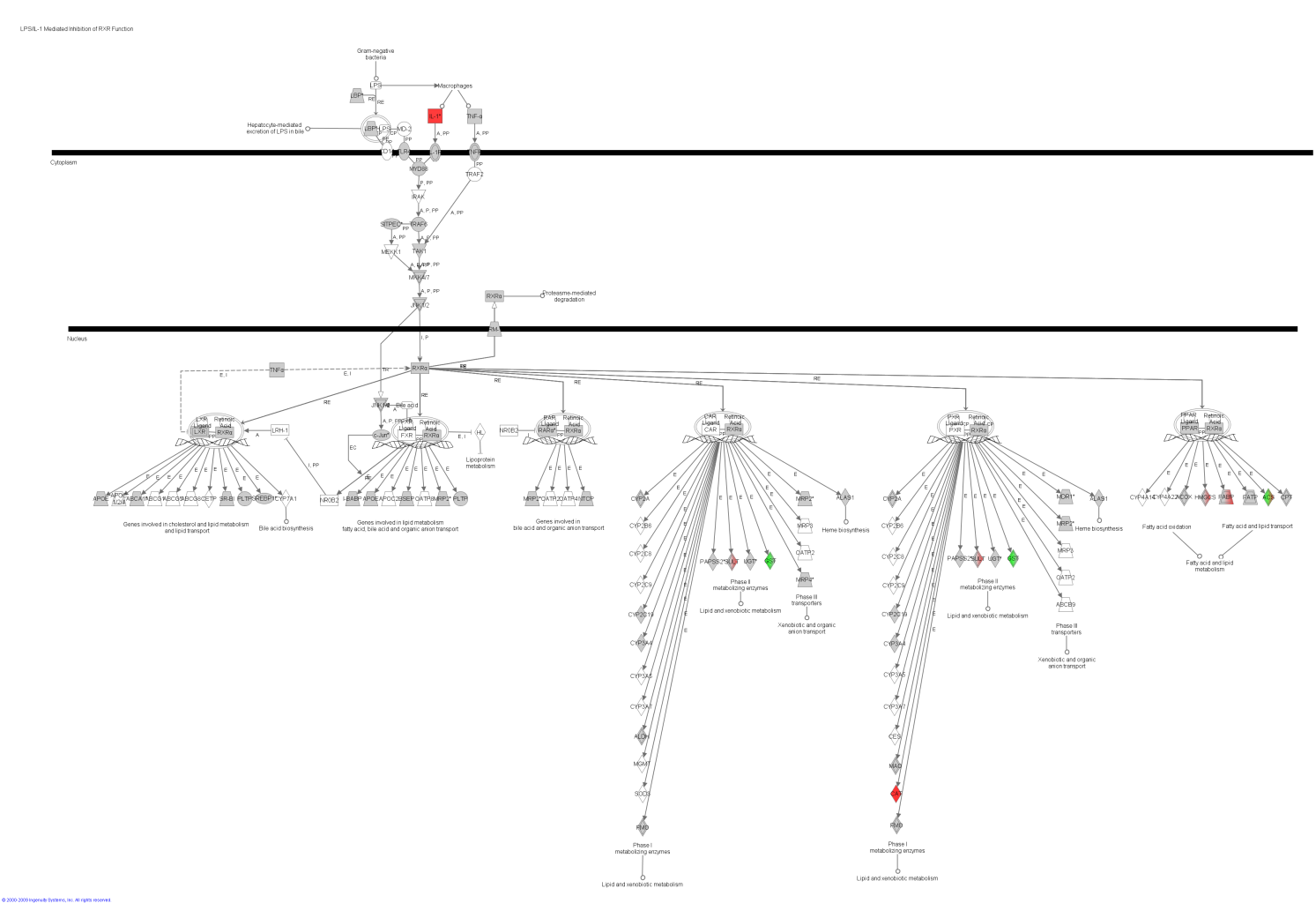


**OSTEOGENIC vs. ADIPOGENIC DIFFERENTIATION IN ASC**

**dd21**

Red and green shade in objects denote higher expression in adipogenic and osteogenic, respectively

### Regulation of Actin-based Motility by Rho


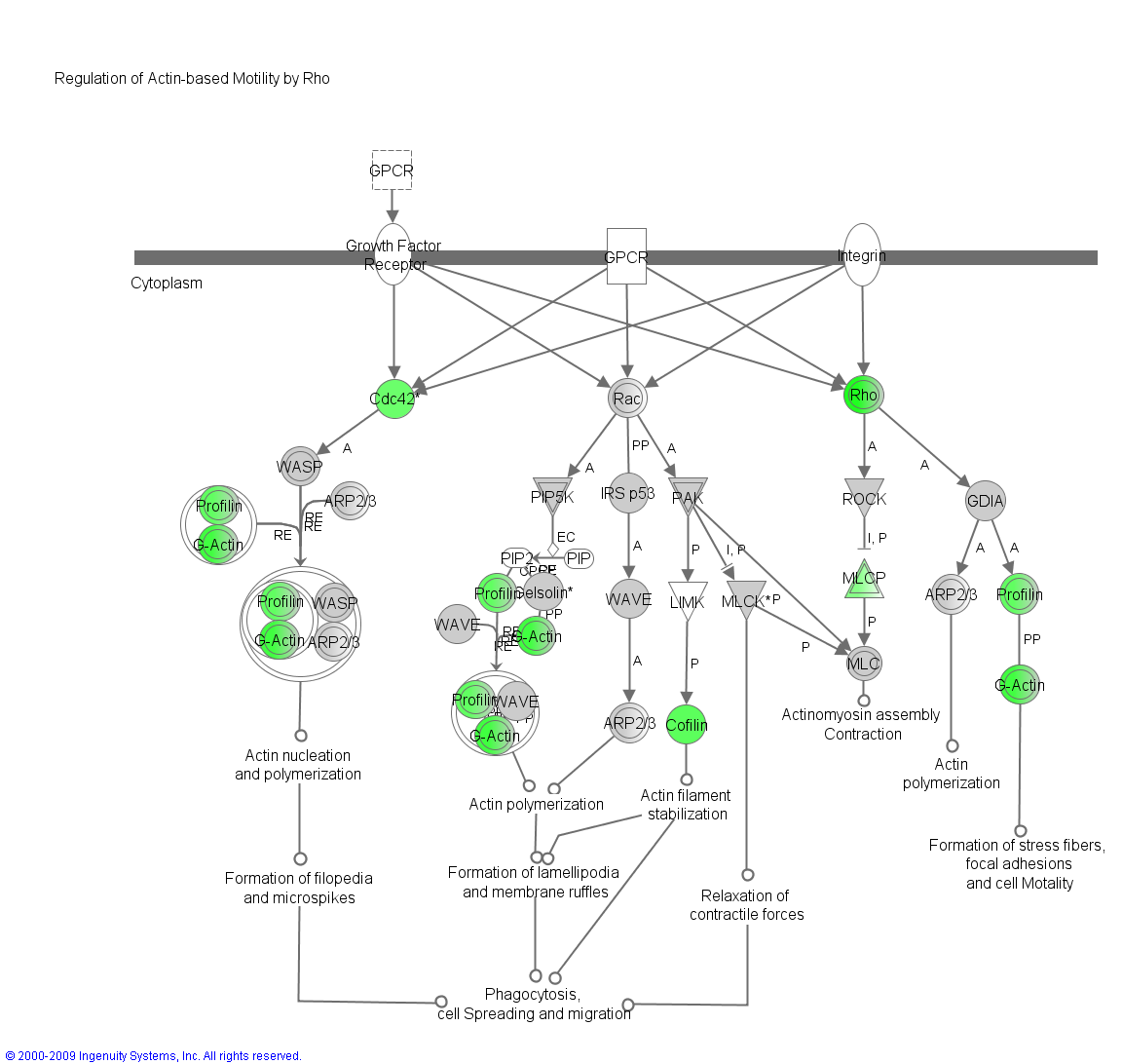


# OSTEOGENIC vs. ADIPOGENIC DIFFERENTIATION IN BMSC

## dd21

Red and green shade in objects denote higher expression in adipogenic and osteogenic, respectively

### LPS/IL-1 Mediated Inhibition of RXR Function


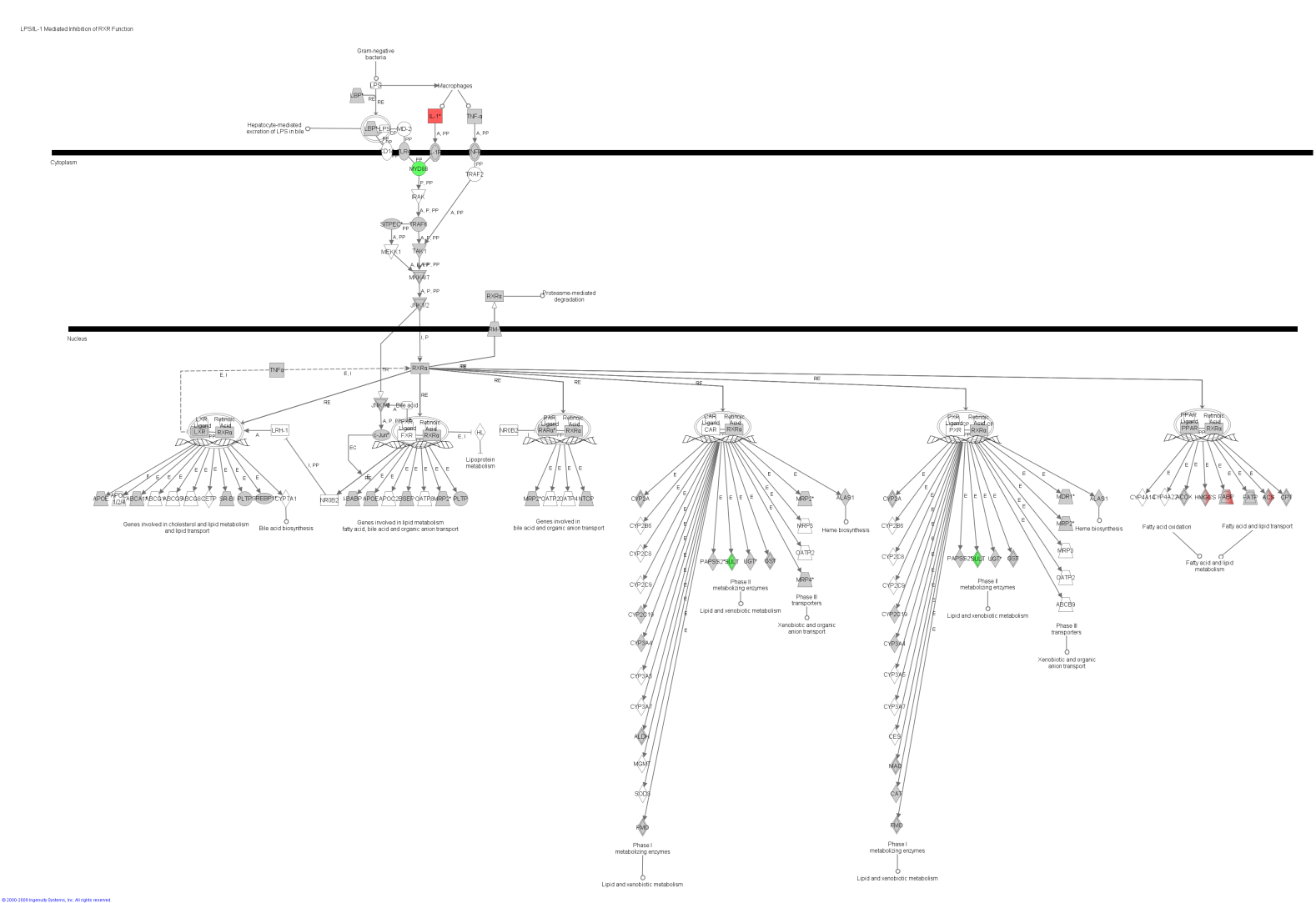


# BMSC vs. ASC DURING OSTEOGENIC DIFFERENTIATION

## dd2

Red and green shade in objects denote higher expression in BMSC and ASC, respectively

### 14-3-3 mediated signaling

**
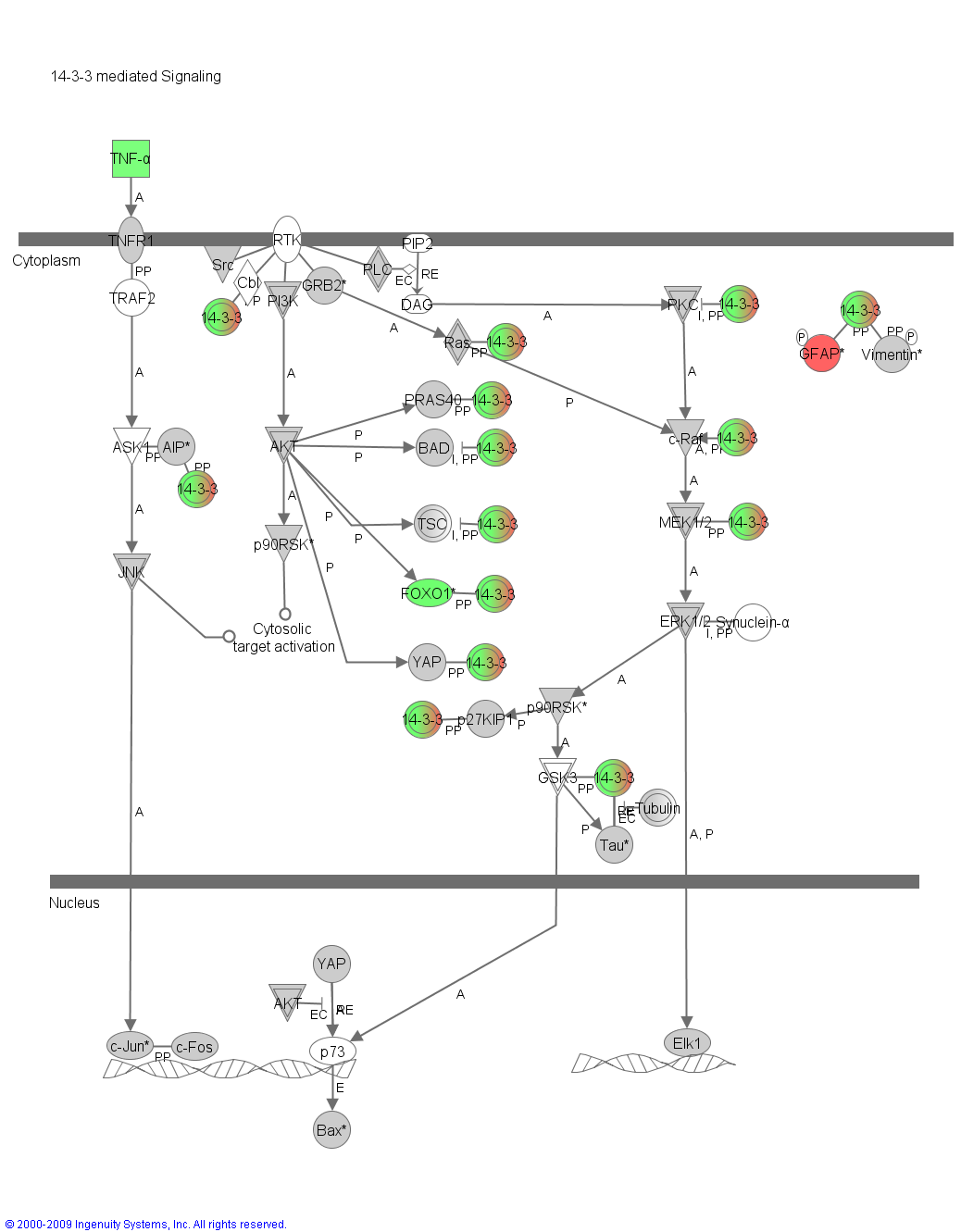
**

**BMSC vs. ASC DURING ADIPOGENIC DIFFERENTIATION**

## dd21

Red and green shade in objects denote higher expression in BMSC and ASC, respectively

### Keratan sulfate biosynthesis

**
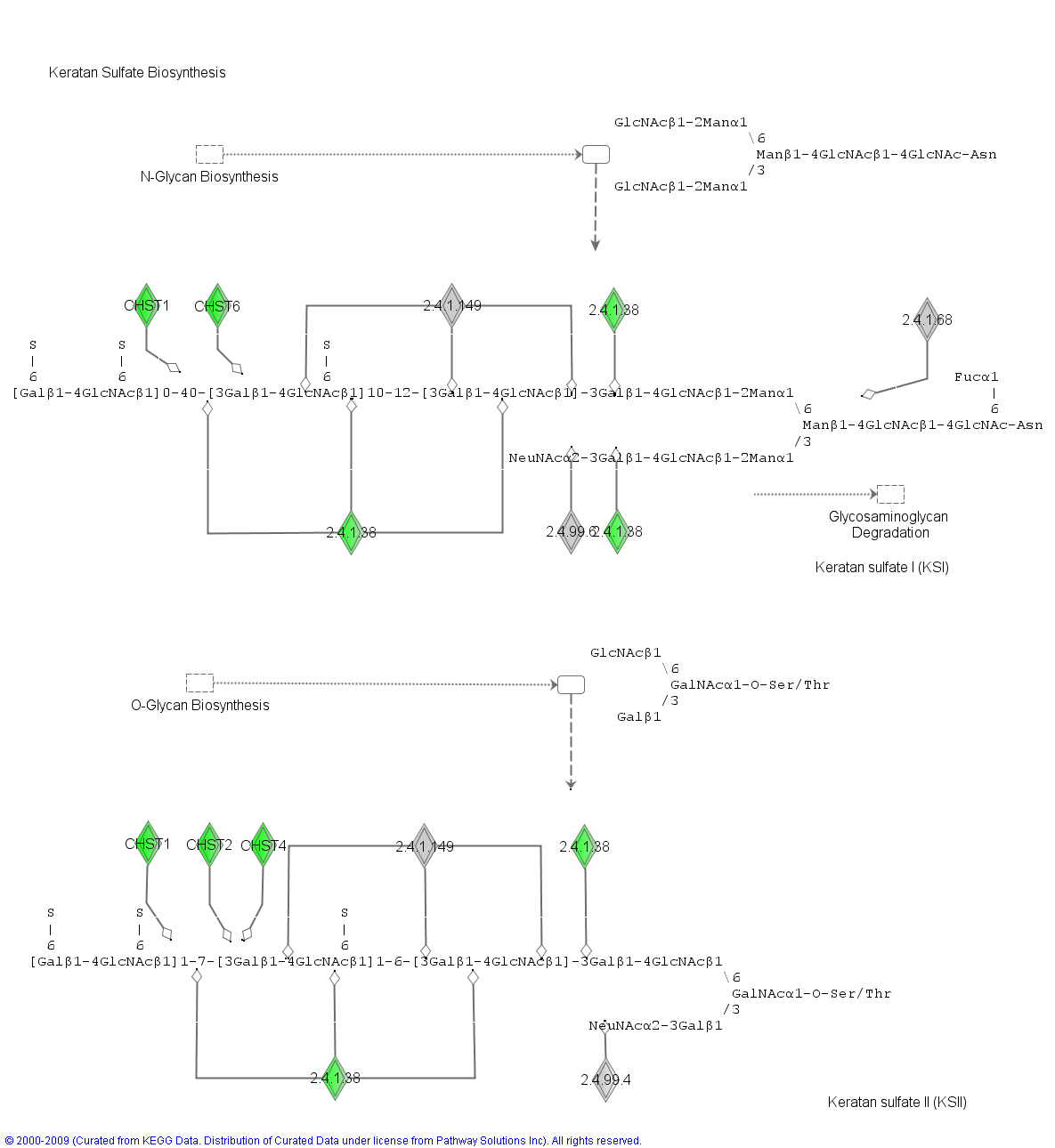
**

**BMSC vs. ASC DURING ADIPOGENIC DIFFERENTIATION**

**dd21**

Red and green shade in objects denote higher expression in BMSC and ASC, respectively

### LPS/IL-1 Mediated Inhibition of RXR Function


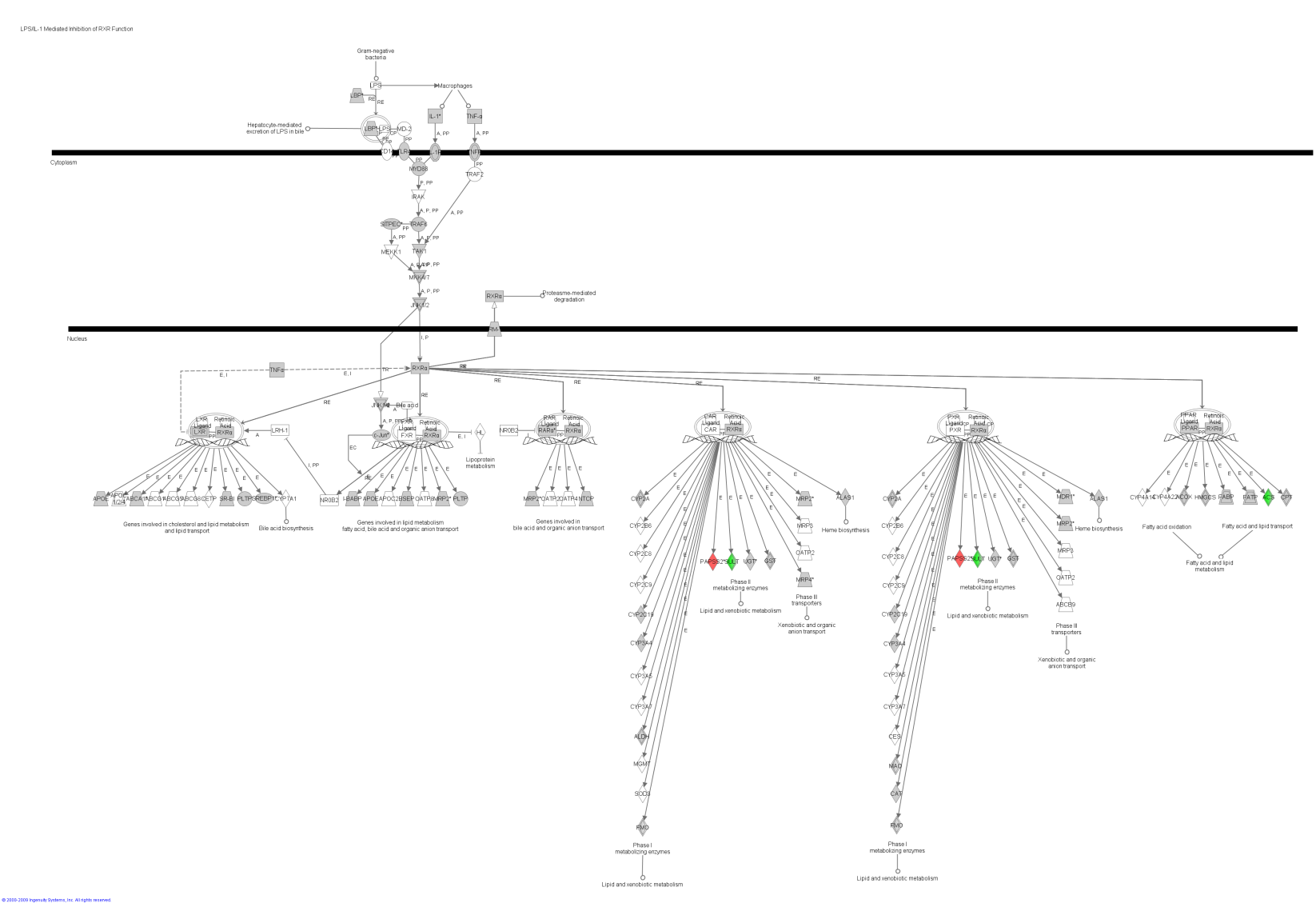


**BMSC vs. ASC DURING ADIPOGENIC DIFFERENTIATION**

**dd21**

Red and green shade in objects denote higher expression in BMSC and ASC, respectively

### Sulfur metabolism


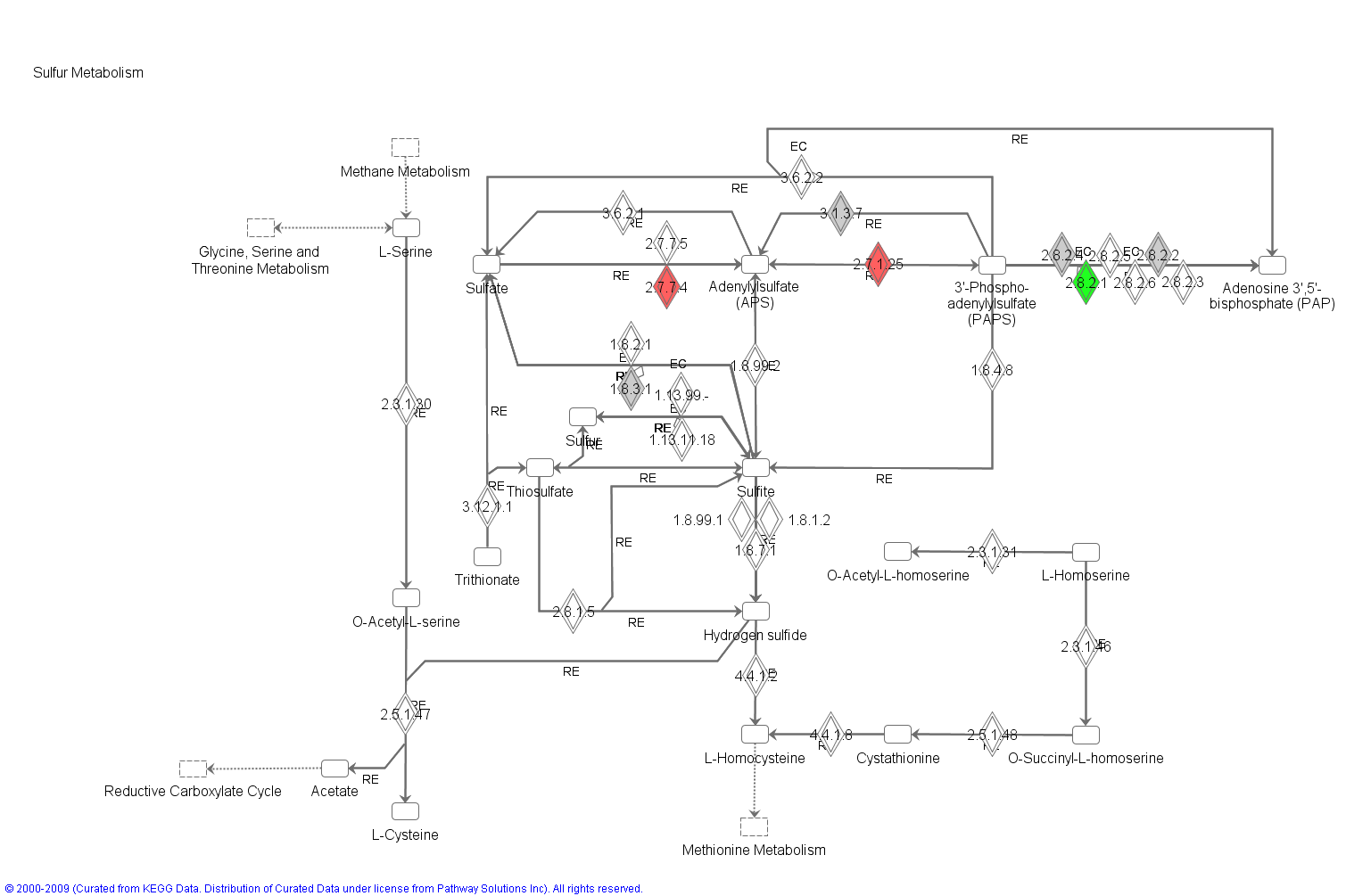


# NETWORK AMONG DEG

**(from ingenuity pathway analysis)**

## OSTEOGENIC vs. ADIPOGENIC DIFFERENTIATION IN ASC

### dd2

Red and green shade in objects denote higher expression in adipogenic and osteogenic, respectively

**
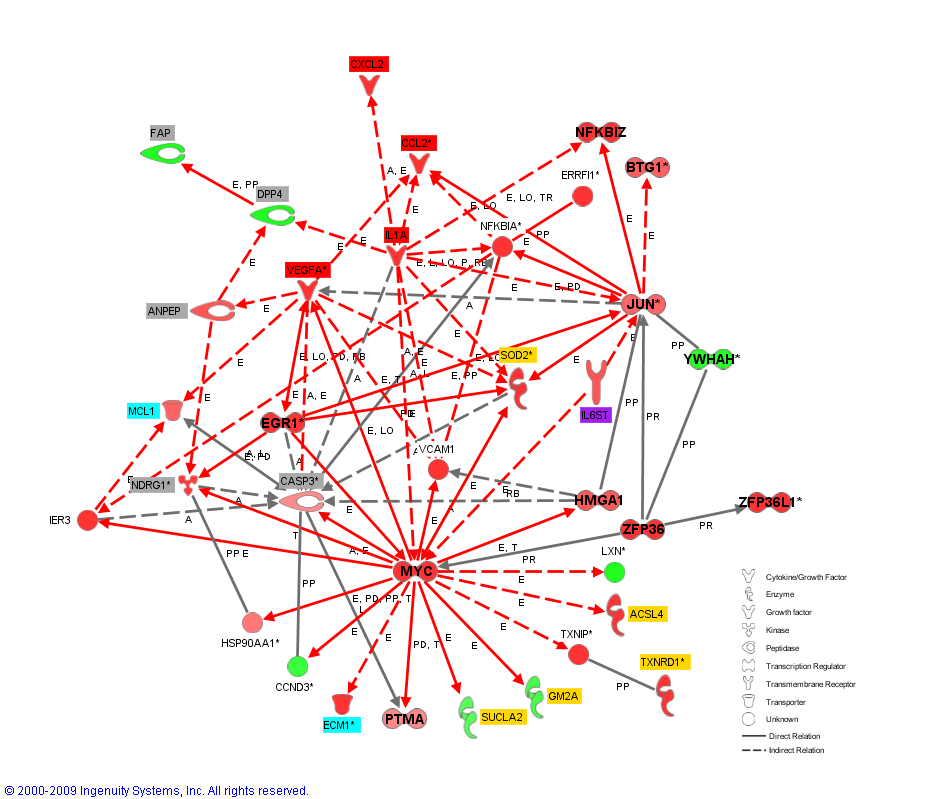
**

**OSTEOGENIC vs. ADIPOGENIC DIFFERENTIATION IN ASC**

### dd7

Red and green shade in objects denote higher expression in adipogenic and osteogenic, respectively


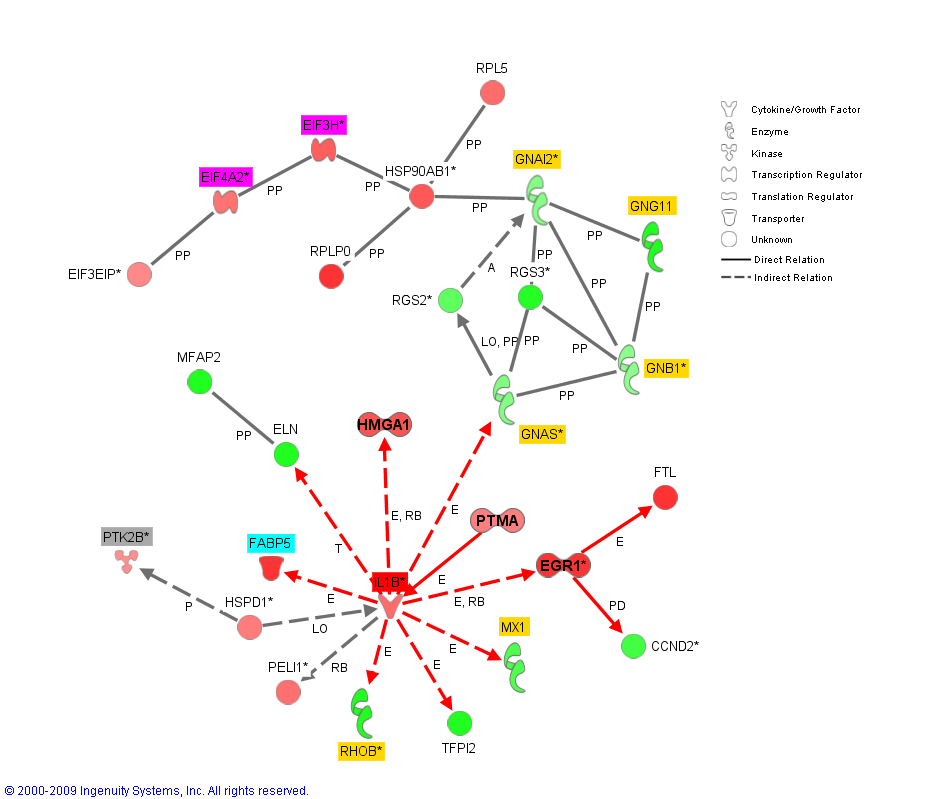


**OSTEOGENIC vs. ADIPOGENIC DIFFERENTIATION IN ASC**

### dd21

Red and green shade in objects denote higher expression in adipogenic and osteogenic, respectively


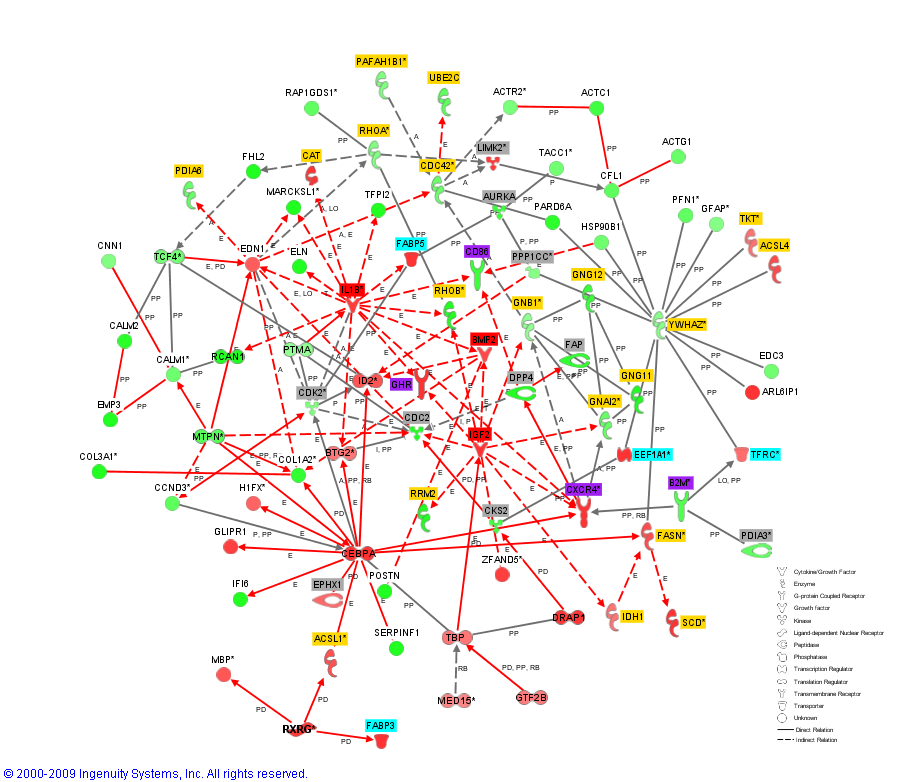


## OSTEOGENIC vs. ADIPOGENIC DIFFERENTIATION IN BMSC

### dd2

Red and green shade in objects denote higher expression in adipogenic and osteogenic, respectively

**
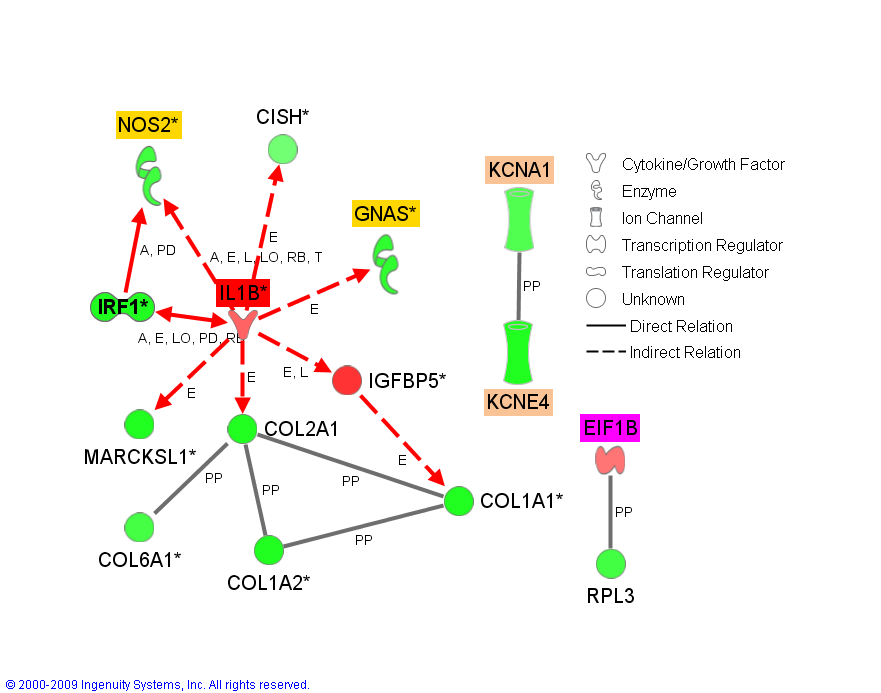
**

**OSTEOGENIC vs. ADIPOGENIC DIFFERENTIATION IN BMSC**

### dd7

Red and green shade in objects denote higher expression in adipogenic and osteogenic, respectively


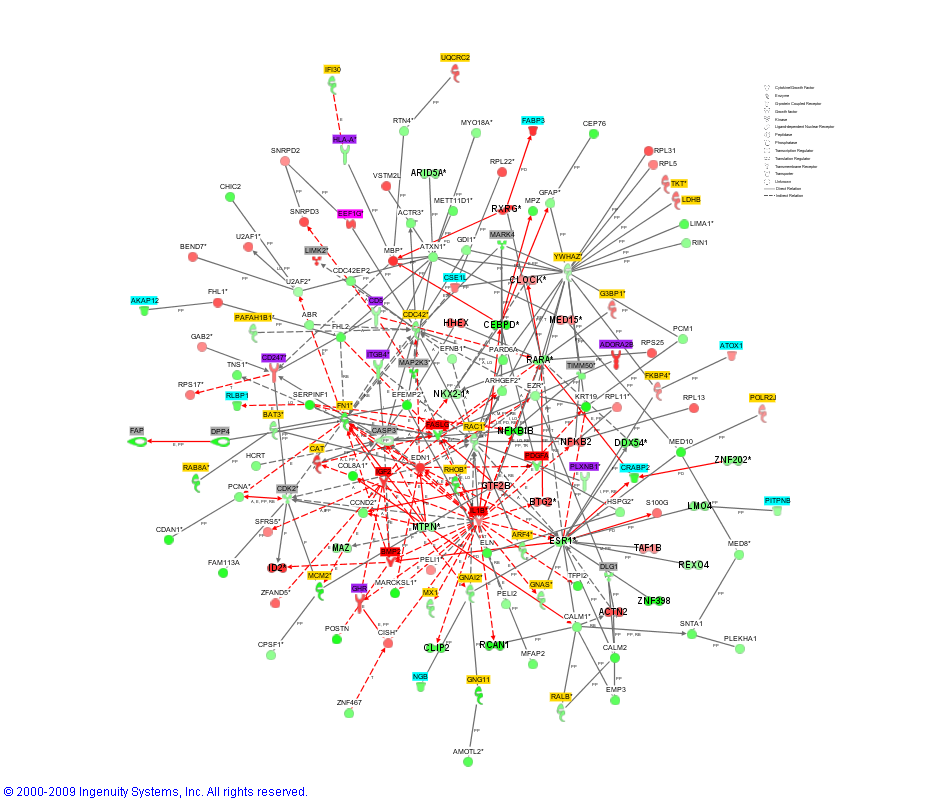


**OSTEOGENIC vs. ADIPOGENIC DIFFERENTIATION IN BMSC**

### dd21

Red and green shade in objects denote higher expression in adipogenic and osteogenic, respectively


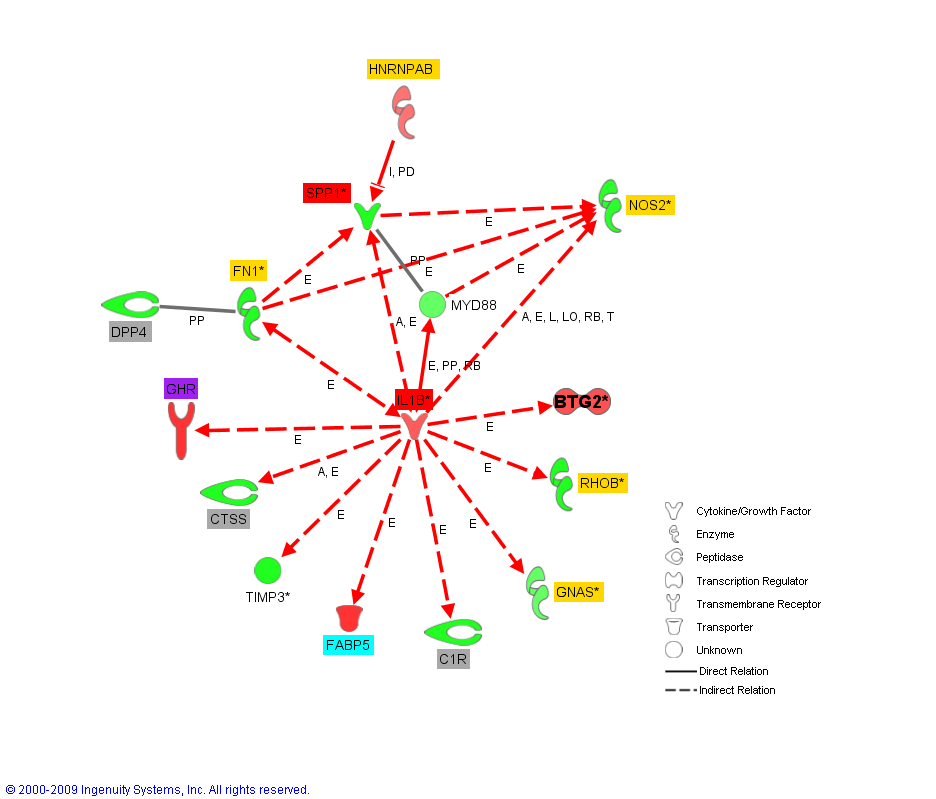


## BMSC vs. ASC DURING OSTEOGENIC DIFFERENTIATION

### dd2

Red and green shade in objects denote higher expression in BMSC and ASC, respectively


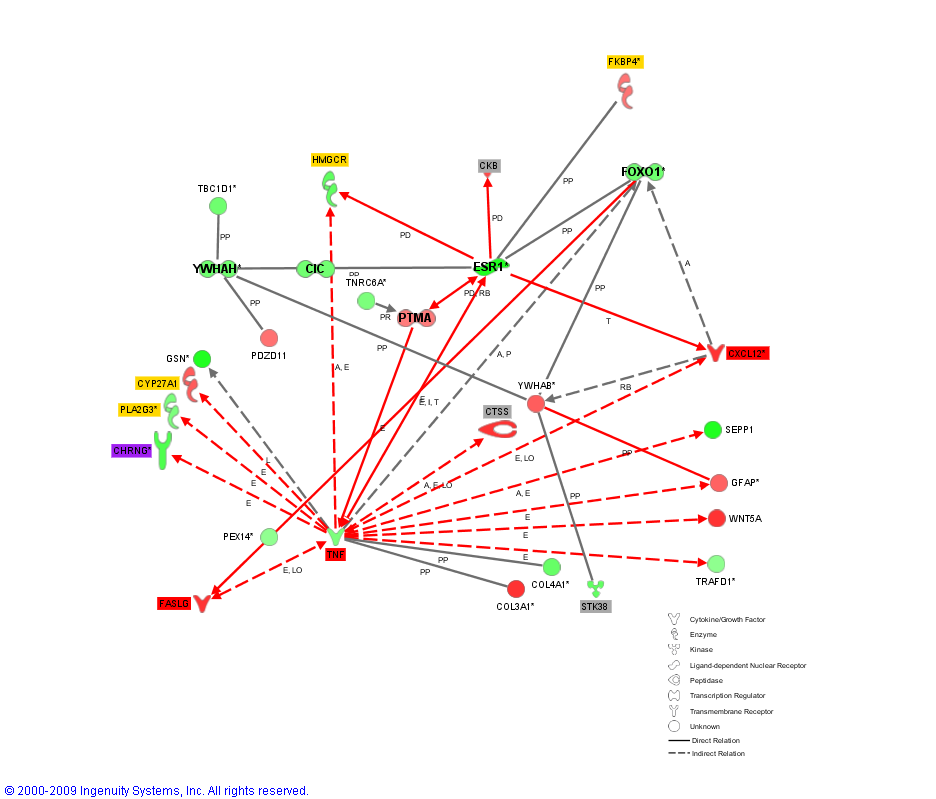


## BMSC vs. ASC DURING ADIPOGENIC DIFFERENTIATION

### dd2

Red and green shade in objects denote higher expression in BMSC and ASC, respectively


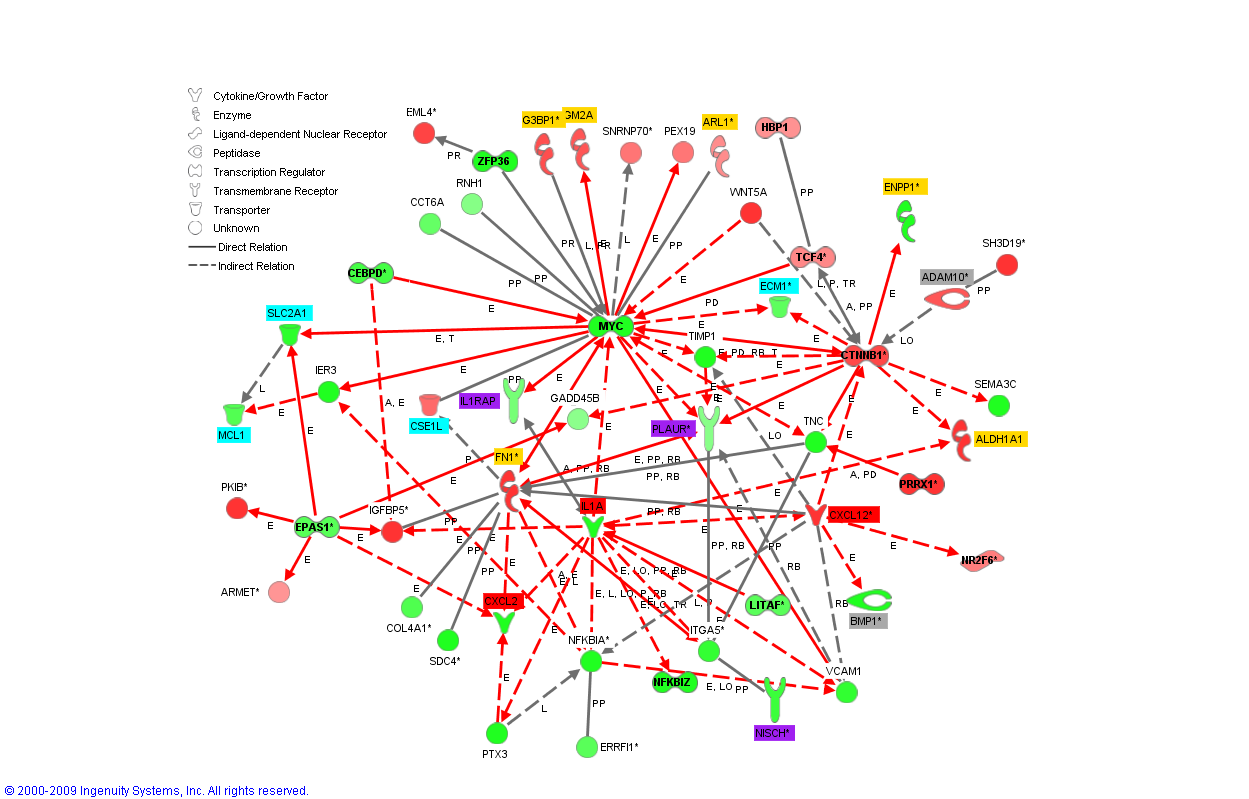


**BMSC vs. ASC DURING ADIPOGENIC DIFFERENTIATION**

### dd7

Red and green shade in objects denote higher expression in BMSC and ASC, respectively


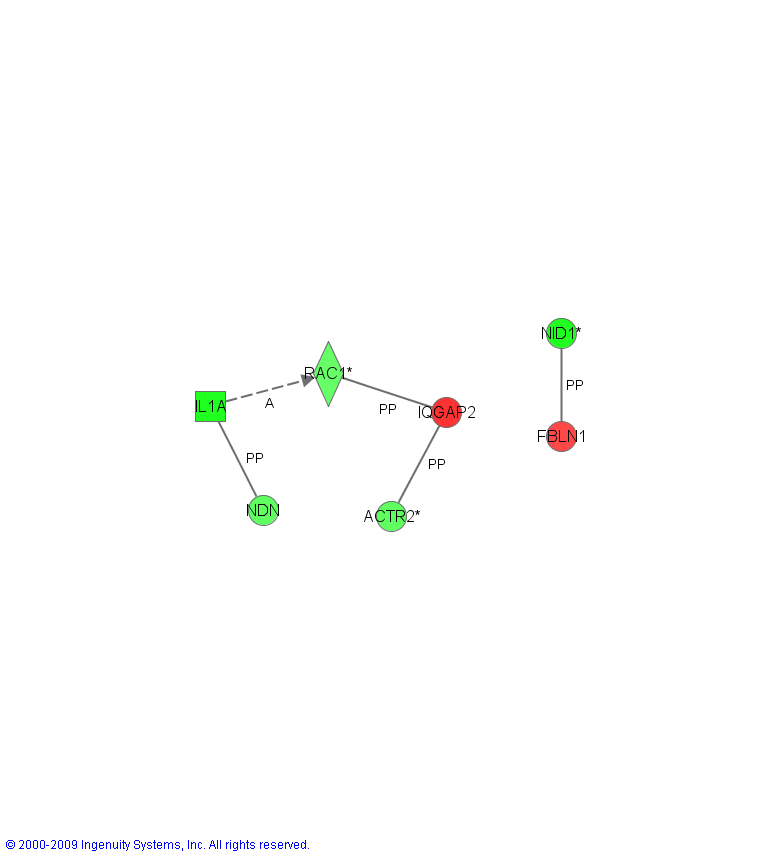

Supplement: File S7 — Results of significantly enriched pathway analysis from Ingenuity Pathway Analysis of DEG between adipogenesis and osteogenesis and DEG between BMSC and ASC during differentiation. The file contains the complete list of enriched pathways (P-value ≤0.05) and figures of the enriched pathways for each comparison. (DOCX) [file pone.0032481.s012.docx]
