# Supplementary material for: Transcriptomics Comparison between Porcine Adipose and Bone Marrow Mesenchymal Stem Cells during In Vitro Osteogenic and Adipogenic Differentiation
Source: PLoS One. 2012 Mar 7;7(3):e32481. doi: 10.1371/journal.pone.0032481 (PMC3296722; doi:10.1371/journal.pone.0032481)
Supplement: Table S1 — Pearson correlation between ASC and BMSC transcriptome. Reported are the results from SAS analysis of overall Pearson correlation between ASC and BMSC transcriptome overall, before differentiation (dd0) for all the pigs and for each single pig; between differentiations in the same cell type (ASC or BMSC) overall and for each single pig; and overall correlation between ASC and BMSC during adipogenic and osteogenic differentiation. All correlations were significant at p<0.0001. (DOCX) [file pone.0032481.s017.docx]

Table S1

|  |  |  |
| --- | --- | --- |
| **ASC *vs.* BMSC** |  |  |
| Overall | 0.78 |  |
|  | dd0 |  |
| All pigs | 0.79 |  |
| Pig 12 | 0.90 |  |
| Pig 22 | 0.70 |  |
| Pig 40 | 0.85 |  |
| **Osteogenic *vs.* adipogenic** |  |  |
| Overall | 0.78 |  |
|  | Cell type | |
|  | ASC | BMSC |
| Overall | 0.80 | 0.74 |
| dd2 | 0.81 | 0.78 |
| dd7 | 0.79 | 0.67 |
| dd21 | 0.81 | 0.83 |
| Pig 12 | 0.81 | 0.86 |
| Pig 22 | 0.83 | 0.63 |
| Pig 40 | 0.79 | 0.79 |
| **ASC *vs.* BMSC** |  |  |
| Overall | 0.78 |  |
|  | Differentiation | |
|  | Osteogenic | Adipogenic |
|  |  |  |
| Whole differentiation | 0.79 | 0.76 |
| dd2 | 0.83 | 0.75 |
| dd7 | 0.72 | 0.72 |
| dd21 | 0.83 | 0.87 |
