# Supplementary material for: Transcriptomics Comparison between Porcine Adipose and Bone Marrow Mesenchymal Stem Cells during In Vitro Osteogenic and Adipogenic Differentiation
Source: PLoS One. 2012 Mar 7;7(3):e32481. doi: 10.1371/journal.pone.0032481 (PMC3296722; doi:10.1371/journal.pone.0032481)
Supplement: Table S2 — Functional analysis results by IPA of adipogenic and osteogenic differentiation of ASC at dd2. Tabulated results from Ingenuity Pathway Analysis® (IPA) effect on function analysis of DEG between adipogenic and osteogenic differentiation of ASC at dd2. Reported are the functions sorted by decrease in significance. The category denotes the main functional category assigned by IPA. The function annotation is derived by the “effect on function” in IPA. In parenthesis are reported the number of DEG for each specific function and the arrows denote the overall effect on the function inferred by the gene annotation using IPA (⇑⇑ = highly activated in adipogenic vs. osteogenic differentiation; ⇑ = activated in adipogenic vs. osteogenic differentiation; ↑ = tends to be activated in adipogenic vs. osteogenic differentiation; ⇓⇓ = highly activated in osteogenic vs. adipogenic differentiation; ⇓ = activated in osteogenic vs. adipogenic differentiation; ↓ = tends to be activated in osteogenic vs. adipogenic differentiation) following the criteria reported in Materials and Methods in file S1. In yellow shade all functions enriched with a B–H FDR≤0.05. (DOCX) [file pone.0032481.s018.docx]

**Table S2**

| **Category** | **Function Annotation** | **DEG** |  |
| --- | --- | --- | --- |
| Cell death | Cell death (38, **⇑**), cell death of normal cells (22, **⇑**), tumor cell lines (21, **↓**), bone marrow cells (6, ⇔); apoptosis (36, **⇑**), apoptosis of normal cells (22, **⇑**), tumor cell lines (17, **↓**), leukocytes (13, ⇔), T lymphocytes (7, **↑**), connective tissue cells (6, **⇑**); survival of cells (18, **↑**), tumor cell lines (9, ⇔). | 41 **⇑** |  |
| Cellular Movement | | Migration of cells (17, ⇔), eukaryotic cells (15, **↑**), leukocytes (8, **↑**), phagocytes (5, **⇑**), bone marrow cells (4, **⇑**); cell movement of normal cells (13, **↑**), blood cells (10, **⇑**), leukocytes (9, **⇑**), phagocytes (6, **⇑⇑**); chemotaxis of leukocytes (6, **↑**); homing of bone marrow cells (4, **⇑⇑**). | 22 **↑** |
| Hematologycal System Development & Function | | Proliferation of leukocytes (12, **⇑**), lymphocytes (11, **⇑**), T lymphocytes (8, **↑**); differentiation of blood cells (10, **↑**), leukocytes (9, **↑**); quantity of leukocytes (10, **⇑**), mononuclear leukocytes (8, **↑**), lymphocytes (7, ⇔). | 27 **↑** |
| Cellular Development | | Differentiation of cells (23, **↑**), blood cells (10, **↑**), bone marrow cells (7, **↓**), osteocytes (7, **↑**), osteoclasts (6, **↑**), embryonic stem cells (4, ⇔); development of cells (17, **⇑**), endothelial cells (4, **⇑**); developmental process of blood cells (14, ⇔), bone marrow cells (10, **↓**), stem cells (5, ⇔); morphogenesis of cells (10, **⇑**). | 31 **↑** |
| Cellular Growth & Proliferation | | Proliferation of cells (28, **↑**); growth of cells (27, **↑**), normal cells (22, **↑**), tumor cell lines (13, **↓**), blood cells (7, ⇔); colony formation of eukaryotic cells (8, **↑**), osteoclasts (3, **⇑**). | 36 **↑** |
| Cell Cycle | | Cell division process of cells (16, **↑**); cell stage of cells (14, **↑**); arrest in cells division process of eukaryotic cells (9, **↑**); mitosis (7, **↑**); entry into cell division process of cells (6, **↑**); remodeling of chromatin (5, **↑**); senescence of eukaryotic cells (5, **↑**); delay in cell division process of cell lines (4, **⇑**); arrest in cells division process of cancer cells (3, **↑**). | 18 **↑** |
| Connective Tissue Development & Function | Differentiation of osteocytes (7, **⇑**), osteoclasts (6, **⇑**); growth of fibroblast cell lines (6, **↑**); quantity of osteoclasts (5, **↑**). | 19 **⇑** |  |
| Tissue Morphology | | Quantity of cells (17, **↑**), leukocytes (10, **⇑**), mononuclear leukocytes (7, **↑**), connective tissue cells (6, **↑**), lymphocytes (7, **↑**). | 18 **↑** |
| Gene Expression | | Activation of gene (7, **⇑**), NFkB binding site (5, **↑**), protein binding site (4, **⇑**); binding of DNA (7, ⇔), NFkB binding site (5, **↓**). | 16 **↑** |
| RNA Damage & Repair | | Stabilization of mRNA (4, **↑**). | 4 **↑** |
| RNA Post Transcriptional Modification | | Stabilization of mRNA (4, **↑**). | 4 **↑** |
| Antigen Presentation | | Inflammatory response (9, **↑**); inflammation (6, ⇔); accumulation of macrophages (4, **⇑**), peripheral blood monocytes (2, **↑**); binding of monocytes (3, **↑**). | 13 **↑** |
| Immune Cell Trafficking | | Cell movement of leukocytes (9, **⇑**), granulocytes (6, **⇑⇑**), phagocytes (6, **⇑⇑**); migration of leukocytes (8, **↑**), bone marrow cells (4, **⇑**); adhesion of leukocytes (7, **⇑**), mononuclear leukocytes (4, **⇑**); accumulation of leukocytes (6, **⇑⇑**); chemotaxis of leukocytes (6, **↑**); homing of bone marrow cells (4, **⇑**). | 13 **⇑** |
| Tissue Development | | Adhesion of eukaryotic cells (10, **⇑**), endothelial cells (4, **⇑⇑**); accumulation of cells (7, **⇑⇑**), leukocytes (6, **⇑⇑**), granulocytes (3, **⇑⇑**), mononuclear leukocytes (3, **⇑⇑**), macrophages (2, **⇑**). | 16 **⇑⇑** |
| Molecular Transport | | Quantity of reactive oxygen species (4, **↑**); release of protein (4, **↑**); clearance of D-glucose (2, **⇑**). | 8 **↑** |
| Protein Synthesis | | Release of protein (4, **↑**). | 4 **↑** |
| Cardiovascular System Development & Function | | Development of blood vessel (10, **⇑**); adhesion of endothelial cells (4, **⇑⇑**); angiogenesis of blood vessel (4, **⇑⇑**). | 13 **⇑** |
| Cell Morphology | | Morphogenesis of cells (10, **⇑**); transformation of eukaryotic cells (9, ⇔); branching of eukaryotic cells (3, **↑**); permeabilization of mitochondria (3, **↑**). | 26 **↑** |
| Cell-To-Cell Signaling & Interaction | | Adhesion of eukaryotic cells (10, **⇑**), leukocytes (7, **⇑⇑**), endothelial cells (4, **⇑⇑**), mononuclear leukocytes (4, **⇑⇑**); recruitment of normal cells (5, **⇑⇑**), granulocytes (4, **⇑⇑**); stimulation of eukaryotic cells (5, **⇑⇑**); activation of granulocytes (3, **⇑⇑**). | 23 **⇑⇑** |
| Tumor Morphology | | Formation of tumor (6, **⇑**), malignant tumor (4, **⇑**); development of tumor (5, **⇑**). | 12 **⇑** |
| Hematopoiesis | | Differentiation of leukocytes (9, **↑**); bone marrow cells (7, ⇔); growth of hematopoietic progenitor cells (4, **⇑**); homing of bone marrow cells (4, **⇑**); migration of bone marrow cells (4, **⇑**). | 18 **⇑** |
| Cellular Assembly & Organization | | Remodeling of chromatin (5, **↑**); quantity of focal adhesion (3, **↓**). | 12 ⇔ |
| DNA Replication, Recombination & Repair | | Synthesis of DNA (9, **⇑**); metabolism of DNA (8, **↑**); damage of DNA (4, ⇔); breakage of chromosomes (2, ⇔). | 18 **↑** |
| Post-Transcriptional Modification | | Association of protein (2, ⇔); hydroxylation of protein fragment (1, **↑**). | 3 ⇔ |
| Carbohydrate Metabolism | | Removal of D-glucose (3, **↑**); clearance of D-glucose (2, **⇑**); hydrolysis of phosphatidylcholine (2, **↓**). | 7 **↑** |
| Small Molecule Biochemistry | | Hydrolysis of lipid (5, **⇓⇓**), triacylglycerol (2, **⇓)**; synthesis of nitric oxide (4, **⇑⇑**); generation of prostaglandin (3, **⇑**). | 18 ⇔ |
| Cell Signaling | | Degeneration of cells (4, ⇔); breakage of chromatin (1, **↓**). | 7 ⇔ |
| Nucleic Acid Metabolism | | Deamination of dCMP (1, **↓**); metabolism of UDP-N- acetylglucosamine (2, ⇔). | 3 ⇔ |
| Lipid Metabolism | | Hydrolysis of lipids (5, **⇓⇓**), phosphatidylcholine (2, **↓**), triacylglycerol (2, **⇓)**; generation of prostaglandin (3, **⇑**), prostaglandin D2 (2, **⇑**); formation of eicosanoid (2, **⇑**). | 8 ⇔ |
| Embryonic Development | | Cell death of embryonic cell lines (5, **⇑**); differentiation of embryonic stem cells (4, **⇑**); colony formation of embryonic cell lines (2, **⇑**); development of embryonic cell lines (2, **⇑**); quantity of embryonic cells (2, **⇑**); transformation of embryonic cell lines (2, ⇔). | 14 **⇑** |
| Endocrine System Development & Function | | Proliferation of endocrine cell lines (3, **⇑**); growth of endocrine cell lines (2, ⇔). | 4 **↑** |
| Free Radical Scavenging | | Quantity of reactive oxygen species (4, **↑**). | 4 **↑** |
| Cellular Function & Maintenance | | Contact growth inhibition of fibroblasts (2, **⇓**); respiratory burst of phagocytes (2, ⇔). | 7 **↓** |
| Organ Development | | Angiogenesis of skin (2, ⇔); development of ear (2, **↑**); proliferation of epidermis (2, ⇔). | 5 ⇔ |
| Cellular Compromise | | Breakage of chromosomes (2, **↓**). | 7 **↓** |
| Amino Acid Metabolism | | Removal of amino acids (2, ⇔). | 2 ⇔ |
| Cell-Mediated Immune Response | | Inflammatory response (9, **↑**); proliferation of T lymphocytes (8, **↑**); inflammation (6, ⇔). | 18 **↑** |
| Humoral Immune Response | | Inflammatory response (9, **↑**); inflammation (6, ⇔). | 11 **↑** |
| Drug Metabolism | | Induction of progesterone (1, **↑**). | 1 **↑** |
| Vitamine and Mineral Metabolism | | Clearance of vitamin A (1, **↑**). | 1 **↑** |
