# Supplementary material for: Transcriptomics Comparison between Porcine Adipose and Bone Marrow Mesenchymal Stem Cells during In Vitro Osteogenic and Adipogenic Differentiation
Source: PLoS One. 2012 Mar 7;7(3):e32481. doi: 10.1371/journal.pone.0032481 (PMC3296722; doi:10.1371/journal.pone.0032481)
Supplement: Table S3 — Functional analysis results by IPA of adipogenic and osteogenic differentiation of ASC at dd21. Tabulated results from Ingenuity Pathway Analysis® (IPA) effect on function analysis of DEG between adipogenic and osteogenic differentiation of ASC at dd21. Reported are the functions sorted by decrease in significance. The category denotes the main functional category assigned by IPA. The function annotation is derived by the “effect on function” in IPA. In parenthesis are reported the number of DEG for each specific function and the arrows denote the overall effect on the function inferred by the gene annotation using IPA (⇑⇑ = highly activated in adipogenic vs. osteogenic differentiation; ⇑ = activated in adipogenic vs. osteogenic differentiation; ↑ = tends to be activated in adipogenic vs. osteogenic differentiation; ⇓⇓ = highly activated in osteogenic vs. adipogenic differentiation; ⇓ = activated in osteogenic vs. adipogenic differentiation; ↓ = tends to be activated in osteogenic vs. adipogenic differentiation.) following the criteria reported in Materials and Methods in file S1. (DOCX) [file pone.0032481.s019.docx]

### Table S3

| **Category** | **Function Annotation** | **DEG** |  |
| --- | --- | --- | --- |
| Lipid Metabolism | Metabolism of lipids (21, ****), fatty acids (8, ****); quantity of lipids (14, ****), acylglycerol (8, ****), diacylglycerol (3, ****), prostaglandin E2 (3, ****), testosterone (3, ****), acyl coenzyme A (2, ⇔); synthesis of lipids (14, ****), fatty acids (5, ****); modification of lipids (9, ****); transport of lipids (6, ****), fatty acids (4, ****), oleic acid (3, ****); uptake of fatty acids (3, ****). | 34 **** |  |
| Small Molecular Biochemestry | | Metabolic process of lipids (22, ****); metabolism of lipids (21, ****), fatty acids (8, ****); quantity of lipids (14, ****), acylglycerol (8, ****), D-glucose (7, ****), triacylglycerol (6, ****); synthesis of lipids (14, ****); transport of lipids (6, ****), fatty acids (4, ****), oleic acid (3, ****); hydrolysis of GTP (5, ****). | 49 **** |
| Cellular Growth & Proliferation | | Proliferation of cells (23, ****), tumor cell lines (10, ****), fibroblasts (5, ****), epithelial cells (4, ****); growth of cells (17, ****), tumor cell lines (9, ****); arrest in growth of cells (9, ****); induction of leukocytes (3; ****); stimulation of eukaryotic cells (3, ****). | 72 ⇔ |
| Cellular Development | | Differentiation of cell lines (18, ****), mesenchymal cells (3, ⇔); developmental disorder of cells (7, ****); adipogenesis of fibroblast cell lines (4, ****); delay in differentiation of cells (2, ****); development of bone cell lines (2, ****). | 33 **** |
| Cellular Function & Maintenance | | Polymerization of actin (5, ****); function of cytoskeleton (2, ****). | 17 **** |
| Cell Cycle | | Cell cycle progression (23, ****); cell division process of cell lines (23, ****), tumor cell lines (18, ****); cell stage of eukaryotic cells (20, ****); arrest in cell stage of cell lines (15,** **), interphase of cell lines (13, ****); mitosis (11, ****). | 30 **** |
| Molecular Transport | Quantity of lipids (14, ****), acylglycerol (8, ****), D-glucose (7, ****), triacylglycerol (6, ****), diacylglycerol (3, ****), prostaglandin E2 (3, ****), testosterone (3, ****), acyl-coenzyme A (2, ⇔); transport of lipids (6, ****), fatty acids (4, ****), oleic acid (3, ****); uptake of fatty acids (3, ****). | 28 **** |  |
| Cell Death | | Cell death (53, ****); survival of cells (20, ****), osteocytes (3, ****). | 61 **** |
