# Supplementary material for: Transcriptomics Comparison between Porcine Adipose and Bone Marrow Mesenchymal Stem Cells during In Vitro Osteogenic and Adipogenic Differentiation
Source: PLoS One. 2012 Mar 7;7(3):e32481. doi: 10.1371/journal.pone.0032481 (PMC3296722; doi:10.1371/journal.pone.0032481)
Supplement: Table S4 — Function analysis results by IPA of adipogenic and osteogenic differentiation of BMSC at dd21. Tabulated results from Ingenuity Pathway Analysis® (IPA) effect on function analysis of DEG between adipogenic and osteogenic differentiation of BMSC at dd21. Reported are the functions sorted by decrease in significance. The category denotes the main functional category assigned by IPA. The function annotation is derived by the “effect on function” in IPA. In parenthesis are reported the number of DEG for each specific function and the arrows denote the overall effect on the function inferred by the gene annotation using IPA (⇑⇑ = highly activated in adipogenic vs. osteogenic differentiation; ⇑ = activated in adipogenic vs. osteogenic differentiation; ↑ = tends to be activated in adipogenic vs. osteogenic differentiation; ⇓⇓ = highly activated in osteogenic vs. adipogenic differentiation; ⇓ = activated in osteogenic vs. adipogenic differentiation; ↓ = tends to be activated in osteogenic vs. adipogenic differentiation) following the criteria reported in Materials and Methods in file S1. (DOCX) [file pone.0032481.s020.docx]

**Table S4**

| **Category** | **Function Annotation** | **DEG** |  |
| --- | --- | --- | --- |
| Cell Death | Cell death of tumor cell lines (13, **↓**); apoptosis of normal cells (11, **↓**), endothelial cell lines (4, **⇑**), fibroblasts (4, **⇓**); survival of cell lines (7, **⇓**), osteoclasts (2, ⇔). | 18 **↓** |  |
| Cellular Movement | | Cell movement of normal cells (12, ⇔), mononuclear leukocytes (7, **⇑**), lymphocytes (6, **↑**), phagocytes (5, **↓**), T lymphocytes (5, **↑**); migration of leukocytes (8, **⇓**), endothelial cells (4, **⇑**), antigen presenting cells (3, **⇓**); invasion of tumor cell lines (6, **⇓**); arrest in cell rolling of granulocytes (2, ⇔). | 15 ⇔ |
| Lipid Metabolism | | Metabolic process of lipids (10, **⇑⇑**); metabolism of lipids (9, **⇑⇑**), fatty acids (4, **⇑**), cholesterol (3, **⇑**); quantity of lipids (9, **↑**), fatty acids (3, **↑**); synthesis of lipids (7, **⇑**); accumulation of lipids (4, **⇑⇑**). | 21 **⇑⇑** |
| Small Molecular Biochemistry | | Production of nitric oxide (5, ⇔); quantity of D-glucose (5, ⇔). | 26 ⇔ |
| Molecular Transport | | Quantity of lipids (9, **↑**), D-glucose (5, ⇔), fatty acids (3, **↑**), acyl-coenzyme A (2, ⇔), lysophospholipids (2, ⇔), prostaglandin (2, **⇑**), testosterone (2, ⇔); accumulation of lipids (4, **⇑⇑**); release of arachidonic acid (3, **⇑**), nitric oxide (3, **↓**); uptake of D-glucose (3, **↓**), fatty acids (2, **⇑**). | 15 **⇑** |
| Carbohydrate Metabolism | | Quantity of carbohydrate (6, ⇔), D-glucose (5, ⇔); uptake of D-glucose (3, **↓**). | 10 ⇔ |
| Hematological System Development & Function | Cell movement of leukocytes (9, **↓**), mononuclear leukocytes (7, **⇑**), lymphocytes (6, **↑**), phagocytes (5, **↓**), T lymphocytes (5, **↑**), macrophages (3, **↑**); activation of blood cells (8, **⇓**), leukocytes (7, **⇓**), mononuclear leukocytes (6, **⇓**), phagocytes (5, **⇓**), T lymphocytes (5, **⇓**); proliferation of T lymphocytes (7, **↑**); chemotaxis of mononuclear leukocytes (5, **↓**). | 13 **↓** |  |
| Immune Cell Trafficking | | Cell movement of leukocytes (9, **↓**), mononuclear leukocytes (7, **⇑**), lymphocytes (6, **↑**), phagocytes (5, **↓**), T lymphocytes (5, **↑**), macrophages (3, **↑**); migration of leukocytes (8, **⇓**), mononuclear leukocytes (5, **⇓**). | 13 **↓** |
| Antigen Presentation | | Immune response (9, **⇓**); inflammatory response (8, **⇓**); activation of macrophages (4, **↓**); quantity of macrophages (4, ⇔); infiltration of macrophages (3, **↑**). | 15 **↓** |
| Tissue Development | | Developmental process of connective tissue (7, **↑**), bone (6, **↑**); resorption of bone (4, **↓**); accumulation of neutrophils (3, ⇔); adhesion of endothelial cells (3, **↑**). | 11 **↑** |
| Cell-mediated Immune Response | | Immune response (9, **⇓**); inflammatory response (8, **⇓**); proliferation of T lymphocytes (7, **↑**); activation of T lymphocytes (5, **⇓**); cell movement of T lymphocytes (5, **↑**); inflammation (5, **↓**); chemotaxis of T lymphocytes (3, ⇔); migration of T lymphocytes (3, **↓**). | 16 **↓** |
| Cell-To-Cell Signaling & Interaction | | Activation of eukaryotic cells (9, **⇓**), activation of blood cells (8, **⇓**), leukocytes (7, **⇓**), mononuclear leukocytes (6, **⇓**), phagocytes (5, **⇓**), T lymphocytes (5, **⇓**), macrophages (4, **↓**); adhesion of endothelial cells (3, **↑**), phagocytes (3, **↓**); recruitment of granulocytes (3, **⇑**). | 15 **⇓** |
| Connective Tissue Development & Function | | Resorption of bone (4, **↓**); Cell movement of fibroblasts (3, **↓**); quantity of osteoclasts (3, **↓**). | 9 **↓** |
| Endocrine System Development & Function | | Quantity of testosterone (2, ⇔); release of testosterone (2, ⇔) | 4 ⇔ |
