# Supplementary material for: Transcriptomics Comparison between Porcine Adipose and Bone Marrow Mesenchymal Stem Cells during In Vitro Osteogenic and Adipogenic Differentiation
Source: PLoS One. 2012 Mar 7;7(3):e32481. doi: 10.1371/journal.pone.0032481 (PMC3296722; doi:10.1371/journal.pone.0032481)
Supplement: Table S5 — Pearson correlation between ASC and BMSC transcriptome in each pig and for each time point during adipogenic and osteogenic differentiation. All correlations were significant at p<0.0001. (DOCX) [file pone.0032481.s021.docx]

Table S5

| Time | Osteogenic | Adipogenic | Both |
| --- | --- | --- | --- |
|  | Pig 12 | | |
| All | 0.86 | 0.86 | 0.86 |
| 2 | 0.87 | 0.84 | 0.84 |
| 7 | 0.86 | 0.87 | 0.87 |
| 21 | na | 0.87 | 0.87 |
|  | Pig 22 | | |
| All | 0.74 | 0.61 | 0.69 |
| 2 | 0.84 | 0.61 | 0.73 |
| 7 | 0.67 | 0.54 | 0.61 |
| 21 | 0.82 | 0.81 | 0.81 |
|  | Pig 40 | | |
| All | 0.79 | 0.80 | 0.80 |
| 2 | 0.81 | 0.77 | 0.79 |
| 7 | 0.70 | 0.79 | 0.73 |
| 21 | 0.90 | 0.86 | 0.88 |
