# Supplementary material for: Transcriptomics Comparison between Porcine Adipose and Bone Marrow Mesenchymal Stem Cells during In Vitro Osteogenic and Adipogenic Differentiation
Source: PLoS One. 2012 Mar 7;7(3):e32481. doi: 10.1371/journal.pone.0032481 (PMC3296722; doi:10.1371/journal.pone.0032481)
Supplement: Table S6 — Pearson correlation between pigs (12, 22, and 40) transcriptome in each cell type (ASC or BMSC) during adipogenic and osteogenic differentiation. All correlations were significant at p<0.0001. (DOCX) [file pone.0032481.s022.docx]

Table S6

| Pig/Pig | Osteogenic | Adipogenic | Both |
| --- | --- | --- | --- |
|  | ASC | | |
| 12/22 | 0.83 | 0.77 | 0.81 |
| 22/40 | 0.88 | 0.80 | 0.81 |
| 12/40 | 0.84 | 0.85 | 0.84 |
|  | BMSC | | |
| 12/22 | 0.74 | 0.70 | 0.71 |
| 22/40 | 0.76 | 0.74 | 0.74 |
| 12/40 | 0.83 | 0.91 | 0.88 |
