# Supplementary material for: Transcriptomics Comparison between Porcine Adipose and Bone Marrow Mesenchymal Stem Cells during In Vitro Osteogenic and Adipogenic Differentiation
Source: PLoS One. 2012 Mar 7;7(3):e32481. doi: 10.1371/journal.pone.0032481 (PMC3296722; doi:10.1371/journal.pone.0032481)
Supplement: Table S7 — Function analysis results by IPA of BMSC and ASC during adipogenic differentiation at dd2. Tabulated results from Ingenuity Pathway Analysis® (IPA) effect on function analysis of DEG between BMSC and ASC during adipogenic differentiation at dd2. Reported are the functions sorted by decrease in significance. The category denotes the main functional category assigned by IPA. The function annotation is derived by the “effect on function” in IPA. In parenthesis are reported the number of DEG for each specific function and the arrows denote the overall effect on the function inferred by the gene annotation using IPA (⇑⇑ = highly activated in BMSC vs. ASC; ⇑ = activated in BMSC vs. ASC; ↑ = tends to be activated in BMSC vs. ASC; ⇓⇓ = highly activated in ASC vs. BMSC; ⇓ = activated in ASC vs. BMSC; ↓ = tends to be activated in ASC vs. BMSC) following the criteria reported in Materials and Methods in file S1. Effect on functions with <2 genes were discarded. (DOCX) [file pone.0032481.s023.docx]

### Table S7

| **Category** | **Function Annotation** | **DEG** |  |
| --- | --- | --- | --- |
| Cell-To-Cell Signaling & Interaction | Adhesion of cells (16, **↓**), eukaryotic cells (15, **↓**), normal cells (12, **↓**), leukocytes (8, **↓**), bone marrow cells (6, **↓**), fibroblasts (6, **↓**), endothelial cell lines (5, **↓**), mononuclear leukocytes (5, ⇔), granulocytes (4, **⇓**),T lymphocytes (4, ⇔), B lymphocytes (3, **↓**), bone cell lines (3, **↑**), monocytes (3, **↑**), neutrophils (3, **↓**), phagocytes (3, **↓**); activation of cells (13, **⇑**); binding of cells (12, **↑**), blood cells (7, **⇑**), tumor cell lines (7, **⇑**), bone marrow cells (4, **⇓**). | 22 **↓** |  |
| Cell Death | | Cell death (40, ⇔), eukaryotic cells (33, ⇔), leukocytes (13, **⇑**), mononuclear leukocytes (9, **⇑**), lymphocytes (8, **⇑**); apoptosis (36, **↓**), eukaryotic cells (30, ⇔), normal cells ( 19, ⇔), tumor cells (19, **⇓**), leukocytes (12, **↑**), bone marrow cells (6, **⇓**), phagocytes (6, **↑**), neutrophils (4, **⇑**). | 40 **↑** |
| Hematological System Development & Function | | Cell movement of leukocytes (12, **↓**), phagocytes (5, **⇓**); adhesion of leukocytes (8, **↓**), mononuclear leukocytes (5, ⇔), granulocytes (4, **⇓**),T lymphocytes (4, ⇔), monocytes (3, **↑**), neutrophils (3, **↓**), phagocytes (3, **↓**); binding of leukocytes (6, **↑**), mononuclear leukocytes (5, **⇑**), lymphocytes (4, ⇔), granulocytes (3, **⇓**), T lymphocytes (3, **↑**), phagocytes (2, **⇓**); accumulation of leukocytes (5, **⇓⇓**), granulocytes (4, **⇓⇓**), mononuclear leukocytes (3, **⇓⇓**), neutrophils (3, **⇓⇓**). | 25 **⇓** |
| Connective Tissue Development & Function | | Adhesion of fibroblasts (6, **↓**), bone marrow stromal cells (3, **↓**); adipogenesis of fibroblast cell lines (3, **⇓**); binding of adipose cell lines (2, ⇔); survival of osteoclasts (2, ⇔). | 19 **↓** |
| Tissue Development | | Adhesion of eukaryotic cells (15, **↓**), bone marrow cells (6, **↓**), endothelial cells (4, **⇓**), granulocytes (4, **⇓**),T lymphocytes (4, ⇔), B lymphocytes (3, **↓**), bone marrow stromal cells (3, **↓**), monocytes (3, **↑**), neutrophils (3, **↓**); aggregation of cells (7, **⇑**); accumulation of leukocytes (4, **⇓⇓**), granulocytes (4, **⇓⇓**), mononuclear leukocytes (3, **⇓⇓**), neutrophils (3, **⇓⇓**), phagocytes (3, **⇓**). | 29 **⇓** |
| Cell Morphology | | Morphogenesis of cells (13, **↓**), endothelial cells (2, **↓**); transformation of cell lines (9, **⇑**); cell spreading of eukaryotic cells (7, **↑**), tumor cell lines (4, ⇔); reorganization of actin (3, **↑**). | 22 **↑** |
| Cellular Movement | Migration of cells (25, **↑**), eukaryotic cells (24, ⇔), leukocytes (13, ⇔), tumor cell lines (11, **↓**), endothelial cells (8, **⇑**), mononuclear leukocytes (8, **⇑**), lymphocytes (6, **↑**), monocytes (6, **⇑**), phagocytes (5, ⇔), tumor cells (5, **↓**), eosinophils (4, **↓**), T lymphocytes (4, ⇔), antigen presenting cells (3, **⇑**). | 36 **↑** |  |
| Immuno Cell Trafficking | | Migration of leukocytes (13, ⇔), mononuclear leukocytes (8, **⇑**), monocytes (6, **⇑**), granulocytes (5, **↓**), T lymphocytes (4, ⇔); cell movement of leukocytes (11, **↓**), phagocytes (5, **⇓**); adhesion of leukocytes (8, **↓**), mononuclear leukocytes (5, ⇔), granulocytes (4, **⇓**), T lymphocytes (4, ⇔), monocytes (3, **↑**), neutrophils (3, **↓**), phagocytes (3, **↓**); accumulation of leukocytes (5, **⇓⇓**), granulocytes (4, **⇓⇓**), mononuclear leukocytes (3, **⇓⇓**), neutrophils (3, **⇓⇓**). | 18 **⇓** |
| Cardiovascular System Development & Function | | Development of blood vessel (16, **↓**), endothelial cells (3, **↓**); angiogenesis (13, **↓**); adhesion of endothelial cell lines (5, **↓**); neovascularization (5, **↓**). | 22 **↓** |
| Tumor Morphology | | Formation of malignant tumor (5, **⇓**); proliferation of cancer cells (4, **⇓⇓**). | 13 **⇓** |
| Antigen Presentation | | Immune response (11, ⇔); inflammatory response (10, **↓**); migration of macrophages (2, **↑**). | 26 **↓** |
| Cellular Development | | Differentiation of cells (25, **↓**), bone marrow cells (6, ⇔); development of cells (22, **⇓**), endothelial cells (3, **⇓**); developmental process of blood cells (15, **↑**); morphogenesis of cells (12, **⇓**); adipogenesis of fibroblast cell lines (3, **⇓**). | 39 **↓** |
| Cellular Growth & Proliferation | | Proliferation of cells (40, **↓**); growth of cells (33, **↑**), tumor cell lines (16, **⇑**); colony formation of cells (10, **↑**). | 54 **↑** |
| Gene Expression | | Transcription (28, **↓**); binding NFKB binding site (6, **↑**); activation of E box motif (4, **↓**). | 32 **↓** |
| Carbohydrate metabolism | | Binding of phosphatidylinositol 4,5-diphosphate (2, **↑**); release of proteoglycan (2, ⇔). | 3 **↑** |
| Molecular Transport | | Quantity of calcium (9, **↑**); accumulation of lipids (6, **↓**). | 16 ⇔ |
| Small Molecular Biochemistry | | Accumulation of lipids (6, **↓**); binding of phosphatidylinositol 4,5-diphosphate (2, **↑**). | 16 **↓** |
| Cell-Mediated Immune Response | | Immune response (11, ⇔); inflammatory response (11, **↓**); adhesion of T lymphocytes (4, ⇔); migration of T lymphocytes (4, ⇔); binding of T lymphocytes (3, **↑**). | 24 **↓** |
| Humoral Immune Response | | Immune response (11, ⇔); inflammatory response (11, **↓**); adhesion B lymphocytes (3, **↓**). | 23 **↓** |
| Embryonic Development | | Developmental process of embryonic tissue (8, **↑**). | 18 **↑** |
| Cellular Compromise | | Adhesion of cancer cells (4, ⇔). | 6 ⇔ |
| Cell Cycle | | Arrest in interphase of eukaryotic cells (9, ⇔), in G2 phase of eukaryotic cells (6, ⇔), in G0/G1 phase transition of tumor cell lines (3, **↓**). | 11 ⇔ |
| Tissue Morphology | | Quantity of cells (18, ⇔), stem cells (3, **↑**). | 20 ⇔ |
| Cellular Assembly & Organization | | Formation of plasma membrane projections (8, **↑**), actin stress fibers (7, **⇑**), cytoskeleton (3, **↑**); reorganization of actin (3, **↑**). | 14 **↑** |
| Lipid Metabolism | | Accumulation of lipids (6, **↓**). | 12 **↓** |
| Cellular Function & Maintenance | | Assembly of actin stress fibers (3, **⇑**); reorganization of actin (3, **↑**); respiratory burst of neutrophils (3, **↑**); assembly of fibronectin matrix (2, **⇓**). | 13 **↑** |
| DNA Replication, Recombination, & Repair | | Synthesis of DNA (11, ⇔). | 13 ⇔ |
