# Supplementary material for: Transcriptomics Comparison between Porcine Adipose and Bone Marrow Mesenchymal Stem Cells during In Vitro Osteogenic and Adipogenic Differentiation
Source: PLoS One. 2012 Mar 7;7(3):e32481. doi: 10.1371/journal.pone.0032481 (PMC3296722; doi:10.1371/journal.pone.0032481)
Supplement: Table S8 — Function analysis results by IPA of BMSC and ASC during adipogenic differentiation at dd7. Tabulated results from Ingenuity Pathway Analysis® (IPA) effect on function analysis of DEG between BMSC and ASC during adipogenic differentiation at dd7. Reported are the functions sorted by decrease in significance. The category denotes the main functional category assigned by IPA. The function annotation is derived by the “effect on function” in IPA. In parenthesis are reported the number of DEG for each specific function and the arrows denote the overall effect on the function inferred by the gene annotation using IPA (⇑⇑ = highly activated in BMSC vs. ASC; ⇑ = activated in BMSC vs. ASC; ↑ = tends to be activated in BMSC vs. ASC; ⇓⇓ = highly activated in ASC vs. BMSC; ⇓ = activated in ASC vs. BMSC; ↓ = tends to be activated in ASC vs. BMSC) following the criteria reported in Materials and Methods in file S1. Effect on functions with <2 genes were discarded. (DOCX) [file pone.0032481.s024.docx]

### Table S8

| **Category** | **Function Annotation** | **DEG** |  |
| --- | --- | --- | --- |
| Cellular Growth & Proliferation | Growth of eukaryotic cells (7, **↑**), tumor cell lines (5, **⇑**); colony formation of tumor cell lines (5, **⇑**); proliferation of tumor cell lines (5, **⇑**). | 10 **⇑** |  |
| Cell Morphology | | Shape change (5, **↑**); morphogenesis of cells (4, **↑**); extension of plasma membrane projections (3, ⇔). | 8 **↑** |
| Cellular Assembly & Organization | | Extension of plasma membrane projections (3, ⇔); assembly of actin filaments (2, **↓**). | 8 **↓** |
| Cellular Movement | | Migration of cells (6, ⇔); invasion of tumor cell lines (5, **↑**). | 10 **↑** |
| Cellular Compromise | Collapse of growth cone (2, ⇔). | 4 ⇔ |  |
| Cell Death | | Apoptosis of eukaryotic cells (7, **↓**), apoptosis of tumor cell lines (5, **↓**). | 8 **↓** |
| Cellular Development | | Differentiation of cells (7, **⇓**). | 9 **⇓** |
| Lipid Metabolism | Quantity of phosphatidic acid (2, **⇓**). | 3 **⇓** |  |
| Small Molecule Biochemistry | | Hydrolysis of GTP (2, **⇓**); Quantity phosphatidic acid (2, **⇓**); synthesis nitric oxide (2, **⇓**). | 7 **⇓** |
| Tissue Development | | Adhesion of endothelial cells (2, ⇔). | 5 ⇔ |
| Antigen Presentation | | Adhesion of monocytes (2, **↓**). | 2 **↓** |
| Cell-To-Cell Signaling & Interaction | | Adhesion of cell lines (3, **↑**), endothelial cells (2, ⇔), monocytes (2, **↓**). | 6 ⇔ |
| Hematological System Development & Function | | Adhesion of monocytes (2, **↓**); cell movement of Neutrophils (2, **⇓**). | 3 **↓** |
| Immune Cell Trafficking | | Adhesion of monocytes (2, **↓**); cell movement of Neutrophils (2, **⇓**). | 2 **↓** |
| Connective Tissue Development & Function | | Resorption of bone (2, **↓**). | 4 **↓** |
| Embryonic Development | | Differentiation of embryonic cells (2, **⇓**). | 3 **⇓** |
| Molecular Transport | | Quantity of phosphatidic acid (2, **⇓**); release of calcium (2, **⇓**). | 6 **⇓** |
| Cell Signaling | | Hydrolysis of GTP (2, **⇓**); release of calcium (2, **⇓**); synthesis of nitric oxide (2, **⇓**). | 6 **⇓** |
| Cardiovascular System Development & Function | | Adhesion of endothelial cells (2, ⇔). | 3 ⇔ |
| Cell Cycle | | Arrest in G2 phase of cell lines (2, ⇔); senescence of cells (2, ⇔). | 4 ⇔ |
| Cellular Function & maintenance | | Assembly of actin filaments (2, **↓**); organization of actin cytoskeleton (2, **↓**). | 3 **↓** |
| Gene Expression | | Activation of protein binding sites (2, **⇓**). | 4 **⇓** |
| Nucleic Acid Metabolism | | Hydrolysis of GTP (2, **⇓**). | 3 **⇓** |
| DNA Replication, Recombination, & Repair | | Hydrolysis of GTP (2, **⇓**). | 3 **⇓** |
