# Supplementary material for: Transcriptomics Comparison between Porcine Adipose and Bone Marrow Mesenchymal Stem Cells during In Vitro Osteogenic and Adipogenic Differentiation
Source: PLoS One. 2012 Mar 7;7(3):e32481. doi: 10.1371/journal.pone.0032481 (PMC3296722; doi:10.1371/journal.pone.0032481)
Supplement: Table S9 — Functional analysis results by IPA of BMSC and ASC during adipogenic differentiation at dd21. Tabulated results from Ingenuity Pathway Analysis® (IPA) effect on function analysis of DEG between BMSC and ASC during adipogenic differentiation at dd21. Reported are the functions sorted by decrease in significance. The category denotes the main functional category assigned by IPA. The function annotation is derived by the “effect on function” in IPA. In parenthesis are reported the number of DEG for each specific function and the arrows denote the overall effect on the function inferred by the gene annotation using IPA (⇑⇑ = highly activated in BMSC vs. ASC; ⇑ = activated in BMSC vs. ASC; ↑ = tends to be activated in BMSC vs. ASC; ⇓⇓ = highly activated in ASC vs. BMSC; ⇓ = activated in ASC vs. BMSC; ↓ = tends to be activated in ASC vs. BMSC) following the criteria reported in Materials and Methods in file S1. Effect on functions with <2 genes were discarded. (DOCX) [file pone.0032481.s025.docx]

### Table S9

| **Category** | **Function Annotation** | **DEG** |  |
| --- | --- | --- | --- |
| Cellular Movement | Migration of cell lines (4, ****), bone marrow cells (3, ****); chemotaxis of tumor cells (2, ****). | 4 **** |  |
| Hematopoiesis | | Migration of bone marrow cells (3, ⇔). | 3 ⇔ |
| Immuno Cell Trafficking | | Migration of bone marrow cells (3, ⇔). | 3 ⇔ |
| Cell Death | | Apoptosis of cancer cells (2, ⇔), phagocytes (2, ⇔), T lymphocytes (2, ⇔). | 5 ⇔ |
| Cell Morphology | | Shape change of fibroblast cell lines (2, ⇔). | 4 ⇔ |
| Cellular Growth & Proliferation | | Growth of eukaryotic cells (5, ****), tumor cell lines (4, ****); formation of eukaryotic cells (3, ⇔). | 5 **** |
| Carbohydrate metabolism | Uptake of D-glucose (2, ****). | 2 **** |  |
| Cardiovascular System Development & Function | | Development of blood vessel (3, ****). | 3 **** |
| Cellular Development | | Developmental process of endothelial cell lines (2, ****). | 4 **** |
| Hematological System Development & Function | | Quantity of B lymphocytes (2, ****). | 2 **** |
| Humoral Immune Response | | Quantity of B lymphocytes (2, ****). | 2 **** |
| Lipid Metabolism | | Modification of lipid (3, ****); oxidation of fatty acid (2, ⇔). | 5 **** |
| Molecular Transport | | Influx of calcium (2, ****); uptake of D-glucose (2, ****). | 5 **** |
| Small Molecule Biochemistry | | Modification of lipid (3,** ** ); oxidation of fatty acid (2, ⇔); uptake of D-glucose (2, ****). | 6 **** |
| Tissue Morphology | | Mass of connective tissue (2, ⇔); Quantity of B lymphocytes (2, ****). | 5 **** |
| Connective Tissue Development & Function | | Mass of connective tissue (2, ⇔). | 5 ⇔ |
| Cell Signaling | | Flux of Ca^2+^ (2, ⇔). | 2 ⇔ |
| Vitamin & Mineral Metabolism | | Flux of Ca^2+^ (2, ⇔). | 2 ⇔ |
