# Supplementary material for: Transcriptomics Comparison between Porcine Adipose and Bone Marrow Mesenchymal Stem Cells during In Vitro Osteogenic and Adipogenic Differentiation
Source: PLoS One. 2012 Mar 7;7(3):e32481. doi: 10.1371/journal.pone.0032481 (PMC3296722; doi:10.1371/journal.pone.0032481)
Supplement: Table S10 — Functional analysis results by IPA of BMSC and ASC during osteogenic differentiation at dd2. Tabulated results from Ingenuity Pathway Analysis® (IPA) effect on function analysis of DEG between BMSC and ASC during osteogenic differentiation at dd2. Reported are the functions sorted by decrease in significance. The category denotes the main functional category assigned by IPA. The function annotation is derived by the “effect on function” in IPA. In parenthesis are reported the number of DEG for each specific function and the arrows denote the overall effect on the function inferred by the gene annotation using IPA (⇑⇑ = highly activated in BMSC vs. ASC; ⇑ = activated in BMSC vs. ASC; ↑ = tends to be activated in BMSC vs. ASC; ⇓⇓ = highly activated in ASC vs. BMSC; ⇓ = activated in ASC vs. BMSC; ↓ = tends to be activated in ASC vs. BMSC) following the criteria reported in Materials and Methods in file S1. Effect on functions with <2 genes were discarded. (DOCX) [file pone.0032481.s026.docx]

### Table S10

| **Category** | **Function Annotation** | **DEG** |  |
| --- | --- | --- | --- |
| Cellular Compromise | Deletion of T lymphocytes (3, ****); Damage of mitochondria (2, ⇔), permeability transition of cells (2, ****). | 4 **** |  |
| Cell Death | | Apoptosis of cell lines (15, ****); cell viability of eukaryotic cells (6, ****); apoptosis of neutrophils (4, ****), bone cell lines (3, ****), endothelial cell lines (3,** **), osteocytes (3, ****), osteoblasts (2, ⇔), osteoclasts (2, ****), stem cells (2, ****); cell death of microvascular endothelial cells (2, ⇔); killing of T lymphocytes (2, ⇔); lysis of blood cells (2, ****). | 19 **** |
| Connective Tissue Development & Function | | Binding of fibroblasts (2, ****); thickness of connective tissue (2, ****). | 6 ⇔ |
| Tumor Morphology | | Killing of tumor cells (2, ⇔); mass of tumor tissue (1, ****). | 2 ⇔ |
| Cardiovascular System Development & Function | | Development of blood vessel (8, ****); formation of endothelial tube (3, ****); cell-cell contact of endothelial cell lines (2, ****); growth of endothelial cell lines (2, ⇔). | 12 **** |
| Cellular Movement | | Migration of cells (16, ****, eukaryotic cells (14, ****), mononuclear leukocytes (5, ****), endothelial cell lines (4, ⇔), monocytes (4, ****), T lymphocytes (4, ⇔), phagocytes (3, ****), B lymphocytes (2, ⇔), eosinophils (2, ⇔); invasion of cell lines (10, ****), tumor cell lines (8, ****); cell movement of mononuclear leukocytes (6, ****), lymphocytes (5, ⇔), monocytes (4, ****), phagocytes (4, ****), T lymphocytes (4, ⇔); chemotaxis of T lymphocytes (3, ⇔); infiltration of lymphocytes (3, ****). | 19 **** |
| Cell growth & proliferation | | Growth of epithelial cell lines (4, ⇔), bone marrow cells (3, ****), endothelial cell lines (2, ****); inhibition of T lymphocytes (3, ****). | 8 **** |
| Hematologycal System Development & Function | | Cell movement of mononuclear leukocytes (6, ⇔), lymphocytes (5, ****), monocytes (4, ****), phagocytes (4, ****), T lymphocytes (4, ⇔); adhesion of T lymphocytes (4, ****); transmigration of leukocytes (4, ****), monocytes (2, ****), T lymphocytes (2, ****); binding of lymphocytes (3, ****); chemotaxis of T lymphocytes (3, ⇔); infiltration of lymphocytes (3, ****); inhibition of T lymphocytes (3, ****); transmigration of mononuclear leukocytes (3, ****); adhesion of peripheral blood leukocytes (2, ⇔); mobilization of granulocytes (2, ⇔), mononuclear leukocytes (2, ⇔). | 8 **** |
| Immune cell trafficking | | Cell movement of mononuclear leukocytes (6, ⇔), lymphocytes (5, ****), monocytes (4, ****), phagocytes (4, ****), T lymphocytes (4, ⇔); migration of mononuclear leukocytes (5, ****), monocytes (4, ****), T lymphocytes (4, ⇔), B lymphocytes (2, ⇔), eosinophils (2, ⇔); adhesion of T lymphocytes (4, ****); transmigration of leukocytes (4, ****), chemotaxis of T lymphocytes (3, ⇔); infiltration of lymphocytes (3, ****). | 9 **** |
| Endocrine System Development & Function | | Binding of estrogen (2, ****); survival of endocrine cells (2, ****). | 4 **** |
| Gene expression | | Activation of HNF4 binding site (2, ⇔), insulin response element (2, ⇔). | 4 ⇔ |
| Lipid Metabolism | | Hydrolysis of phosphatidic acid (3, ****); binding of estrogen (2,** **); biosynthesis of cholesterol (2, ****); exposure of phospholipid (2,** **); generation of prostaglandin E2 (2, ****); phospholipid flip-flop of phosphatidylserine (2, ⇔); quantity of phosphatidylcoline (2, ****); reduction of fatty acid (2, ****). | 14 **** |
| Molecular transport | | Quantity of cyclin GMP (2, ****); quantity of phosphatidylcoline (2, ****). | 9 **** |
| Small Molecular Biochemistry | | Hydrolysis of phosphatidic acid (3,** **); binding of estrogen (2, ****), hormone (2, ****); biosynthesis of cholesterol (2, ****); exposure of phospholipid (2,** **); generation of prostaglandin E2 (2, ****); phospholipid flip-flop of phosphatidylserine (2, ⇔); Quantity of cyclic GMP (2, ****); quantity of phosphatidylcoline (2, ****). | 16 **** |
| Cell Morphology | | Polarization of B lymphocytes (2, ⇔), polarization of phagocytes (2, ⇔). | 11 ⇔ |
| Cell-To-Cell Signaling & Interaction | | Adhesion of T lymphocytes (4, ****); binding of lymphocytes (3, ****), fibroblasts (2, ****); attraction of mononuclear leukocytes (2, ⇔); gap junctional intercellular communication of cell line (2, ****). | 11 **** |
| Cell-Mediated Immune Response | | Adhesion of T lymphocytes (4, ****); cell movement of T lymphocytes (4, ⇔); migration of T lymphocytes (4, ⇔); chemotaxis of T lymphocytes (3, ⇔). | 6 ⇔ |
| Tissue Development | | Adhesion of T lymphocytes (4, ****); formation of endothelial tube (3, ****). | 12 ⇔ |
| Cellular Development | | Developmental process of bone cell lines (4, ****), bone marrow-derived macrophages (2, ⇔); growth of epithelial cell lines (4, ⇔), bone marrow cells (3, ****); osteoclastogenesis of macrophages (2, ⇔). | 9 **** |
| Antigen Presentation | | Cell movement of monocytes (4, ****), transmigration of monocytes (2, ****). | 5 **** |
| Carbohydrate Metabolism | | Hydrolysis of phosphatidic acid (3, ****); phospholipid flip-flop of phosphatidylserine (2, ⇔); redistribution of phosphatidic acid (2, ⇔). | 8 **** |
| Humoral Immune Response | | Migration of B lymphocytes (2, ⇔); polarization of B lymphocytes (2, ⇔). | 3 ⇔ |
| Hematopoiesis | | Migration of megakaryocytes (2, ⇔). | 2 ⇔ |
| Cellular function and maintenance | | Depolarization of mitochondrial membrane (2, ⇔); organization of normal cells (2, ****); regulation of blood cells (2, ****). | 5 **** |
| Drug Metabolism | | Generation of prostaglandin E2 (2, ****). | 3 **** |
| Cellular Assembly & organization | | Depolarization of mitochondrial membrane (2, ⇔). | 8 ⇔ |
| Tissue Morphology | | Loss of bone (2, ****); thickness of connective tissue (2, ****). | 7 **** |
| Cell Cycle | | Cell division process of leukocytes cell lines (3, ****). | 4 **** |
| Nucleic Acid Metabolism | | Quantity of cyclic GMP (2, ****). | 3 **** |
