# Supplementary material for: Transcriptomics Comparison between Porcine Adipose and Bone Marrow Mesenchymal Stem Cells during In Vitro Osteogenic and Adipogenic Differentiation
Source: PLoS One. 2012 Mar 7;7(3):e32481. doi: 10.1371/journal.pone.0032481 (PMC3296722; doi:10.1371/journal.pone.0032481)
Supplement: Table S11 — Functional analysis results by IPA of BMSC and ASC during osteogenic differentiation at dd21. Reported are the functions sorted by decrease in significance. The function annotation is derived by the “effect on function” in IPA. In parenthesis are reported the number of DEG for each specific function and the arrows denote the overall effect on the function inferred by the gene annotation using IPA (⇑⇑ = highly activated in BMSC vs. ASC; ⇑ = activated in BMSC vs. ASC; ↑ = tends to be activated in BMSC vs. ASC; ⇓⇓ = highly activated in ASC vs. BMSC; ⇓ = activated in ASC vs. BMSC; ↓ = tends to be activated in ASC vs. BMSC) following the criteria reported in Materials and Methods in file S1. Effect on functions with <2 genes were discarded. (DOCX) [file pone.0032481.s027.docx]

### Table S11

| **Category** | **Function Annotation** | **DEG** |  |
| --- | --- | --- | --- |
| Antigen Presentation | Inflammatory response (6, ****); cell movement of macrophages (2, ⇔). | 6 **** |  |
| Cell-mediated Immune Response | | Inflammatory response (6, ****); adhesion of T lymphocytes (2, ****); chemotaxis of T lymphocytes (2, ⇔); migration of T lymphocytes (2, ****). | 7 **** |
| Humoral Immuno Response | | Inflammatory response (6, ****). | 6 **** |
| Cellular Movement | | Migration of normal cells (5, ****), leukocytes (4, ⇔), endothelial cells (3, ****), monocytes (3, ****); chemotaxis of normal cells (4, ⇔), mononuclear leukocytes (3, ****), T lymphocytes (2, ⇔); invasion of tumor cell lines (4, ⇔), normal cells (3, ⇔); cell movement of macrophages (2, ⇔). | 7 **** |
| Immuno Cell Trafficking | | Migration of leukocytes (4, ⇔), monocytes (3, ****), T lymphocytes (2, ****); Chemotaxis of mononuclear leukocytes (3, ****), T lymphocytes (2, ⇔); activation of phagocytes (2,** **); adhesion of T lymphocytes (2, ****). | 4 **** |
| Cellular Assembly & Organization | | Formation of lamellipodia (3, ****); assembly of actin filaments (2, ⇔); rearrangement of cytoskeleton (2, ⇔). | 5 ⇔ |
| Cardiovascular System Development & Function | Angiogenesis (4,** **); cardiovascular process of tissue (3, ****); adhesion of endothelia cells (2, ⇔). | 6 **** |  |
| Cellular Development | | Angiogenesis of endothelial cells (2, ****); Differentiation of bone cell lines (2, ****). | 4 **** |
| Cell-To-Cell Signaling & Interaction | | Adhesion of cell lines (3, ****), endothelial cells (2, ⇔), epithelial cell lines (2, ****), fibroblast cell lines (2, ****), T lymphocytes (2, ****); attachment of cell (3, ⇔); activation of phagocytes (2, ****). | 5 **** |
| Tissue development | | Developmental process of tissue (7, ⇔); adhesion of endothelial cells (2, ⇔), T lymphocytes (2, ****). | 7 ⇔ |
| Embryonic Development | | Adhesion of embryonic cell lines (2, ****). | 3 **** |
| Cell Death | | Cell death of normal cells (6, ****); apoptosis of normal cells (5, ⇔), endothelial cell lines (2, ⇔), epithelial cells (2, ⇔), fibroblasts (2, ⇔). | 6 ⇔ |
| Cellular Growth & Proliferation | | Proliferation of eukaryotic cells (7, ****), connective tissue cells (3, ****), leukocytes cell lines (2, ****); growth of tumor cells (2, ****). | 8 ⇔ |
| Hematological System Development & Function | | Chemotaxis of mononuclear leukocytes (3, ****); activation of phagocytes (2, ****); adhesion of T lymphocytes (2, ****); cell movement of macrophages (2, ⇔). | 4 **** |
| Connective Tissue Development & Function | | Adhesion of fibroblast cell lines (2, ****). | 4 **** |
| Cell Morphology | | Shape change (4, ⇔), normal cells (3, ****); cell spreading of eukaryotic cells (3, ****), cell lines (2, ****); reorganization of cytoskeleton (2, ⇔). | 5 **** |
| Cellular Function & Maintenance | | Assembly of actin filaments (2, ****); reorganization of cytoskeleton (2, ⇔). | 4 **** |
| Small Molecular Biochemistry | | Production of nitric oxide (2, ⇔); release of nitric oxide (2, ⇔). | 5 ⇔ |
| Cell Signaling | | Production of nitric oxide (2, ⇔); release of calcium (2, ****), nitric oxide (2, ⇔). | 4 **** |
| Molecular Transport | | Release of calcium (2, ****), nitric oxide (2, ⇔). | 5 **** |
| Carbohydrate Metabolism | | Binding of carbohydrate (2, ****). | 3 **** |
| Tumor Morphology | | Growth of tumor (2, ⇔). | 2 ⇔ |
| Gene Expression | | Activation of AP1 consensus site (2, ****). | 2 **** |
